# The impact of particle size on PFAS concentrations in dust from homes in North Carolina and New York and implications for exposure

**DOI:** 10.1080/02786826.2025.2582532

**Published:** 2025-11-18

**Authors:** Clara M. A. Eichler, Mahender Singh Rawat, Naomi Y. Chang, Elizabeth Brown, Sujan Fernando, Thomas M. Holsen, Glenn C. Morrison, Andrea R. Ferro, Barbara J. Turpin

**Affiliations:** a Department of Environmental Sciences and Engineering, The University of North Carolina at Chapel Hill, Chapel Hill, North Carolina, USA; b Department of Civil and Environmental Engineering, Clarkson University, Potsdam, New York, USA; c Center for Air and Aquatic Resources Engineering and Science, Clarkson University, Potsdam, New York, USA

## Abstract

Per- and polyfluoroalkyl substances (PFAS) are manufactured chemicals and ubiquitously present in the environment, including in homes. The two major exposure pathways for PFAS indoors are inhalation and accidental ingestion of house dust; however, the influence of dust particle size on PFAS exposure is not well understood to date. Thus, we are aiming to better understand the relationship between dust particle size and PFAS concentrations. We collected dust from 10 homes in North Carolina and seven homes in New York, sieved the dust into multiple size fractions ranging from <63 μm to <2,000 μm, and used targeted methods to analyze the fractions for PFAS. We found that many neutral PFAS are significantly (*p* < 0.05) and negatively correlated with dust particle size (mean Pearson correlation coefficient r=−0.70 to −0.90), i.e., higher concentrations were found in the smaller size fractions. This suggests that neutral PFAS concentrations in dust are primarily influenced by partitioning to the dust particles from the gas phase. On the other hand, several perfluoroalkyl acids showed no clear or positive correlations between particle size and concentration (mean Pearson r=−0.45 to 0.65), suggesting that additional migration pathways contribute preferentially to the larger size fractions, such as abrasion of fibers from upholstery. Dust-air partition coefficients, $K_{d}^{\prime }$, derived for neutral PFAS for a subset of homes reflect this observation, with higher $\log(K_{d}^{\prime })$ values found for smaller dust size fractions compared to larger size fractions. This work highlights the importance of the choice of size fraction when analyzing PFAS in dust and for exposure assessments.

## Graphical Abstract

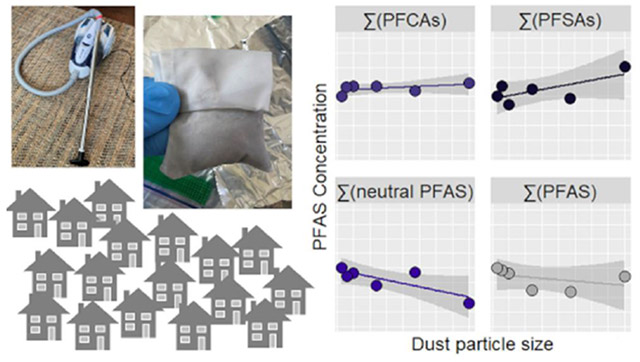

## Introduction

Per- and polyfluoroalkyl substances (PFAS) are ubiquitous, manufactured chemicals that are present in many consumer and industrial products because of their water- and grease-resistant and surfactant properties (Glüge et al. 2020; Sunderland et al. 2019; Sznajder-Katarzynska et al. 2019). Due to their persistence in the environment, PFAS are also known as “forever chemicals” (Brunn et al. 2023; Cordner et al. 2021). Many PFAS have been associated with negative health impacts, including thyroid disease, liver damage, kidney and testicular cancer, reduced response to vaccines in children, and lower birth weight (Fenton et al. 2021). However, of the thousands of PFAS present in commerce, sufficient health data to assess their toxicity are available only for a handful of “legacy” PFAS, e.g., perfluorooctanoic acid (PFOA) and perfluorooctanesulfonic acid (PFOS). While use of several PFAS has already been limited in many countries (Fenton et al. 2021; Pelch et al. 2022), legacy and substitute PFAS can persist for years in indoor environments as a result of their use in durable goods and partitioning to indoor reservoirs. Their known or suspected toxicity and ubiquity in the environment and in products of everyday use, including food packaging (Minet et al. 2022; Phelps et al. 2024; Yuan et al. 2016), personal care products (Harris et al. 2022), cosmetics (Harris et al. 2022; Schultes et al. 2018), clothing (Eichler et al. 2024b; Gremmel et al. 2016; Xia et al. 2022), and furniture (Robel et al. 2017; Rodgers et al. 2022) among others, make comprehensive risk assessments of PFAS paramount.

When assessing human exposure to PFAS, indoor environments are of particular importance. People spend about 90% of their time indoors (Klepeis et al. 2001) where PFAS-containing products are frequently stored and used (Glüge et al. 2020). Settled house dust is often investigated to gain insights into the presence and levels of chemicals of concern indoors (Liu 2022; Salthammer et al. 2018; Weschler and Nazaroff 2010), including PFAS (DeLuca et al. 2022; Savvaides et al. 2021; Young et al. 2021a), phthalates (Bi et al. 2018; Wang et al. 2013b), polycyclic aromatic hydrocarbons (PAHs) (Cao et al. 2019; Lewis et al. 1999), polychlorinated biphenyls (PCBs) (Wang et al. 2013a), and flame retardants (FRs) (Al-Omran and Harrad 2016), because dust is a common indoor reservoir and relatively easy to sample. Further, accidental dust ingestion, transdermal uptake from dust particles adhered to skin, and inhalation of resuspended dust particles are important exposure pathways specifically for young children, who are often most vulnerable to the harmful effects of these chemicals (DeLuca et al. 2022; Eichler et al. 2021; Holder et al. 2023). PFAS have been detected in house dust and in dust from occupational settings in the United States (US) (DeLuca et al. 2024; Fraser et al. 2013; Hall et al. 2020; Karâskovâ et al. 2016; Kato et al. 2009; Knobeloch et al. 2012; Proctor et al. 2025; Schildroth et al. 2022; Strynar and Lindstrom 2008; Wu et al. 2020; Young et al. 2021a; Zheng et al. 2020), Canada (Beesoon et al. 2012; Kubwabo et al. 2005; Shoeib et al. 2005; 2011), Europe (Björklund et al. 2009; Ericson Jogston et al. 2012; Fromme et al. 2008; Harrad et al. 2019; Haug et al. 2011; Huber et al. 2011; Karâskovâ et al. 2016; Simonetti et al. 2020; Winkens et al. 2018; Xu et al. 2013), Asia (Su et al. 2016; Tian et al. 2016; Wang et al. 2022; Xu et al. 2021; Yao et al. 2018), Africa (Shoeib et al. 2016), and Australia (Li et al. 2024). Ionic/ionizable PFAS like PFOA and PFOS are most commonly included in targeted PFAS analyses, and neutral PFAS like fluorotelomer alcohols (FTOHs) and perfluorooctane sulfonamidoethanols (FOSEs) are also frequently targeted. More recently, high concentrations of polyfluoroalkyl phosphate esters (PAPs) have been found in dust samples and sparked increased interest in this PFAS sub-class (Eriksson and Kärman 2015; Savvaides et al. 2021). In addition, measurements of total organic fluorine (TOF) (Young et al. 2021b; 2022) as a proxy for the presence of PFAS in dust have been done, while non-targeted (Newton et al. 2025) and suspect-screening analysis methods (Newton et al. 2020) for environmental samples including dust are still in development.

House dust is a complex mixture that contains organic materials shed by humans, animals, and plants, as well as organic and inorganic particulate matter from settled airborne particles of different origins and tracked in by occupants (Morawska and Salthammer 2003; USEPA 2017). House dust can also contain particles and fibers shed from mechanical abrasion of flooring, clothing, and other objects as well as bacteria and fungal material from microbial growth in the dust (Dannemiller et al. 2017). The composition of dust can vary greatly based on the geographic location, building properties, occupant habits, and characteristics of the indoor environment (Morawska and Salthammer 2003). House dust includes particles ranging in size from <2.5 μm to several mm, however, particles >30 μm have been classified as settled house dust (Morawska 2004), and size fractions in the range of <50–75 μm to 2 mm are frequently used for chemical analysis (Morawska and Salthammer 2003). For exposure assessments of lead in dust, size fractions of <150 μm have been identified as of particular interest, because particles in this size fraction are more likely to adhere to hands and thus ingestion *via* hand-to-mouth transfer and transdermal uptake is more likely (USEPA 2016). This recommendation has been followed for other chemicals as well (Al-Omran et al. 2021). In addition, gastro-intestinal bioaccessibility of several chemicals has been found to be higher if the chemicals are associated with smaller particles (Rusina et al. 2023; Wang et al. 2013b). Particle size also affects inhalation exposure to chemicals in resuspended dust, as particle size impacts the resuspension fraction (i.e., the fraction of settled dust particles that is resuspended by an external force, such as a footstep), the particle settling velocity in the air, as well as the deposition in the respiratory tract (Qian et al. 2014).

Nevertheless, there is great inconsistency in the dust size fractions that have been analyzed for PFAS to date. Multiple studies used dust sieved to <150 μm (Ericson Jogston et al. 2012; Eriksson and Kärman 2015; Hall et al. 2020; Kubwabo et al. 2005; Schildroth et al. 2022; Shoeib et al. 2005; 2011; Strynar and Lindstrom 2008; Wang et al. 2022; Wu et al. 2020; Yao et al. 2018; Young et al. 2021a), while others used the <125 μm fraction (Xu et al. 2021), the <250 μm fraction (Shoeib et al. 2016), the <500 μm fraction (Eichler et al. 2024a; Fraser et al. 2013; Goosey and Harrad 2011; Hall et al. 2020; Harrad et al. 2019; Proctor et al. 2025; Winkens et al. 2018), the <1 mm or <2 mm fraction (Kato et al. 2009; Knobeloch et al. 2012; Li et al. 2024), or the entire dust sample (Haug et al. 2011; Huber et al. 2011; Su et al. 2016; Zheng et al. 2020). The use of different size fractions makes it difficult to compare concentrations as well as resulting exposure estimates. To date, only one study investigated different size fractions (1–2 mm, 390 μm-1 mm, 190–390 *μ*m, 75–190 *μ*m, 25–75 *μ*m, <25 μm and <3.7 μm) of a composite dust sample that consisted of the combined content of 32 vacuum cleaner bags from homes in Stockholm, Sweden (Gustafsson et al. 2022; Gustafsson et al. 2018). In their study, the authors analyzed the dust for several ionic PFAS and PAPs, and observed a general trend of highest concentrations in the smallest size fraction (<3.7 μm) (Gustafsson et al. 2022). However, for several PFAS, including perfluorohexanoic acid (PFHxA), PFOA, and PFOS, the 1–2 mm fraction yielded concentrations that are similarly high as those in the <3.7 μm fraction (Gustafsson et al. 2022). For perfluorodecanoic acid (PFDA), the concentration in the largest size fraction was even higher than the concentration in the smallest fraction (Gustafsson et al. 2022). These observations indicate that the choice of size fraction could be important for subsequent exposure assessments, as well as potentially yield insights into the sources of PFAS in dust and the importance of dust as an indoor reservoir for PFAS. Further that the relationship between the size fraction and concentration varies for different PFAS, making the choice of the most appropriate size fraction to include in exposure assessments even more difficult. However, the study included only a limited number of PFAS and PFAS sub-classes, and further research is needed to understand the impact of particle size fraction on PFAS concentrations in dust.

The relationship between dust particle size and concentrations of different volatile and semi-volatile organic compounds (VOCs and SVOCs) has been studied for brominated flame retardants (BFRs), PAHs, PCBs, pesticides, and phthalates (Ali 2024; Cao et al. 2014; 2019; He et al. 2018; Kajiwara and Takigami 2016; Lewis et al. 1999; Wang et al. 2013a; 2013b; Wei et al. 2009). Concentrations of PAHs, pesticides, and phthalates generally seem to increase with decreasing particle size (Ali 2024; Lewis et al. 1999; Wang et al. 2013b), however, some variability in this pattern has been observed for dust from different microenvironments (Ali 2024; Cao et al. 2019). For BFRs, which have been studied more extensively, higher concentrations in smaller dust particles have been reported in some studies (He et al. 2018; Wei et al. 2009), while other studies observed peak concentrations in mid-range to higher-range particle size fractions, often also depending on the class of BFR and the microenvironment (Al-Omran and Harrad 2016; Cao et al. 2014; Kajiwara and Takigami 2016; Zhou and Püttmann 2019). Based on the discussions provided in these studies, the distribution of organic chemicals in different size fractions is impacted by several factors. The surface area-to-volume ratio of the particles, which is higher for smaller particles, affects partitioning of surface-active compounds to the particle surface (Lewis et al. 1999; Wei et al. 2009) as well as absorption of compounds into the particle (Cao et al. 2014). The organic content of the particles, which influences the absorption behavior of hydrophobic chemicals, is another factor (Cao et al. 2014; He et al. 2018), although organic content alone has been found to be insufficient to explain observed variations in concentrations (Al-Omran and Harrad 2016; Liu and Folk 2021). In addition, the predominating migration pathways of the chemical into the dust, which can vary by microenvironment, chemical structure, and chemical properties, have been investigated to explain varying concentrations in different dust size fractions (Cao et al. 2014; He et al. 2018; Kajiwara and Takigami 2016; Rauert and Harrad 2015; Webster et al. 2009; Wei et al. 2009; Zhou and Püttmann 2019).

The pathways for VOCs and SVOCs to migrate from a source (usually a consumer product or building material) into settled dust are 1) emission from the source into the gas phase followed by partitioning from the gas phase to the dust, 2) abrasion of particles or fibers from the source followed by transfer of these particles or fibers to the dust, and 3) transfer from the source to the dust by direct contact (Bi et al. 2021; Cao et al. 2014; Rauert et al. 2015; Webster et al. 2009). Depending on the presence or absence of any of these pathways, the relationship between particle size and chemical concentration might vary greatly (Al-Omran et al. 2021). Transfer *via* direct contact has been shown to result in much higher concentrations than emission with subsequent dust-air partitioning (Liu and Folk 2021; Rauert et al. 2016; Rauert and Harrad 2015). Abrasion due to wear and weathering can also result in high concentrations in the dust (Rauert et al. 2014; Rauert and Harrad 2015), however, in the absence of transfer *via* direct contact and abrasion, which requires specific types of sources and chemical uses, accumulation of VOCs and SVOCs in dust due to emission into air and subsequent partitioning likely explains the presence of the majority of detected compounds (Weschler et al. 2008; Weschler and Nazaroff 2010).

With these considerations and knowledge gaps in mind, this work had the following specific objectives: (1) To measure a broad range of targeted PFAS and, where possible, extractable organic fluorine (EOF) in dust from homes from two different US locations, (2) to compare PFAS occurrences and concentrations in different dust size fractions, (3) to investigate the relationship between particle size and concentrations, and (4) to derive size-dependent dust-air partition coefficients for a subset of homes and PFAS. These objectives will allow us to evaluate the influence of particle size on PFAS occurrence, fate, and exposure. Further, the results of this work will advance our understanding of the role of different PFAS migration pathways from sources to dust, guide future sampling campaigns in their choice of particle size to use, and provide insights in the importance of different size fractions for exposure assessments.

## Materials and methods

### Chemicals

All dust samples were analyzed for nine neutral PFAS, specifically three FTOHs, two FTACs, two FOSEs, two FOSAs at the University of North Carolina at Chapel Hill (UNC-CH). Two different methods were used to analyze the dust samples for ionic/ionizable PFAS. The method used at UNC-CH included 26 ionic/ionizable PFAS and the method used at Clarkson University (Clarkson) included 38 ionic/ionizable PFAS. The ionic/ionizable PFAS that were part of both analytical methods included 11 perfluorocarboxylic acids (PFCAs), eight perfluorosulfonic acids (PFSAs), and GenX. At UNC-CH, two additional PFCAs and four PAPs were targeted. At Clarkson, linear and branched perfluorohexane sulfonic acid (PFHxS) and PFOS, respectively, were targeted separately. In addition, seven emerging PFAS, three fluorotelomer carboxylic acids (FTCAs), three fluorotelomer sulfonic acids (FTS), two perfluorooctane sulfonamidoacetic acids (FOSAAs), as well as perfluorooctane sulfonamide (FOSA) were included in the Clarkson method. A complete list of native and mass-labeled PFAS used in the targeted analyses and the vendors of the standards used can be found in Table S1 the Supplemental Information (SI). All other solvents and reagents used in the analyses done at UNC-CH were HPLC grade and purchased from Thermo Fisher Scientific (Waltham, MA, USA). Solvents and reagents used for the analyses at Clarkson were LC/MS grade and obtained from Thermo Fisher Scientific chemicals (Waltham, MA, USA).

### Dust sampling

House dust samples were collected from a convenience sample of 10 detached, single-family, nonsmoking homes in the Chapel Hill/Durham area in North Carolina (NC) (Eichler et al. 2023) and seven homes of the same type in Potsdam, New York (NY). Sampling in NC was done in 2022 as part of the Indoor PFAS Assessment (IPA) Campaign; details describing the IPA Campaign homes, sampling procedures, and analytical methods can be found elsewhere (Chang et al. 2025a; 2025b; Eichler et al. 2023; 2024a). Briefly, dust was collected by vacuuming the floor of the main living area of each home using a vacuum cleaner and a sampling sock (Nylon Filter Bags, 25 μm mesh, 1.75 inches x 5 inches, Dulytek, online) placed inside the hose behind the vacuum cleaner nozzle. Occupants were asked to refrain from vacuuming the space for at least one week prior to sampling. Rugs and carpets, if present, were included in the sampling. Depending on the vacuumed floor area and dust loading, one to five sampling socks per home were collected. After sampling, each sampling sock filled with dust was wrapped individually in clean aluminum foil and stored inside a polypropylene (PP) zipper bag at −80 °C until analysis. Additional sampling socks were brought into the field, installed inside the vacuum cleaner hose, and then removed immediately. These socks were otherwise treated like the actual samples and collected as field blanks (FBs). The 10 NC dust samples included here were collected after 6 months of study participation of each IPA Campaign home (home IDs 01, 10, 18, 30, 35, 43, 50, 59, 65, and 78). Further, 12 FBs total were collected from the NC homes at *t* = 6 months.

Sampling at a convenience sample of seven NY homes (home IDs C1-C7) was conducted in 2023 and followed the same protocol as outlined above. However, in order to collect a sufficient amount of dust to allow for EOF analysis in addition to targeted PFAS analysis, multiple rooms in each NY homes were vacuumed in addition to the main living area. Again, occupants were asked not to vacuum for at least one week prior to sampling. We collected seven FBs from the NY homes. Samples and FBs were stored at −20 °C until further processing and analysis.

### Dust sample processing and analysis

For sieving and extraction, the dust was removed from the sampling sock. If multiple sampling socks were filled during sampling at one home, the contents were combined and sieved together prior to extraction. Each of the NC dust samples from eight homes (homes 10, 18, 30, 35, 50, 59, 65, and 78) were sieved into two size fractions, 250–500 μm and <250 μm, because not enough dust was available to include additional size fractions. The dust samples from homes 01 and 43 were each sieved into four size fractions: 250–500 μm, 125–250 μm, 63–125 μm, and <63 μm. About 100 mg of each size fraction was then extracted and analyzed by gas chromatography-electron impact-mass spectrometry (GC-EI-MS) for nine neutral PFAS, while another 100 mg of each size fraction was extracted separately and analyzed by ultra-high performance liquid chromatography-electrospray ionization tandem mass spectrometry (UHPLC-ESI-MS/MS) for 26 ionic/ionizable PFAS at UNC-CH. Details regarding the extraction and analytical method used for neutral PFAS analysis can be found in Section S1 the SI and in Eichler et al. (2024a). The extraction and analytical methods used for ionic/ionizable PFAS at UNC-CH is described in Section S2 and in Chang et al. (2025b). If a sufficient amount of dust was left after extraction for GC-MS and LC-MS/MS analysis, it was analyzed for EOF as described below. A small subset of NC dust samples was also analyzed for ionic/ionizable PFAS at Clarkson as described in Section S3 and the results are further discussed in Section S4.

Each of the NY dust samples was sieved into six size fractions: >1,180 μm, 600–1,180 μm, 250–600 μm, 150–250 μm, 75–150 μm, and <75 μm. About 100 mg of each size fraction was extracted and analyzed at UNC-CH for neutral PFAS (Section S1). In addition, 10–15 mg of each size fraction was extracted and analyzed for ionic/ionizable PFAS at Clarkson (Section S3). For EOF analysis, about 0.05 g of each size fraction was needed. Only the size fractions >1,180 μm, 150–250 μm, 75–150 μm, and <75 μm had enough dust available for EOF analysis. EOF analysis was conducted using combustion ion chromatography (CIC) after extraction of the dust samples in methanol and extract clean-up with solid-phase extraction (SPE) cartridges. A detailed description of the EOF extraction and analysis method can be found in Section S5.

### Neutral PFAS in the gas phase

Sampling of neutral PFAS in the gas phase at the NC homes has been described in detail in Eichler et al. (2023). In brief, gas-phase samples were collected in the main living area of each NC home for ~72 h (~5 L/min sampling flowrate, 21.2 m^3^ sampling volume) using polyurethane foam (PUF)-XAD2-PUF sandwich cartridges (ORBO 1500 Precleaned Small PUF/Amberlite XAD-2/PUF Cartridge, Supelco, Bellefonte, PA) downstream of a quartz-fiber filter (QFF; Catalog No. 21038, Supelco, Bellefonte, PA; prebaked at 550 °C for 12 h). PUF plugs and XAD2 were spiked with mass-labeled standards and extracted twice by ultrasonication in a 3:1 (v/v) hexane/methanol mixtures. Extracts were combined, cleaned up with ENVI-Carb (Supelclean ENVI-Carb SPE Bulk Packing, Supelco, Bellefonte, PA), and concentrated under nitrogen to ~1 mL. Extracts were transferred to GC vials and analyzed following the UNC-CH protocol for neutral PFAS. The gas-phase samples were collected in the week prior to dust sampling. No gas-phase sampling took place at the NY homes.

### QA/qc

Solvent blanks, FBs (i.e., unused sampling socks that were brought into the field), and lab blanks were included in all extraction batches for the targeted analyses and processed like samples to monitor for contamination. Of the 12 FBs collected from the NC homes, four were analyzed by GC-MS and eight by LC-MS/MS using the UNC-CH method. PFAS concentrations in all FBs were calculated by dividing the measured PFAS mass in the FB by the average mass of dust extracted for each analytical method. It has to be noted that extraction of the actual dust samples did not include extraction of the sampling sock.

Method detection limits (MDLs) for each of the targeted analysis methods are reported in Tables S3-S5. Additional information on MDL determination, recoveries, and analytical precision can be found in the method descriptions in the SI (Sections S1-S5, Figure S1).

### Data analysis

We calculated the concentrations in the <600 μm (NY homes) and <500 μm (NC homes) as weighted sums by adding the products of the weight fraction of each particle size fraction and the concentrations of each PFAS together for each home (Section S6). For the statistical analysis of the linear relationship between PFAS concentrations in dust and particle size fraction, the concentration data were normalized by dividing the concentration of each PFAS in the larger size fractions (>75 μm for NY homes or >63 μm for NC homes) by the concentration of the same PFAS in the <75 μm (NY homes) or <63 μm (NC homes) fraction for each home. This method was chosen because concentrations among homes ranged over nearly two orders of magnitude. By normalizing to the same size fraction within a home, data among homes can be combined to test for size-fraction trends. Non-detects were replaced by MDL/2. The normalized concentrations were ln-transformed to achieve more normal distributions (Figure S2). Then, Pearson correlation coefficients (r) and *p* values were calculated for each home and PFAS. To calculate average correlation coefficients, the r values were Fisher-transformed, averaged, and then transformed back, as described in Corey et al. (1998). The correlation analysis included data from all seven NY homes as well as from NC homes 01 and 43. Further, only PFAS with detection frequencies (DFs) greater than 50% were included in the analysis. As a control, we also investigated the absolute (i.e., not normalized) concentrations and came to similar results. The statistical analyses were performed with R/RStudio (Version 2024.09.0 Build 375, Posit Software, PBC).

Dust-air partition coefficients as $\log(K_{d}^{\prime })$ in log(m^3^/μg) were calculated for 6:2 FTOH, 8:2 FTOH, 10:2 FTOH, MeFOSE, and EtFOSE (all DF >50%) as described in Eichler et al. (2024a) as

(1) $$\log(K_{d}^{\prime })=\log\left(\frac{C_{d}}{C_{g}}\cdot \frac{10^{-6}g}{\mu g}\right),$$

using the PFAS concentrations in the indoor gas phase ($C_{g}$; ng/m^3^) reported in Eichler et al. (2023) and the PFAS concentrations in different dust size fractions ($C_{d}$; ng/g) measured in the 10 NC homes reported here.

## Results and discussion

### PFAS in field blanks

**Figure 1.**
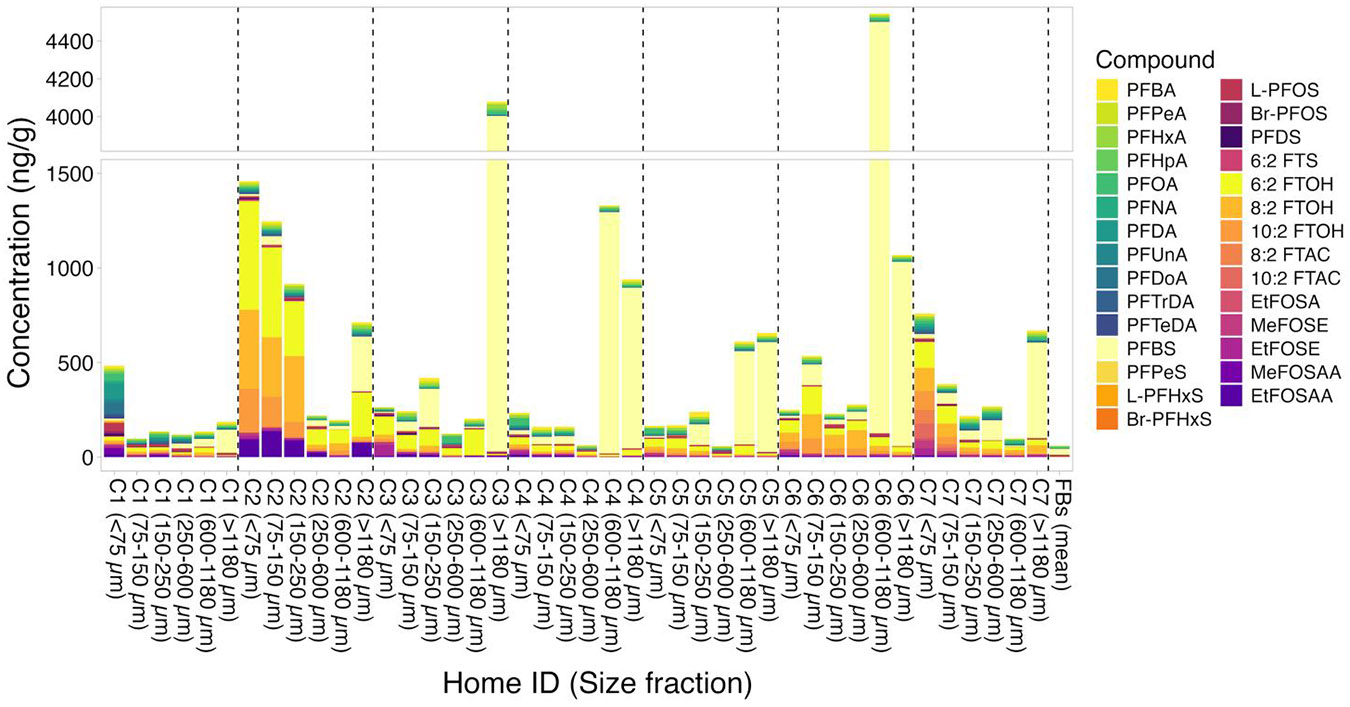
Concentrations of targeted PFAS detected at least once above MDL in dust samples from all NY homes (home IDs: C1-C7) and mean PFAS concentrations in the field blanks (FBs).

**Figure 2.**
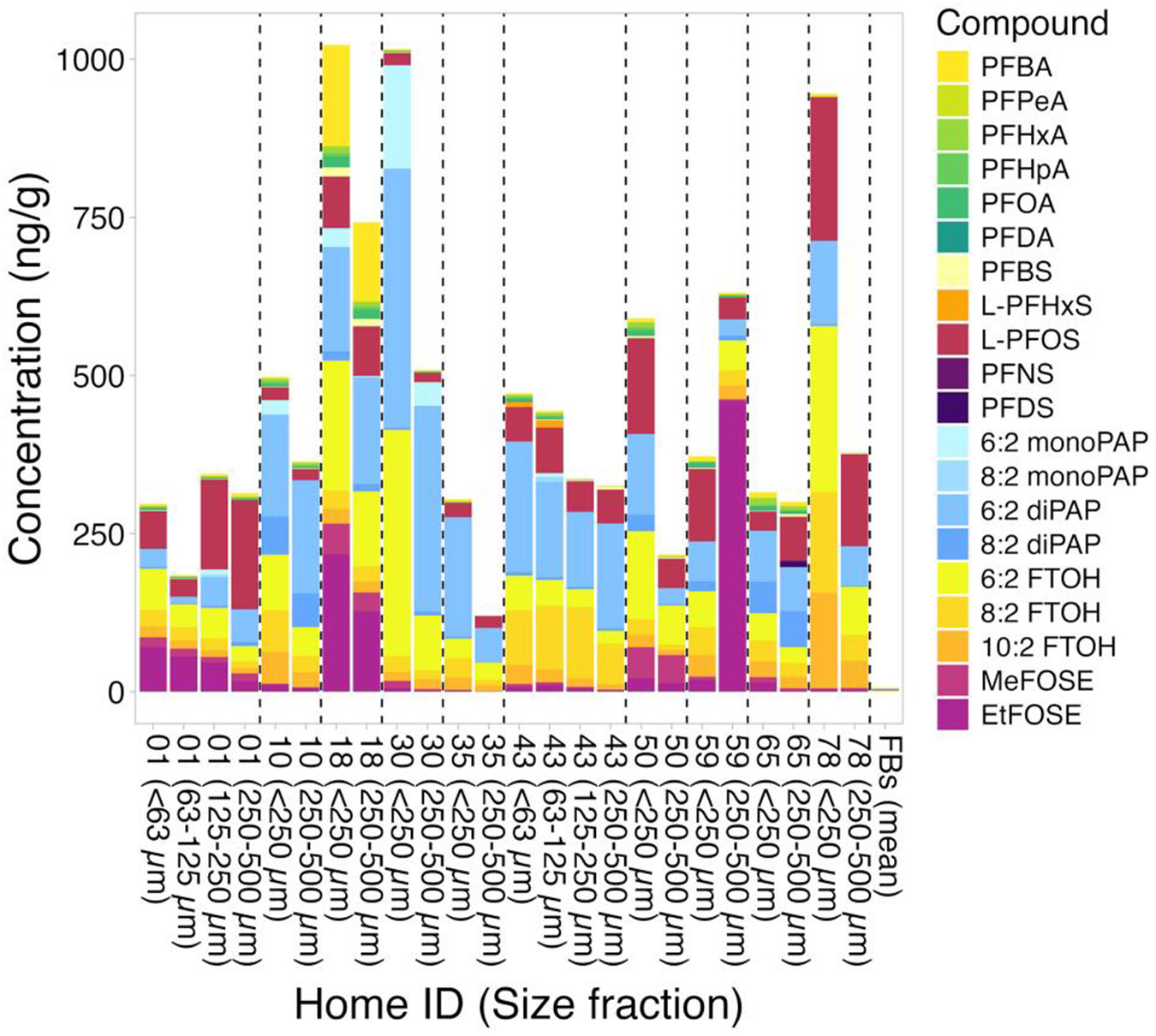
Concentrations of PFAS in dust samples from NC homes (home IDs: 01, 10, 18, 30, 35, 43, 50, 59, 65, and 78) detected at least once above MDL and mean PFAS concentrations in the field blanks (FBs).

Some PFAS were detected in the FBs collected from the NY and NC homes (Figures 1 and 2). Specifically, 6:2 and 8:2 FTOH were found in the FBs from NY homes C4, C5, and C6, however, all concentrations were below MDL. Of the ionic/ionizable PFAS, PFOA and L-PFOS were detected above MDL in all NY home FBs at mean concentrations comparable to mean PFOA and L-PFOS levels in the NY dust samples, indicating some potential for contamination. Further, PFBS was detected twice in the NY FBs (homes C1 and C5) at concentrations comparable to those in some dust samples but lower than others. In the FBs from the NC homes, 6:2 FTOH and EtFOSA were found at concentrations below MDL. PFBS, L-PFOS, and 8:2 diPAP were detected in all or most NC FBs. PFBS and 8:2 diPAP concentrations in the FBs were comparable to concentrations in the dust samples, while L-PFOS FB concentrations were about an order of magnitude lower than dust concentrations. PFPeA was detected only in the FB from home 18.

### PFAS and EOF in NY house dust samples

PFAS concentration profiles varied among the seven NY homes as well as among the different size fractions (Figure 1, Tables S6-S7). The number of detected PFAS was greatest in the smallest size fraction (<75 μm) for all homes. The C_4_-C_10_ PFCAs were present in all dust samples and size fractions, while the C_11_-C_14_ PFCAs were found in most NY dust samples, but less frequently in the larger size fractions. PFHxA, PFOA, and PFPeA were most frequently found, with detection frequencies (DFs) of 88%, 83%, and 62%, respectively. Of the PFSAs, PFBS (DF = 83%) and L-PFOS (DF = 67%) were most frequently detected. PFHpS, PFNS, and PFDoS were not detected in any samples. Of the emerging PFAS, only 6:2 FTS was detected above MDL in one NY dust sample (home C1, 150–250 μm fraction). Several other emerging PFAS (PFEESA, 7:3 FTCA, 5:3 FTCA, 8:2 FTS, 4:2 FTS, PFMPA (PF40PeA) and 9Cl-PF3ONS) were observed in some dust samples but always at levels below the MDL, and thus will not be further discussed. The FTOHs had DFs of 95% (6:2 FTOH), 74% (8:2 FTOH), and 64% (10:2 FTOH). The FOSEs were also detected frequently (DF = 88% for MeFOSE and 91% for EtFOSE). FTOHs and FOSEs were detected most frequently in the smaller size fractions (<250 μm) in the NY homes and the FTACs were present only in the two smallest size fractions from home C7. EtFOSA was detected above MDL only in the <75 μm fraction from home C7. MeFOSAA, EtFOSAA, and FOSA were found in several dust samples as well. However, their concentrations were mostly below MDL (DFs = 2.4%, 26%, and 0%, respectively).

Overall, observed concentrations were highest for PFBS. Samples from homes C3, C4, C5, C6 and C7 showed particularly high levels of PFBS of >500 ng/g in either the largest (>1,180 μm) or the second largest size fraction (600–1,180 μm). Concentrations of PFCAs, PFSAs, and emerging PFAS were generally one to two orders of magnitude lower than the PFBS concentrations, with slightly higher concentrations of PFCAs observed in the smaller size fractions (Figure S3). The <75 μm sample from home C1 had the highest levels of several PFCAs, including PFOA (36.7ng/g) and PFDA (83.8ng/g), compared to the other NY homes, as well as the highest concentration of PFOS (43.8 ng/g). In the home C2 dust samples, concentrations of 6:2 FTOH, 8:2 FTOH, and 10:2 FTOH were high compared to the other NY homes, with highest levels found in the size fractions smaller than 250 μm.

PFAS concentrations in dust reported in the literature vary greatly, and the levels of PFAS found in NY dust samples are generally consistent with the reported data for PFCAs, FTOHs, FOSEs, and most PFSAs (Eriksson and Kärman 2015; Fraser et al. 2013; Goosey and Harrad 2011; Hall et al. 2020; Huber et al. 2011; Kato et al. 2009; Shoeib et al. 2016; Strynar and Lindstrom 2008; Winkens et al. 2018; Wu et al. 2020; Yao et al. 2018; Young et al. 2021a). However, the PFBS concentrations found in homes C3 to C7 are on the higher end of the ranges found in other locations. One potential explanation is that most studies use dust sieved to size fractions of 500 μm or smaller, while we observed the highest PFBS levels in dust particles larger than 500 μm. Only two studies, in which the dust samples were either not sieved (Huber et al. 2011) or sieved to <2,000 μm (Kato et al. 2009), reported PFBS levels of up to 1089 ng/g and 7718 ng/g, respectively, which is more in line with the PFBS concentrations observed in homes C3 to C7. PFBS was also found in two of the seven NY field blanks at relatively high concentrations (home C1: 183 ng/g; home C5; 36.5 ng/g); thus, contamination with PFBS in the field or during sample processing may also be a potential explanation.

Concentrations of EOF in the NY house dust samples were all below the MDL of 20,000 ng/g; however, some information can still be gathered from the results. Homes C1 to C6 showed relatively high (>2,000ng/g) EOF concentrations in at least one of the investigated size fractions (Figure S4), with a tendency of higher EOF levels in the two smaller (<75 μm and 75–150 μm) size fractions. Overall, the range of EOF concentrations is comparable to the range of the sum of fluorine, ∑(F), levels measured in the targeted analysis of ionic/ionizable PFAS; however, ∑(F) exceeds EOF in several samples (Figure S4). In the future, the analysis of larger amounts of dust for both EOF and targeted PFAS could be beneficial, if the sample mass allows it, which was not the case here. The results further highlight the difficulties associated with using EOF as a proxy for PFAS concentrations in environmental samples.

### PFAS in NC house dust samples and comparison of neutral species

Given that the neutral species for NY and NC samples were analyzed by the same method in the same lab, we compare neutral species in the two regions directly below. Methodological differences across labs make us hesitant to make such a comparison for ionic PFAS (Figure S1). It also has to be noted that, in contrast to the NY homes, only homes 01 and 43 had dust samples sieved to more than two size fractions, and dust particles >500 μm were not included in the NC samples.

Figure 2 shows the results for all size fractions, for PFAS detected at least once above MDL in the NC homes. Occurrences and levels of PFAS detected in NC homes have been discussed in detail elsewhere, as weighted sums representing the <500 μm size fraction (Chang et al. 2025b; Eichler et al. 2024a). In brief, the C_4_-C_8_ PFCAs and PFDA (i.e., the C_10_ PFCA) were detected somewhat frequently, but not the C_9_ and C_11_-C_18_ PFCAs (Tables S8-S9). L-PFOS was detected most frequently (DF = 100%), followed by PFBS (DF = 71%), but other PFSAs (i.e., L-PFHxS, PFNS, and PFDS) were found above MDL in only very few samples. The diPAPs were observed in all dust samples (DFs = 100%), while the monoPAPs were found in only a few samples, with DFs (>MDL) of 29% and 8.3% for 6:2 monoPAP and 8:2 monoPAP, respectively. The FTOHs and FOSEs were detected frequently (DFs = 83%-100%), while the FTACs and FOSAs were not detected. Concentrations are generally comparable to those reported in the literature with highest levels observed for PFBA, L-PFOS, 6:2 diPAP, 6:2 monoPAP, the FTOHs, and the FOSEs (Chang et al. 2025b). PFCAs, FTOHs, and FOSEs tended to be found at higher concentrations in the smaller size fractions (with exceptions), while the PFSAs were present at comparable levels in all size fractions and in some cases even showed higher concentrations in the larger size fractions. No clear trend was observed for the PAPs (Figure S5).

In both sampling locations, FTOHs and FOSEs were detected frequently. Mean FTOH concentrations in the <600 μm (NY homes) and <500 μm (NC) size fractions are comparable (Figure S6), with highest levels observed for 6:2 FTOH (NY: 70 ng/g; NC: 107 ng/g), followed by 8:2 FTOH (NY: 55 ng/g; NC: 45 ng/g) and 10:2 FTOH (NY: 24ng/g; NC: 31 ng/g). MeFOSE (mean: NY: 6.4ng/g; NC: 12ng/g) and EtFOSE (mean: NY: 7.6 ng/g; NC: 47 ng/g) concentrations vary more, however, the effect of the sampling location on the concentrations was not statistically significant (*p* > 0.05).

### Correlations between size fractions and normalized PFAS concentrations and insights about PFAS sources

Based on detection frequencies and measured concentrations, we focus on the following PFAS in this section: PFBA, PFHxA, PFHpA, PFOA, PFDA, PFBS, L-PFHxS, L-PFOS, 6:2 FTOH, 8:2 FTOH, 10:2 FTOH, MeFOSE, and EtFOSE as well as for the sum of the C_4_ to C_14_ PFCAs (∑(PFCAs)), the sum of the C_4_ to C_10_ PFSAs excluding Br-PFHxS and Br-PFOS (∑(PFSAs)), the sum of the three FTOHs, two FTACs, EtFOSA, MeFOSE, and EtFOSE (∑(neutralPFAS)), and the sum of all PFAS (∑(PFAS)) included in the analysis of the dust samples from the two locations. Correlations for additional PFAS are shown for homes C1 to C7 in Figures S7–S13 and for homes 01 and 43 in Figures S14–S15, however, these will not be further discussed.

**Figure 3.**
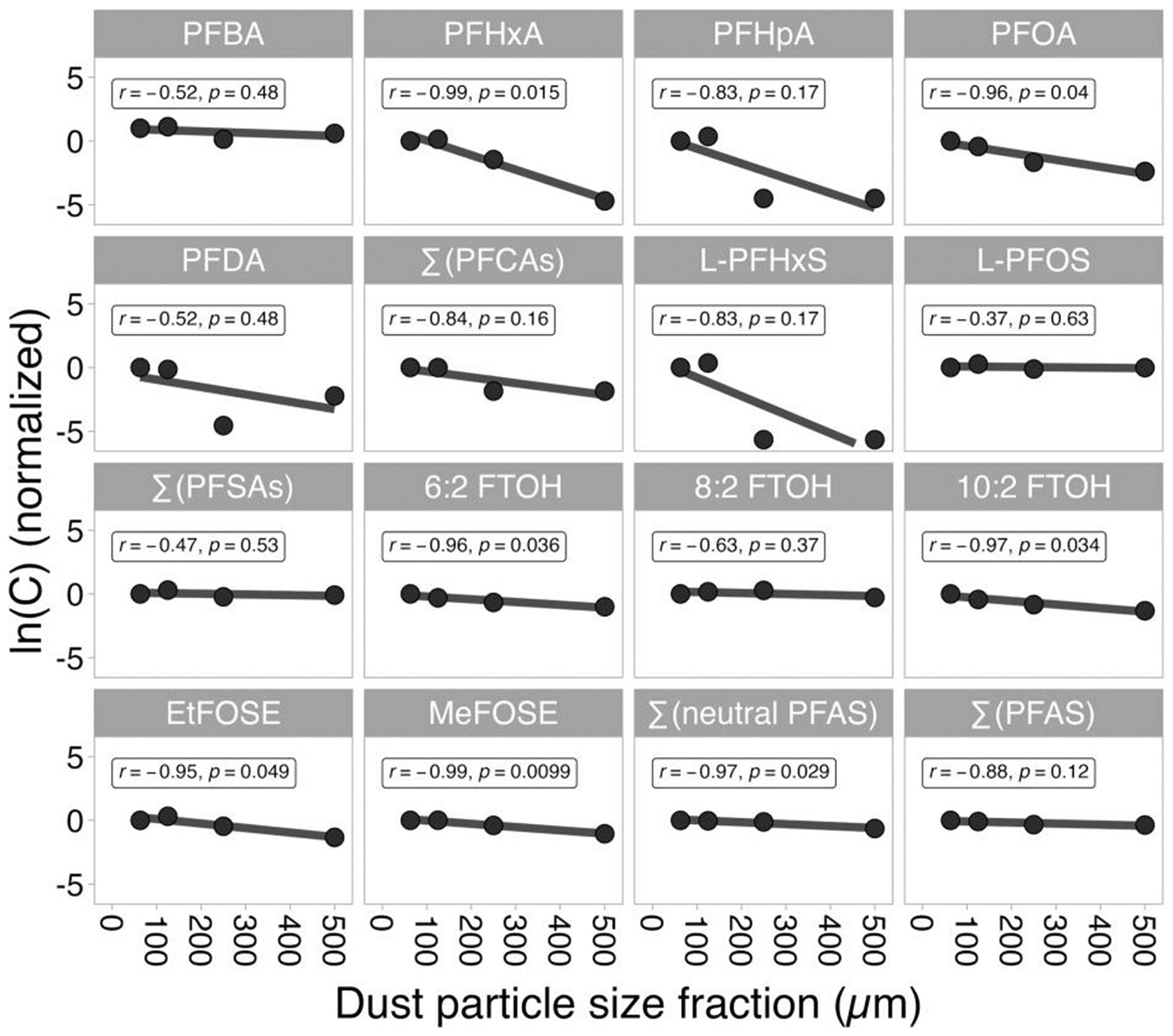
Correlations between normalized, ln-transformed PFAS concentrations and dust particle size fractions for home 43 (North Carolina). Concentrations were normalized by the concentrations measured in the 63 μm size fraction.

The relationship between normalized PFAS concentrations and the size fraction shows varying patterns across homes for PFCAs and PFSAs but similarities across all homes for neutral PFAS. For example, all normalized PFCA concentrations in home 43 dust showed strong, although not significant (*p* > 0.05), negative correlations with particle size (Figure 3), while dust samples from other homes showed varying trends for the same compounds (Figure S7-S15; Figure S16a). Further, samples from all NY homes indicate a strong positive correlation between normalized PFBS concentrations and particle size (Figure S7A-S13A), while home 01 shows a negative correlation (Figure S14). Varying trends can also be observed for L-PFOS, with strong to moderately strong negative correlations observed for homes C7 and 43 and a strong positive correlation found for home 01. In contrast, correlations of 6:2 FTOH, 8:2 FTOH, 10:2 FTOH, MeFOSE, and EtFOSE with particle size are strong to moderately strong and negative for all homes, and in several cases also statistically significant.

Considering only a narrower, more comparable range of size fractions, i.e., <75–600 μm for NY homes (Figure S7B-S13B; Figure S16b) and <63–500 μm for NC homes, we see a shift toward more negative correlations for several PFAS that showed weak or more positive correlations if larger size fractions were included, such as PFBA, PFDA, and PFBS. This indicates that for these compounds, the >600 μm size fractions accumulate PFAS based on a different mechanism.

**Figure 4.**
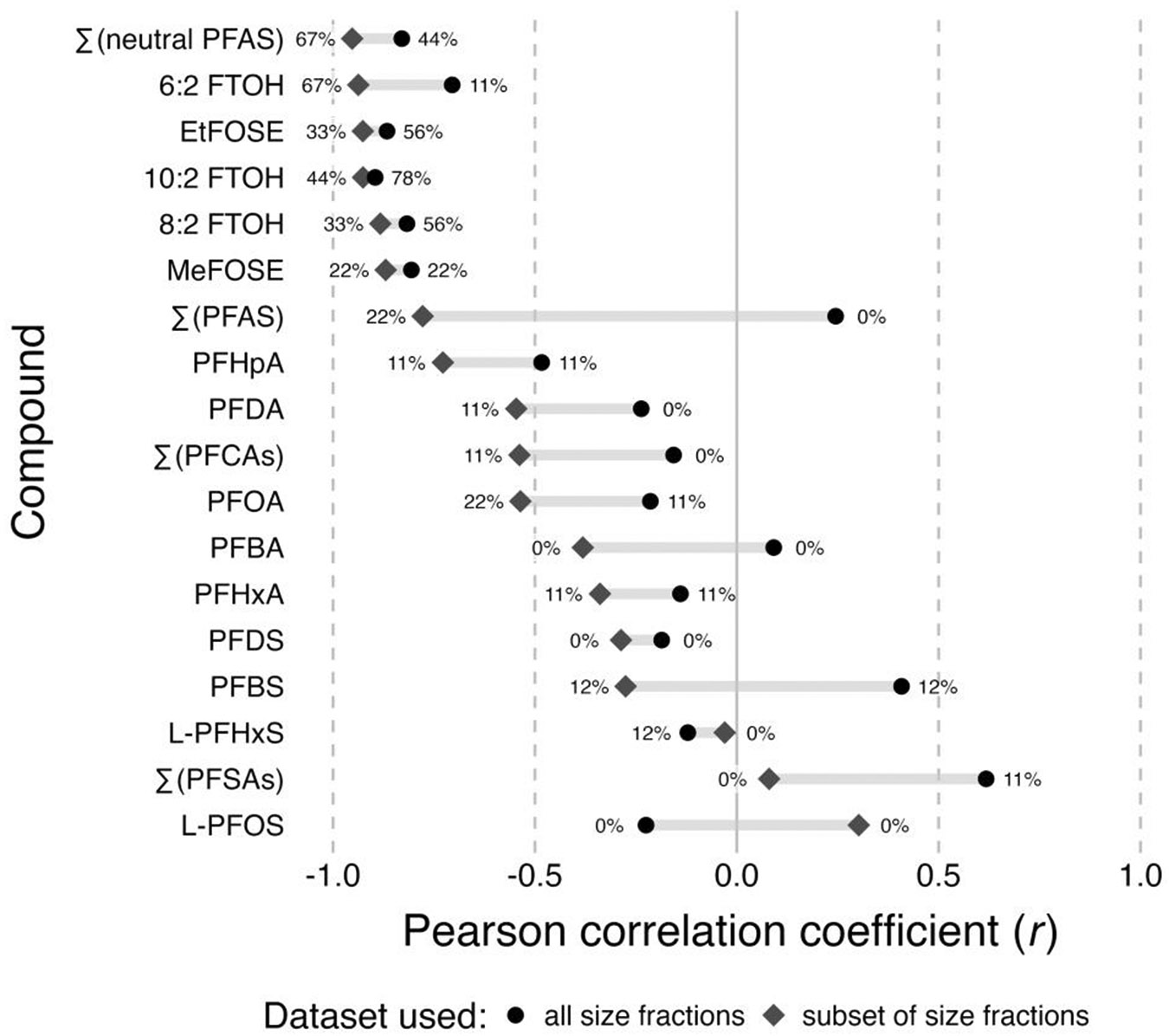
Mean Pearson correlation coefficients (r) for PFAS concentrations in dust and the dust particle size fraction for the full dataset (black dots; NC homes: <63 to 250–500 μm size fractions, NY homes: <75 to 1,180-2,000 μm size fractions) and the subset of size fractions (gray diamonds; NC homes: <63 to 250–500 μm size fractions, NY homes: <75 to 250–600 μm size fractions) sorted from most negative to most positive mean r based on the subset. The percentages indicate for what portion of homes the correlation was significant.

Strong negative associations hint at partitioning from the gas phase or directly from the source material to the dust particles as the main migration pathway. Because smaller particles provide a larger surface area per unit mass, concentrations are expected to be higher in the smaller size fraction if partitioning preferentially to the dust particle surface is the main mechanism. Particles and fibers abraded from a source and accumulated in dust are likely larger (Cao et al. 2014; Webster et al. 2009). The presence of these particles and fibers, including microplastics that are often coated with chemical additives (Dris et al. 2017; Rochman et al. 2019), results in positive correlations, such as those observed for PFBS. Thus, several of the NY homes may have sources of PFBS-containing particles, e.g., a carpet or upholstery, leading to high concentrations of PFBS in the larger (here, >600 μm) size fraction (Figures S7-S13). In home 01, a source of L-PFOS particles or fibers may be present, as indicated by higher concentrations in the 250–500 μm size fraction (r=0.78, Figure S14). However, if only the smaller size fractions are included in the analysis, a general tendency of all considered PFAS to accumulate in dust because of partitioning becomes evident, as indicated by a mean r of ∑(PFAS) of −0.78 and a shift of r to the left, i.e., closer to −1 (Figure 4; Figure S16), compared to the results when all size fractions are included, where the mean r of ∑(PFAS) is 0.24. Overall, the results of the correlation analyses were most consistent for neutral PFAS, which further strengthens the hypothesis that migration of FTOHs and FOSEs into dust is driven by partitioning. The more mixed results for PFCAs and particularly PFSAs indicates that for those compounds, multiple migration pathways have to be considered.

Our findings are consistent with those reported in the study by Gustafsson et al. who measured concentrations of several PFCAs, PFOS, and PAPs in different size fractions of a composite dust sample (Gustafsson et al. 2022). Although correlations are not provided, their data indicate similar trends as those observed in our work, with generally higher concentrations in smaller size fractions, as well as the presence of some very high concentrations in the large size fraction, especially for the PFCAs.

### The effect of particle size on FTOH and FOSE dust-air partition coefficients

**Figure 5.**
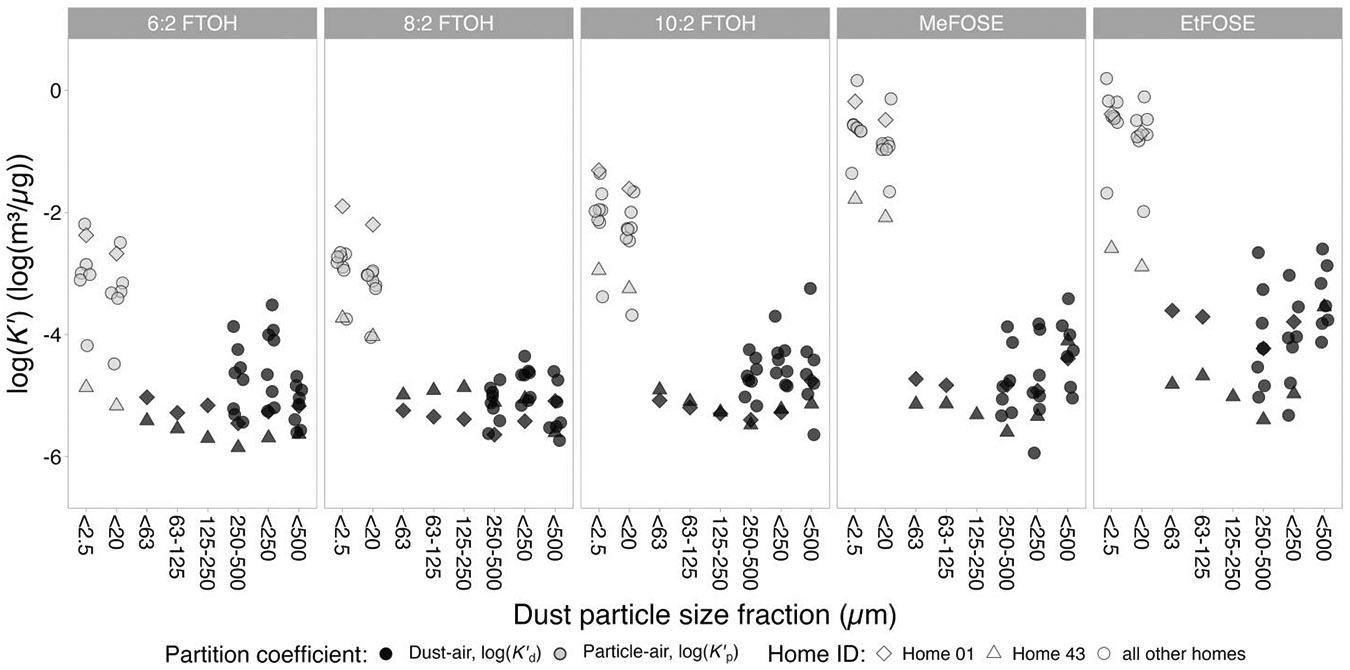
Dust-air partition coefficients ($\log(K_{d}^{\prime })$; dark gray markers) and particle-air partition coefficients ($\log(K_{p}^{\prime })$; light gray markers) for different dust particle size fractions and homes. $\log(K_{p}^{\prime })$ values for the <2.5 μm (i.e., PM_2.5_) and <20 μm size fractions were previously reported in Eichler et al. (2024a). $\log(K_{d}^{\prime })$ values for the 125–250 μm, 63–125 μm, and <63 μm size fractions could only be derived for Home 01 (diamond) and Home 43 (triangle).

We observed higher $\log(K_{d}^{\prime })$ values for smaller dust size fractions compared to larger size fractions (Figure 5, Table S10). For NC homes 01 and 43, for which $\log(K_{d}^{\prime })$ could be derived for multiple size fractions, the correlation coefficients ranged from −0.57 to −1, and several correlations were significant (Figure S17). This finding is consistent with the even higher particle-air partition coefficients ($\log(K_{d}^{\prime })$) previously measured (Eichler et al. 2024a) for airborne particles of <2.5 μm and <20 μm particle size (Figure 5). Our results also show that $\log(K_{d}^{\prime })$ values based on PFAS concentrations in weighted sums (i.e., <250 μm and <500 μm size fractions) provide averaged values for $\log(K_{d}^{\prime })$ and seem to be strongly influenced by smaller size fractions (i.e., these $\log(K_{d}^{\prime })$ values are relatively high). Thus, if $\log(K_{d}^{\prime })$ is used to estimate concentrations in dust from concentrations in air as part of exposure assessments, $\log(K_{d}^{\prime })$ should be chosen carefully to match the dust size fraction considered in the assessment. When comparing $\log(K_{d}^{\prime })$ for the smallest available size fractions (63–125 μm and <63 μm) to $\log(K_{d}^{\prime })$ previously derived for airborne particles (Eichler et al. 2024a), specifically for total suspended particles (TSP, <20 μm) and particulate matter with a diameter of less than 2.5 μm (PM_2.5_), a continuation of the general trend of increasing partition coefficients with decreasing particle size fractions can be observed (Figure 5). However, this continuation is not entirely linear, especially not for MeFOSE and EtFOSE. A possible explanation may be the difference in composition of airborne particles compared to dust particles, which influences particle-air partitioning (Hodas et al. 2012; Hodas and Turpin 2014). On average, ~50% of indoor fine particulate matter originates outdoors, while the remaining ~50% is generated indoors, including *via* resuspension of dust particles (Long et al. 2000; Meng et al. 2005; Qian et al. 2008). Thus, organic gas-phase chemicals, including PFAS, likely have a different affinity to airborne particles than to dust particles.

### Health implications and the role of dust particle size in exposure assessments

Of the PFAS detected most frequently across all dust samples from both locations, i.e., PFPeA, PFOA, L-PFOS, 6:2 FTOH, 8:2 FTOH, 10:2 FTOH, MeFOSE, and EtFOSE, most information on their toxicity in humans is available for the legacy PFAS PFOA and L-PFOS (Fenton et al. 2021; Rappazzo et al. 2017). Both have been linked to immunotoxicity, liver disease and liver cancer, kidney disease and kidney cancer, lipid and insulin dysregulation, and negative reproductive and developmental outcomes (Fenton et al. 2021; Rappazzo et al. 2017; Sunderland et al. 2019). PFOA has also been associated with thyroid disease, testicular cancer, and childhood obesity (Fenton et al. 2021; Sunderland et al. 2019). Not much toxicity information is available on PFPeA; however, some evidence suggests that PFAS with less than six fluorinated carbons, which includes PFPeA, have lower toxicity than long-chain PFAS (Fenton et al. 2021; Sunderland et al. 2019). Additional research is necessary to substantiate these observations. Toxicity data on neutral PFAS, including FTOHs and FOSEs, are limited. Chronic exposure to EtFOSE has been linked to carcinogenicity in rats and reductions in total cholesterol, increased liver weight, and immunotoxicity in rhesus monkeys (Sunderland et al. 2019). FTOHs can be metabolized in the human body to form PFCAs of the same chain length as the FTOH or of shorter chain length, thus becoming a source of exposure to PFOA and other PFCAs (Butt et al. 2014; Rice et al. 2020). Rice et al. (2020) further concluded that 6:2 FTOH is more toxic to humans than PFHxA due to the formation of other metabolites with known or suspected toxicity, e.g., 5:3 acid. Again, additional research is needed to close the knowledge gaps associated with neutral PFAS toxicity.

Our work highlights that the choice of dust particle size fraction matters when measurements and exposure assessments are conducted. Higher concentrations of many PFAS, including FTOHs, FOSEs, and several PFCAs, in smaller size fractions indicate that it may be reasonable to focus on size fractions of <250 μm when assessing exposure. Smaller size fractions may be particularly relevant when hand-to-mouth transfer and transdermal uptake are of interest, as discussed above. Further, the possibility of dust resuspension from walking or other human activity makes exposure *via* inhalation of small particles more likely. Smaller particles can be inhaled more deeply, and if these particles are also associated with higher levels of PFAS, then they warrant particular attention. However, this research further shows that larger dust size fractions can be significant sources of exposure to PFAS as well. Dust particles larger than 600 μm may be less likely to be inhaled or ingested, however, these particles also constitute a potential risk because they can represent a source of PFAS to the indoor environment, prolonging the presence of PFAS in the home. They may further be indicators of specific types of PFAS sources, e.g., carpets and upholstery, which makes them of interest if source apportionment is the goal. Discarding larger size fractions entirely may therefore not give a complete picture of the potential for PFAS exposure in a home. The choice of the dust size fraction to use in exposure assessments thus depends strongly on the most likely pathway of exposure, and multiple size fractions should be considered if several pathways are expected to contribute.

The appropriate choice of size fraction may also depend on the individual or group of PFAS. The greater variability of PFSA concentrations in different size fractions may warrant measurements of these PFAS in a broader range of particles sizes than may be required for FTOHs or FOSEs. The variability of correlations observed among homes adds another layer of complexity, because different size fractions may be relevant for different homes. Together, these considerations pose additional challenges for PFAS exposure assessments and emphasize the need for clearly defined hypotheses.

### Limitations

This study has several limitations that have to be considered. For our analysis, we used a relatively small dataset with measurements from only two different locations in the US. While the results indicate trends that can be found across different homes and geographic locations, additional research at a larger number and in different types of homes would help to further evaluate our findings. Controlled chamber experiments may also provide important insights into the underlying mechanisms that result in the observed differences in concentration profiles by particle size. Also, the size fractions considered were not exactly the same in the two locations, and only two of the NC homes had enough dust to sieve it to more than two size fractions. Differences in collection times and environmental conditions also posed a limitation to the study, potentially influencing the consistency of the results. The use of two different analytical methods, including slightly different target analytes, made a direct comparison of the results for the ionic/ionizable PFAS difficult and required normalizing the data for further analysis. Only limited data were available to determine dust-air partition coefficients, because air sampling was not part of the NY study. However, the relevance of the dust particle size fraction was evident for both datasets despite these limitations and emphasizes the need for careful consideration of size fractionation in field campaigns and exposure assessments.

## Supplementary Material

Supp 1

Supplemental data for this article can be accessed online at https://doi.org/10.1080/02786826.2025.2582532.
