# Supplementary material for: The impact of particle size on PFAS concentrations in dust from homes in North Carolina and New York and implications for exposure
Source: Aerosol Sci Technol. Author manuscript; Available in PMC 2025 Dec 25. (PMC12721804; doi:10.1080/02786826.2025.2582532)
Supplement: Supp 1 [file NIHMS2123801-supplement-Supp_1.docx]

Supplemental Information

**The impact of particle size on PFAS concentrations in dust from homes in North Carolina and New York and implications for exposure**

Clara M. A. Eichler^a^, Mahender Singh Rawat^b^, Naomi Y. Chang^a^, Elizabeth Brown^b^, Sujan Fernando^c^, Thomas M. Holsen^b,c^, Glenn C. Morrison^a^, Andrea R. Ferro^b,c^, and Barbara J. Turpin^a^

^a^Department of Environmental Sciences and Engineering, The University of North Carolina at Chapel Hill, Chapel Hill, NC, USA

^b^Department of Civil and Environmental Engineering, Clarkson University, Potsdam, NY, USA

^c^Center for Air and Aquatic Resources Engineering and Science, Clarkson University, Potsdam, NY, USA

CONTACT

Clara M. A. Eichler [claramae@unc.edu](mailto:claramae@unc.edu) Department of Environmental Sciences and Engineering, The University of North Carolina at Chapel Hill, 166C Rosenau Hall, CB#7431, Chapel Hill, NC 27599, USA

Andrea R. Ferro [aferro@clarkson.edu](mailto:aferro@clarkson.edu) Department of Civil and Environmental Engineering, Clarkson University, 206 Rowley Laboratories, Box 5710, Potsdam, NY 13699, USA

**No. of pages: 39**

**No. of figures: 17**

**No. of tables: 10**

**Table of Content**

| **Table S1**: Native and mass-labeled analytes included in targeted analyses | S4 |
| --- | --- |
| **Section S1: Extraction and Analysis for Neutral PFAS** | S7 |
| **Table S2**: Extraction efficiencies (recoveries) for the New York samples | S8 |
| **Table S3**: Method detection limits (MDLs) for neutral PFAS in dust analyzed by GC-MS as described in Eichler et al. (2024) | S8 |
| **Section S2: Extraction and Analysis for Ionic/Ionizable PFAS at UNC-CH** | S9 |
| **Table S4**: Method detection limits (MDLs) of the UNC-CH method used here for ionic/ionizable PFAS (LC-MS/MS). See also Chang et al. (2025) | S9 |
| **Section S3: Extraction and Analysis for Ionic/Ionizable PFAS at Clarkson** | S10 |
| **Table S5**: Method detection limits (MDLs) of the Clarkson method (LC-MS/MS). MDLs were established as sample-spiked MDLs as described in EPA Method 1633A (USEPA 2024) and in USEPA (2016) | S11 |
| **Section S4: Comparison of the Results from the UNC-CH Method and Clarkson Method** | S11 |
| **Figure S1**: Results for the same samples analyzed by the Clarkson method and the UNC-CH method | S12 |
| **Section S5: Extractable Organic Fluorine (EOF) Analysis** | S13 |
| **Section S6: Calculation of Weighted Sums** | S14 |
| **Figure S2**: Histograms showing the distributions of the ln-transformed, normalized concentrations of targeted PFAS across all dust samples. Non-detects were replaced by MDL/2 prior to ln-transformation | S15 |
| **Table S6**: PFAS measured in dust samples and field blanks (FBs) from New York (NY) homes using the Clarkson method for ionic/ionizable PFAS (LC-MS/MS) | S16 |
| **Table S7**: Neutral PFAS measured in dust samples from New York (NY) homes | S19 |
| **Figure S3**: Box-and-whisker plots of ∑(PFCAs), ∑(PFSAs), ∑(emerging PFAS), ∑(neutral PFAS) and ∑(PFAS) found in six different size fractions in NY homes | S21 |
| **Figure S4**: Extractable organic fluorine (EOF, green) and sum of measured ionic/ionizable PFAS as fluorine (∑(F); orange) concentrations in NY house dust samples (home IDs C1-C7). Note that all EOF values shown are below the MDL of 20,000 ng/g | S22 |
| **Table S8**: PFAS measured in dust samples and field blanks (FBs) from North Carolina (NC) homes using the UNC-CH method for ionic/ionizable PFAS (LC-MS/MS). Calculated concentrations in the <500 µm size fractions and field blank concentrations have been reported in Chang et al. (2025) | S23 |
| **Table S9**: Neutral PFAS measured in dust samples and field blanks (FBs) from North Carolina (NC) homes. Calculated concentrations in the <500 µm fractions have been reported in Eichler et al. (2024) | S25 |
| **Figure S5**: Box-and-whisker plots of ∑(PFCAs), ∑(PFSAs), ∑(PAPs), ∑(neutral PFAS) and ∑(PFAS) found in four different size fractions in NC homes | S26 |
| **Figure S6**: Concentrations of neutral PFAS measured with >50% DF above MDL in North Carolina (homes 01-78) and New York (homes C1-C7), weighted sums | S27 |
| **Figure S7**: Correlations between normalized PFAS concentrations and size fraction found for Home C1 (New York). Concentrations were normalized by the concentrations measured in the 75 µm size fractions. A) Full range of size fractions (<63 µm to <2,000 µm); B) subset of size fractions (<63 µm to <600 µm) | S28 |
| **Figure S8**: Correlations between normalized PFAS concentrations and size fraction found for Home C2 (New York). Concentrations were normalized by the concentrations measured in the 75 µm size fractions. A) Full range of size fractions (<63 µm to <2,000 µm); B) subset of size fractions (<63 µm to <600 µm) | S29 |
| **Figure S9**: Correlations between normalized PFAS concentrations and size fraction found for Home C3 (New York). Concentrations were normalized by the concentrations measured in the 75 µm size fractions. PFHpS was not detected in enough size fractions from Home C3. A) Full range of size fractions (<63 µm to <2,000 µm); B) subset of size fractions (<63 µm to <600 µm) | S30 |
| **Figure S10**: Correlations between normalized PFAS concentrations and size fraction found for Home C4 (New York). Concentrations were normalized by the concentrations measured in the 75 µm size fractions. PFHpS was not detected in dust samples from Home C4. A) Full range of size fractions (<63 µm to <2,000 µm); B) subset of size fractions (<63 µm to <600 µm) | S31 |
| **Figure S11**: Correlations between normalized PFAS concentrations and size fraction found for Home C5 (New York). Concentrations were normalized by the concentrations measured in the 75 µm size fractions. PFHpS, PFDS, and FOSA were not detected in Home C5. A) Full range of size fractions (<63 µm to <2,000 µm); B) subset of size fractions (<63 µm to <600 µm) | S32 |
| **Figure S12**: Correlations between normalized PFAS concentrations and size fraction found for Home C6 (New York). Concentrations were normalized by the concentrations measured in the 75 µm size fractions. A) Full range of size fractions (<63 µm to <2,000 µm); B) subset of size fractions (<63 µm to <600 µm) | S33 |
| **Figure S13**: Correlations between normalized PFAS concentrations and size fraction found for Home C7 (New York). Concentrations were normalized by the concentrations measured in the 75 µm size fractions. PFHpS was not detected in enough size fractions from Home C7. A) Full range of size fractions (<63 µm to <2,000 µm); B) subset of size fractions (<63 µm to <600 µm) | S34 |
| **Figure S14**: Correlations between normalized PFAS concentrations and size fraction found for Home 01 (North Carolina). Concentrations were normalized by the concentrations measured in the 63 µm size fractions | S35 |
| **Figure S15**: Correlations between normalized PFAS concentrations and size fraction found for Home 43 (North Carolina). Concentrations were normalized by the concentrations measured in the 63 µm size fractions | S35 |
| **Figure S16**: Correlation matrices summarizing the Pearson correlation coefficients (*r*) for the NC homes as shown in **Fig. S14-S15** and A) the full range of size fractions (<63 µm to <2,000 µm) for the NY homes as shown in **Fig. S7A-S13A** and B) the subset of size fractions (<63 µm to <600 µm) for the NY homes as shown in **Fig. S7B-S13B**. Mean *r* values were calculated based on the Fisher-transformed *r* values from all homes (Corey, Dunlap and Burke 1998). The asterisk (*) indicates a significant correlation (p < 0.05). “NA” = not available. | S36 |
| **Table S10**: Dust-air partition coefficients as ${\log(K'}_{d})$ of 6:2 FTOH, 8:2 FTOH, 10:2 FTOH, MeFOSE, and EtFOSE measured in NC homes for different size fractions | S37 |
| **Figure S17**: Correlations between the dust-air partition coefficient, ${\log(K'}_{d})$, and the dust size fraction for 6:2 FTOH, 8:2 FTOH, 10:2 FTOH, MeFOSE, and EtFOSE, based on data from North Carolina homes 01 and 43 | S38 |
| **References** | S39 |

**Table S1**: Native and mass-labeled analytes included in targeted analyses

| **PFAS**  **Acronym** | **PFAS** | **CAS#** | **Formula** | **Mass-Labelled PFAS Standard** |
| --- | --- | --- | --- | --- |
| **Ionic/ionizable PFAS – Analyzed by UHPLC-ESI-MS/MS (UNC-CH Method)** | | | | |
| PFBA | Perfluoro-n-butanoic acid | 375-22-4 | C_4_HF_7_O_2_ | ^13^C_4_-PFBA |
| PFPeA | Perfluoro-n-pentanoic acid | 2706-90-3 | C_5_HF_9_O_2_ | ^13^C_5_-PFPeA |
| PFHxA | Perfluoro-n-hexanoic acid | 307-24-4 | C_6_HF_11_O_2_ | ^13^C_5_PFHxA |
| PFHpA | Perfluoro-n-heptanoic acid | 375-85-9 | C_7_HF_13_O_2_ | ^13^C_4_-PFHpA |
| PFOA | Perfluoro-n-octanoic acid | 335-67-1 | C_8_HF_15_O2 | ^13^C_8_-PFOA |
| PFNA | Perfluoro-n-nonanoic acid | 375-95-1 | C_9_HF_17_O_2_ | ^13^C_9_-PFNA |
| PFDA | Perfluoro-n-decanoic acid | 335-76-2 | C_10_HF_19_O_2_ | ^13^C_6_-PFDA |
| PFUnA | Perfluoro-n-undecanoic acid | 2058-94-8 | C_11_HF_21_O_2_ | ^13^C_7_-PFUnA |
| PFDoA | Perfluoro-n-dodecanoic acid | 307-55-1 | C_12_HF_23_O_2_ | ^13^C_2_-PFDoA |
| PFTrDA | Perfluoro-n-tridecanoic acid | 72629-94-8 | C_13_HF_25_O_2_ | ^13^C_2_-PFDoA |
| PFTeDA | Perfluoro-n-tetradecanoic acid | 376-06-7 | C_14_HF_27_O_2_ | ^13^C_2_-PFTeDA |
| PFHxDA | Perfluoro-n-hexadecanoic acid | 67905-19-5 | C_16_HF_31_O_2_ | ^13^C_2_-PFTeDA |
| PFODA | Perfluoro-n-octadecanoic acid | 16517-11-6 | C_18_HF_35_O_2_ | ^13^C_2_-PFTeDA |
| PFBS | Perfluorobutane sulfonic acid | 375-73-5 | C_4_HF_9_O_3_S | ^13^C_3_-PFBS |
| PFPeS | Perfluoropentane sulfonic acid | 2706-91-4 | C_5_HF_11_O_3_S | ^13^C_3_-PFBS |
| L-PFHxS | Perfluorohexane sulfonic acid | 355-46-4 | C_6_HF_13_O_3_S | ^13^C_3_-PFHxS |
| PFHpS | Perfluoroheptane sulfonic acid | 375-92-8 | C_7_HF_15_O_3_S | ^13^C_3_-PFHxS |
| L-PFOS | Perfluorooctane sulfonic acid | 1763-23-1 | C_8_HF_17_O_3_S | ^13^C_8_-PFOS |
| PFNS | Perfluorononane sulfonic acid | 68259-12-1 | C_9_HF_19_O_3_S | ^13^C_8_-PFOS |
| PFDS | Perfluorodecane sulfonic acid | 335-77-3 | C_10_HF_21_O_3_S | ^13^C_8_-PFOS |
| PFDoS | Perfluorododecane sulfonic acid | 79780-39-5 | C_12_HF_25_O_3_S | ^13^C_8_-PFOS |
| GenX | Hexafluoropropylene oxide-dimer acid (HFPO-DA) | 13252-13-6 | C_6_HF_11_O_3_ | ^13^C_3_-PFPrOPrA |
| 6:2 monoPAP | Sodium 1H,1H,2H,2H-perfluorooctyl phosphate | 57678-01-0 | C_8_H_4_F_13_O_4_PNa_2_ | ^13^C_2_-^12^C_6_H_4_F_17_O_4_PNa_2_ |
| 8:2 monoPAP | Sodium 1H,1H,2H,2H-perfluorodecyl phosphate | 57678-03-2 | C_10_H_4_F_17_O_4_PNa_2_ | ^13^C_2_-^12^C_8_H_4_F_17_O_4_PNa_2_ |
| 6:2 diPAP | Sodium bis(1H,1H,2H,2H-perfluorooctyl) phosphate | 57677-95-9 | C_16_H_8_F_26_O_4_PNa | ^13^C_4_-^12^C_12_H_8_F_26_O_4_PNa |
| 8:2 diPAP | Sodium bis(1H,1H,2H,2H-perfluorodecyl) phosphate | 114519-85-6 | C_20_H_8_F_34_O_4_PNa | ^13^C_4_-^12^C_16_H_8_F_34_O_4_PNa |
| **Ionic/ionizable PFAS – Analyzed by LC-MS/MS (Clarkson Method)** | | | | |
| PFBA | Perfluoro-n-butanoic acid | 375-22-4 | C_4_HF_7_O_2_ | ^13^C_4_-PFBA |
| PFPeA | Perfluoro-n-pentanoic acid | 2706-90-3 | C_5_HF_9_O_2_ | ^13^C_5_-PFPeA |
| PFHxA | Perfluoro-n-hexanoic acid | 307-24-4 | C_6_HF_11_O_2_ | ^13^C_5_PFHxA |
| PFHpA | Perfluoro-n-heptanoic acid | 375-85-9 | C_7_HF_13_O_2_ | ^13^C_4_-PFHpA |
| PFOA | Perfluoro-n-octanoic acid | 335-67-1 | C_8_HF_15_O2 | ^13^C_8_-PFOA |
| PFNA | Perfluoro-n-nonanoic acid | 375-95-1 | C_9_HF_17_O_2_ | ^13^C_9_-PFNA |
| PFDA | Perfluoro-n-decanoic acid | 335-76-2 | C_10_HF_19_O_2_ | ^13^C_6_-PFDA |
| PFuDA | Perfluoro-n-undecanoic acid | 2058-94-8 | C_11_HF_21_O_2_ | ^13^C_7_-PFUnA |
| PFDoA | Perfluoro-n-dodecanoic acid | 307-55-1 | C_12_HF_23_O_2_ | ^13^C_2_-PFDoA |
| PFTrDA | Perfluoro-n-tridecanoic acid | 72629-94-8 | C_13_HF_25_O_2_ | ^13^C_2_-PFDoA |
| PFTeDA | Perfluoro-n-tetradecanoic acid | 376-06-7 | C_14_HF_27_O_2_ | ^13^C_2_-PFTeDA |
| PFBS | Perfluorobutane sulfonic acid | 375-73-5 | C_4_HF_9_O_3_S | ^13^C_3_-PFBS |
| PFPeS | Perfluoropentane sulfonic acid | 2706-91-4 | C_5_HF_11_O_3_S | ^13^C_3_-PFHxS |
| L-PFHxS | Linear perfluorohexane sulfonic acid | 355-46-4 | C_6_HF_13_O_3_S | ^13^C_3_-PFHxS |
| Br-PFHxS | Branched perfluorohexane sulfonic acid | 355-46-4 | C_6_HF_13_O_3_S | ^13^C_3_-PFHxS |
| PFHpS | Perfluoroheptane sulfonic acid | 375-92-8 | C_7_HF_15_O_3_S | ^13^C_8_-PFOS |
| L-PFOS | Linear perfluorooctane sulfonic acid | 1763-23-1 | C_8_HF_17_O_3_S | ^13^C_8_-PFOS |
| Br-[PFOS](https://pubchem.ncbi.nlm.nih.gov/#query=C4HF7O3) | Branched perfluorooctane sulfonic acid | 1763-23-1 | C_8_HF_17_O_3_S | ^13^C_8_-PFOS |
| PFNS | Perfluorononane sulfonic acid | 68259-12-1 | C_9_HF_19_O_3_S | ^13^C_8_-PFOS |
| PFDS | Perfluorodecane sulfonic acid | 335-77-3 | C_10_HF_21_O_3_S | ^13^C_8_-PFOS |
| PFDoS | Perfluorododecane sulfonic acid | 79780-39-5 | C_12_HF_25_O_3_S | ^13^C_8_-PFOS |
| GenX | Hexafluoropropylene oxide-dimer acid (HFPO-DA) | 13252-13-6 | C_6_HF_11_O_3_ | ^13^C_3_-HFPO-DA |
| NaDONA | 4,8-Dioxa-3H-perfluorononanoic acid | 919005-14-4 | C_7_H_2_F_12_O_4_ | ^13^C_3_-HFPO-DA |
| NFDHA (3,6-OPFHpA) | Nonafluoro-3,6-dioxaheptanoic acid | 151772-58-6 | C_5_HF_9_O_4_ | ^13^C_5_-PFHxA |
| PFEESA | Perfluoro(2-ethoxyethane)sulfonic acid | 113507-82-7 | C_4_HF_9_O_4_S | ^13^C_5_-PFHxA |
| PFMPA (PF40PeA) | Perfluoro-3-methoxypropanoic acid | 377-73-1 | C_4_HF_7_O_3_ | ^13^C_5_-PFPeA |
| PFMBA (PF50HxA) | Perfluoro-4-methoxybutanoic acid | 863090-89-5 | C₅HF₉O₃ | ^13^C_5_-PFPeA |
| 9Cl-PF3ONS | 9-Chlorohexadecafluoro-3-oxanonane-1-sulfonic acid | 756426-58-1 | C_8_HClF_16_O_4_S | ^13^C_3_-HFPO-DA |
| 11Cl-PF3OUdS | 11-Chloroeicosafluoro-3-oxaundecane-1-sulfonic acid | 763051-92-9 | C_10_HClF_20_O_4_S | ^13^C_3_-HFPO-DA |
| 3:3 FTCA (FPrPA) | 3-Perfluoropropyl propanoic  acid | 356-02-5 | C_6_H_5_F_7_O_2_ | ^13^C_5_-PFPeA |
| 5:3 FTCA (FPePA) | 3-Perfluoropentyl propanoic acid | 914637-49-3 | C_8_H_5_F_11_O_2_ | ^13^C_5_-PFHxA |
| 7:3 FTCA (FHpPA) | 3-Perfluoroheptyl propanoic acid | 812-70-4 | C₁₀H₅F₁₅O₂ | ^13^C_5_-PFHxA |
| 4:2 FTS | Sodium 1H,1H,2H,2H-perfluorohexane sulfonate | 757124-72-4 | C_6_H_5_F_9_O_3_S | ^13^C_2_-4:2FTS |
| 6:2 FTS | Sodium 1H,1H,2H,2H-perfluorooctane sulfonate | 27619-97-2 | C_8_H_5_F_13_O_3_S | ^13^C_2_-6:2FTS |
| 8:2 FTS | Sodium 1H,1H,2H,2H-perfluorodecane sulfonate | 39108-34-4 | C_10_H_5_F_17_O_3_S | ^13^C_2_-8:2FTS |
| FOSA-1 | Perfluoro-1-octanesulfonamide | 754-91-6 | C_8_H_2_F_17_NO_2_S | ^13^C_8_-PFOSA |
| N-EtFOSAA | N-ethylperfluoro-1-octanesulfonamidoacetic acid | 2991-50-6 | C_12_H_8_F_17_NO_4_S | D_5_-N-EtFOSAA |
| N-MeFOSAA | N-methylperfluoro-1-octanesulfonamidoacetic acid | 2355-31-9 | C_11_H_6_F_17_NO_4_S | D_3_-NMeFOSAA |
| **Neutral PFAS – Analyzed by GC-EI-MS (UNC-CH Method)** | | | | |
| 6:2 FTOH | 2-(Perfluorohexyl)ethanol | 647-42-7 | C_8_H_5_F_13_O | ^13^C-6:2 FTOH |
| 8:2 FTOH | 2-(Perfluorooctyl)ethanol | 678-39-7 | C_10_H_5_F_17_O | ^13^C-8:2 FTOH |
| 10:2 FTOH | 2-(Perfluorodecyl)ethanol | 865-86-1 | C_12_H_5_F_21_O | ^13^C-10:2 FTOH |
| 8:2 FTAC | 2-(Perfluorooctyl)ethyl acrylate | 27905-45-9 | C_13_H_7_F_17_O_2_ | ^13^C-8:2 FTOH |
| 10:2 FTAC | 2-(Perfluorodecyl)ethyl acrylate | 17741-60-5 | C_15_H_7_F_21_O_2_ | ^13^C-10:2 FTOH |
| MeFOSA | N-Methylperfluorooctane sulfonamide | 31506-32-8 | C_9_H_4_F_17_NO_2_S | d-MeFOSA, d-EtFOSA |
| EtFOSA | N-Ethylperfluorooctane sulfonamide | 4151-50-2 | C_10_H_6_F_17_NO_2_S | d-EtFOSA |
| MeFOSE | N-Methyl-N-(2-hydroxyethyl)perfluorooctane sulfonamide | 24448-09-7 | C_11_H_8_F_17_NO_3_S | d^7^-MeFOSE |
| EtFOSE | N-Ethyl-N-(2-hydroxyethyl)perfluorooctane sulfonamide | 1691-99-2 | C_12_H_10_O_3_NSF_17_ | d^7^-MeFOSE |
|  |  |  |  |  |

**Section S1: Extraction and Analysis for Neutral PFAS**

All dust samples were extracted as described in Eichler et al. (2024). The analyzed dust mass varied by size fraction and ranged from ~25 mg for the smaller size fractions to ~500 mg for the >1180 µm size fraction. Briefly, each dust sample and field blank was spiked with 20 μL of a mass-labeled recovery standard mixture (5 ng/µL) and extracted three times in 3 mL 3:1 (v/v) hexane/methanol mix by sonication. Dust samples were each vortexed prior to sonication and centrifuged for 5 minutes at 4500 rpm after sonication. The supernatants were combined into 15 mL centrifuge tubes (Nunc Conical Sterile Polypropylene Centrifuge Tubes, Thermo Scientific, Fisher Scientific, Pittsburgh, PA) after each extraction. About 20 mg of ENVI-Carb (Supelclean™ ENVI-Carb™ SPE Bulk Packing, Supelco, Bellefonte, PA) was added to the extract, shaken for about 30 seconds, centrifuged (5 min, 4500 rpm), and filtered with a nylon syringe filter (13 mm diameter, 0.22 µm pore size; VWR, Radnor, PA) into a 15 mL PP centrifuge tube (Nunc Conical Sterile Polypropylene Centrifuge Tubes, Thermo Scientific, Fisher Scientific, Pittsburgh, PA). The filtrates were further evaporated to a final volume of ~300 µL. The final 300 µL was transferred to a PP autosampler vial (300 µL, Thermo Scientific, Fisher Scientific, Pittsburgh, PA) and used for analysis by gas chromatography (Agilent 8890 GC with DB-WAX column, 30 m, 0.25 mm ID, 0.25 µm film thickness) coupled with mass spectrometry (Agilent 5977B MS) in electron impact (EI) mode as described in Eichler et al. (2023). The extracts for nine neutral PFAS analytes and six neutral mass-labeled PFAS standards in selected-ion monitoring (SIM) mode. Each analyte and mass-labeled standard was quantified using authentic standards and a seven-point calibration curve ranging from 0.001 ng/μL to 1.0 ng/μL. Agilent Enhanced ChemStation (Version F.01.03.2357) software was used for peak integration.

Concentrations were corrected for recoveries of the mass-labeled standards, which ranged, on average, from 84% to 121% for the NC samples (see Eichler et al. (2024)) and from 42% to 101% for the NY dust samples (**Table S2**). Analytical precision obtained from repeat analysis of the NC dust samples and expressed as coefficients of variation, ranged from 5.6% to 36% (Eichler et al. 2024). Field blank concentrations were not subtracted from the sample concentrations. The Method Detection Limit (MDL) is based Instrument Detection Limit (IDL). MDLs are reported in **Table S3**. The IDL is the mean concentration plus three times the standard deviation for repeated injections of the lowest calibration standard used (0.001 ng/µL).

**Table S2**: Extraction efficiencies (recoveries) for the New York samples

|  | **13C-6:2 FTOH** | **13C-8:2 FTOH** | **13C-10:2 FTOH** | **d-EtFOSA** | **d7-MeFOSE** |
| --- | --- | --- | --- | --- | --- |
| **Mean** | 42% | 64% | 66% | 101% | 85% |
| **Std. dev.** | 12% | 15% | 16% | 30% | 17% |
| **N** | 46 | 46 | 46 | 46 | 46 |

**Table S3**: Method detection limits (MDLs) for neutral PFAS in dust analyzed by GC-MS as described in Eichler et al. (2024)

| **Compound** | **MDL (ng/g)** |
| --- | --- |
| 6:2 FTOH | 4.53 |
| 8:2 FTOH | 5.99 |
| 10:2 FTOH | 6.86 |
| 8:2 FTAC | 5.37 |
| 10:2 FTAC | 6.08 |
| EtFOSA | 6.96 |
| MeFOSA | 7.51 |
| MeFOSE | 1.72 |
| EtFOSE | 1.64 |

**Section S2: Extraction and Analysis for Ionic/Ionizable PFAS at UNC-CH**

For ionic/ionizable PFAS analysis in NC dust samples, ∼100 mg of sieved dust was placed into a 15 mL PP centrifuge tube. The dust was extracted three times by sonication in methanol (2 mL) for 15 min each time. ~50 mg of ENVI-Carb (Supelclean ENVI-Carb SPE Bulk Packing, Supelco, Bellefonte, PA) was added for extract cleanup before evaporation under nitrogen to 3-5 mL extract volume. Then, extracts were centrifuged (9 min, 4500 rpm) and filtered (nylon membrane, 13 mm diameter, 0.2 mm pore size, VWR, Radnor, PA) before evaporating further to 1 mL extract volume. Prior to analysis, a 25 mL aliquot of extract was combined with 75 mL of MilliQ water to match the initial mobile phase composition. Lab blanks consisted of extracted nylon sampling socks that remained in the lab. Method blanks were handled and processed the same way as other samples and blanks, but without sample media.

An AB SCIEX Triple Quad™ 6500 ultra-high performance liquid chromatography-electrospray ionization tandem mass spectrometry (UHPLC-ESI-MS/MS) operated in the negative mode with multiple reaction monitoring (MRM) was used to analyze the extracts for the 26 ionic/ionizable PFAS. Samples and blanks were quantified using a 5-point calibration curve (0.2 ng/mL to 20 ng/mL). All Teflon tubing within the SCIEX Triple Quad had been previously replaced with PEEK tubing to minimize contamination. A delay column (Zorbax RR Eclipse Plus, C18, 4.6 × 50 mm, 3.5 mm) was installed between the pump and injector. See Zhou et al. (2021) and Chang et al. (2025) for additional method parameters and operating details. Concentrations were recovery-corrected. Recoveries and analytical precision (as coefficients of variation) ranged from 52% to 172% and 9.6% to 70%, respectively, and were reported in detail in Chang et al. (2025). The MDL (**Table S4**) was based on the IDL, i.e., based on repeat injections of the lowest calibration standard used (0.2 ng/mL for PFCAs, PFSAs, and GenX and 1.0 ng/mL for PAPs).

**Table S4**: Method detection limits (MDLs) of the UNC-CH method used here for ionic/ionizable PFAS (LC-MS/MS). See also Chang et al. (2025)

| **Compound** | **MDL (ng/g)** | **Compound** | **MDL (ng/g)** |
| --- | --- | --- | --- |
| PFBA | 0.03 | PFBS | 0.10 |
| PFPeA | 0.02 | PFPeS | 0.06 |
| PFHxA | 0.02 | L-PFHxS | 0.06 |
| PFHpA | 0.03 | PFHpS | 0.03 |
| PFOA | 0.04 | L-PFOS | 0.04 |
| PFNA | 0.03 | PFNS | 0.04 |
| PFDA | 0.03 | PFDS | 0.13 |
| PFuDA | 0.09 | PFDoS | 0.46 |
| PFDoA | 0.48 | GenX | 0.39 |
| PFTrDA | 0.31 | 6:2 monoPAP | 0.44 |
| PFTeDA | 0.73 | 8:2 monoPAP | 0.33 |
| PFHxDA | 0.53 | 6:2 diPAP | 0.73 |
| PFODA | 0.04 | 8:2 diPAP | 0.31 |

**Section S3: Extraction and Analysis for Ionic/Ionizable PFAS at Clarkson**

Approximately 0.01 g of dust was transferred into a 15 mL polypropylene (PP) tube. The sample was spiked with 100 μL of a diluted mass-labelled extraction standard (MPFAC-HIF-ES, prepared by diluting 40 μL of the stock with 960 μL of methanol/water (75/25 v/v)). Next, 1.5 mL of methanol were added to the PP tube containing the spiked dust sample. The mixture was extracted by sonication for 10 minutes and subsequently centrifuged at 6,500 rpm for 10 minutes. 1 mL of the resulting supernatant was transferred into a 1.5 mL PP centrifuge. Approximately 10 mg of ENVI-Carb was added to the sample tube and vortexed for 30 seconds to remove matrix components. Centrifuged the sample at 6,500 rpm for 10 minutes, 0.75 mL was transferred into a 2 mL autosampler vial (ASV). To this, 0.25 mL of water and 50 μL of a diluted injection standard (MPFAC-HIF-IS, prepared by diluting 40 μL of the standard with 960 μL of solvent) were added. The solution was vortexed for 10 seconds to ensure thorough mixing. The prepared samples were analyzed by liquid chromatography-tandem mass spectrometry (LC-MS/MS; Thermo Vanquish LC coupled to a Thermo Altis MS) using a Phenomenex Luna Omega analytical column (100 x 2.1 mm, 1.6 um) and a Waters Acquity isolator column (50 x 2.1 mm, 1.6 um). Chromatographic separation was carried out with 5 mM ammonium acetate in water (mobile phase A) and acetonitrile (mobile phase B) at a flow rate of 0.5 mL/min. The LC gradient was as follows: 0 min (30% B), 0.5 min (30% B), 3 min (90% B), 3.1 min (100% B), 4.5 min (100% B), 4.6 min (30% B) and 6.5 min (30% B). MS parameters were optimized based on multiple reaction monitoring (MRM) transitions (precursor/product fragment ion pair) consistent with EPA Method 1633A (USEPA 2024). Sample acquisition was carried out with Thermo XCalibur 4.7 software and data analysis was performed with Thermo TraceFinder 5.2 software. Concentrations were recovery corrected according to EPA Method 1633A (USEPA 2024). All recoveries were within the acceptable ranges defined in Table 8 for soil/sediment samples in EPA Method 1633A. These ranges are PFAS-dependent, but range from 40% to 130% for most PFAS discussed here. Field blank concentrations were not subtracted. MDLs were calculated as sample-spiked MDLs (i.e., MDL_s_, in contrast to method blank-spiked MDLs = MDL_b_; see Table 9 in EPA Method 1633A and USEPA (2016)) and are listed in **Table S5**.

**Table S5**: Method detection limits (MDLs) of the Clarkson method (LC-MS/MS). MDLs were established as sample-spiked MDLs as described in EPA Method 1633A (USEPA 2024) and in USEPA (2016)

| **Compound** | **MDL (ng/g)** | **Compound** | **MDL (ng/g)** |
| --- | --- | --- | --- |
| PFBA | 14.19 | PFDS | 4.36 |
| PFPeA | 3.46 | PFDoS | 5.82 |
| PFHxA | 5.22 | GenX | 30.34 |
| PFHpA | 3.48 | NaDONA | 5.07 |
| PFOA | 4.55 | NFDHA (3,6-OPFHpA) | 83.85 |
| PFNA | 4.42 | PFEESA | 5.79 |
| PFDA | 4.69 | PFMPA (PF40PeA) | 4.17 |
| PFuDA | 3.57 | PFMBA (PF50HxA) | 4.47 |
| PFDoA | 4.69 | 9Cl-PF3ONS | 4.51 |
| PFTrDA | 3.79 | 11Cl-PF3OUdS | 4.58 |
| PFTeDA | 4.20 | 3:3 FTCA (FPrPA) | 45.48 |
| PFBS | 1.09 | 5:3 FTCA (FPePA) | 27.16 |
| PFPeS | 3.28 | 7:3 FTCA (FHpPA) | 34.07 |
| L-PFHxS | 7.44 | 4:2 FTS | 1.83 |
| Br-PFHxS | 3.91 | 6:2 FTS | 9.06 |
| PFHpS | 4.72 | 8:2 FTS | 8.40 |
| L-PFOS | 4.63 | FOSA | 2.80 |
| Br-[PFOS](https://pubchem.ncbi.nlm.nih.gov/#query=C4HF7O3) | 4.51 | EtFOSAA | 8.17 |
| PFNS | 4.23 | MeFOSAA | 10.36 |

**Section S4: Comparison of the Results from the UNC-CH Method and Clarkson Method**

A small subset of NC samples was analyzed using the UNC-CH method (**Section S2**) as well as the Clarkson method (**Section S3**). These samples also included NC samples from homes 01, 35, and 65 collected at the beginning (t = 0) of the IPA Campaign (see Chang et al. (2025)). As shown in **Figure S1**, there were differences between the results obtained using the two different methods, particularly for certain compounds. While some general trends are similar, e.g., low total PFAS levels in the t = 0 and t = 6 months samples from home 35, the occurrences of some individual PFAS (e.g., L-PFOS) are quite different. For this reason, we refrain from comparing the concentrations of ionic/ionizable PFAS in NC samples and NY samples directly, and use normalized concentrations for further statistical analysis.


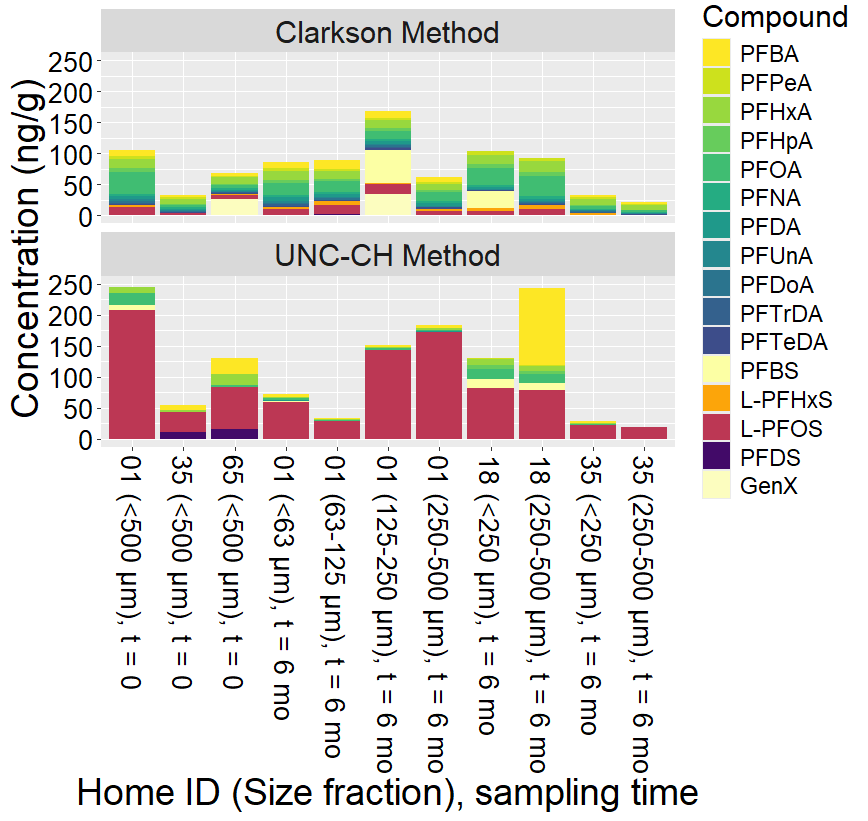


**Figure S1**: Results for the same samples analyzed by the Clarkson method and the UNC-CH method.

**Section S5: Extractable Organic Fluorine (EOF) Analysis**

Approximately 0.05 g of dust was weighed and transferred into a 15 mL polypropylene (PP) tube. Then 1.5 mL of methanol was added to the PP tube and each sample was sonicated for 10 minutes. Following sonication, the tube was centrifuged at 6,500 rpm for 10 minutes to separate the supernatant. The supernatant was carefully transferred into a 50 mL tube, and 15 mL of LC-MS-grade water was added to dilute the extract. Solid-phase extraction (SPE) was then performed on this diluted extract as described below in order to separate the inorganic fluoride in the sample from the organic fluorine.

SPE Cartridge Conditioning: SPE cartridges (Oasis WAX 6cc cartridges, 500 mg sorbent, 60 µm particle size, Waters) were conditioned prior to sample loading using a sequential process. Each cartridge was conditioned with 5 mL of methanol (MeOH) containing 1% ammonium hydroxide (NH₄OH), followed by 10 mL of MeOH, and finally 5 mL of LC-MS grade water at a flow rate of 1–2 mL/min (approximately one drop per second). During the final water step, a small amount of water was left on top of the cartridge to prevent the sorbent from drying before the sample loading step. All the conditioning and elution steps had the same flow rate.

Sample Loading: The diluted sample extracts were hand-loaded via pipetting 5 mL aliquots onto the conditioned cartridges. The remaining sample volume was drained using a slight vacuum if necessary, until dripping stopped, ensuring the sorbent remained moist.

Inorganic Fraction Elution: The inorganic fluoride fraction was eluted by adding 5 mL of 0.01% NH₄OH in water to the cartridge, followed by 5 mL of LC-MS grade water. The cartridge was allowed to drain until the dripping stopped but was not dried completely.

Organic Fraction Elution: The organic fluorine fraction was eluted into a 15 mL centrifuge tube using 5 mL of MeOH containing 1% NH₄OH as the elution solvent. Before cartridge elution, the sample container was rinsed with the 5 mL elution solvent to recover any PFAS adhered to the container walls. The elution solvent was then loaded onto the cartridge. Approximately 1 mL of the elution solvent was allowed to drain, after which the cartridge was soaked for 5 minutes. The remaining solvent was then drained completely into the collection tube.

Total Organic Fluorine (TOF) Analysis: An aliquot of 700 µL from the 5 mL organic fraction extract was analyzed for total organic fluorine (TOF) using Combustion Ion Chromatography (CIC). An integrated ion chromatography system was employed to analyze the nontarget PFAS by quantifying the TOF, consisting of components manufactured by **Metrohm, Switzerland**. The system included a **Compact IC Flex 930** equipped with **conductivity detection** and a **920 Absorber Module**. For sample preparation, a **combustion module** (Analytik Jena, Germany) was utilized to convert samples into inorganic fluoride via combustion at **1050 °C,** and transferred to the absorber module via argon gas. The absorber solution (Milli-Q water) containing the inorganic fluoride is then analyzed via ion separation on a Metrosep A **Supp 5 150/4.0 analytical column** combined with a **Metrosep A Supp 4/5 guard column.** A **1 mL sample loop** was used for sample injection, and the column oven was maintained at **30 °C.** The mobile phase consisted of **0.32 M sodium carbonate / 0.10 M sodium bicarbonate**. The carrier gas was **argon** at a flow rate of **100 mL/min**, and the combustion gas was **oxygen** at **300 mL/min.** The injection volume was **900 μL**. All solutions were prepared using **Milli-Q water** (18 MΩ·cm; 0.56 μS/cm) to ensure high purity.

Method detection limit (MDL): 20 ng/mg = 20,000 ng/g

**Section S6: Calculation of Weighted Sums**

The concentration of PFAS in the <500 µm fraction (NC homes) was calculated as follows:

$$c_{i,<500\mu m}=f_{<63\mu m}\cdot c_{i,<63\mu m}+f_{63-125\mu m}\cdot c_{i,63-125\mu m}+f_{125-250\mu m}\cdot c_{i,125-250\mu m}+f_{250-500\mu m}\cdot c_{i,250-500\mu m}$$

or

$$c_{i,<500\mu m}=f_{<250\mu m}\cdot c_{i,<250\mu m}+f_{250-500\mu m}\cdot c_{i,250-500\mu m}$$

where *c_i_* is the concentration of PFAS *i* in a given size fraction (e.g., <63 µm) and *f* is the fraction of mass of this size fraction, i.e., $f_{j}=m_{j}/m_{<500\mu m}$ where *j* is any of the size fractions used for the NC samples.

Accordingly, the concentration of PFAS in the <600 µm fraction (NY homes) was calculated as

$$c_{i,<600\mu m}=f_{<75\mu m}\cdot c_{i,<75\mu m}+f_{75-150\mu m}\cdot c_{i,75-150\mu m}+f_{150-250\mu m}\cdot c_{i,150-250\mu m}+f_{250-600\mu m}\cdot c_{i,250-600\mu m}$$

with $f_{k}=m_{k}/m_{<600\mu m}$ where *k* is any of the size fractions used for the NY samples.


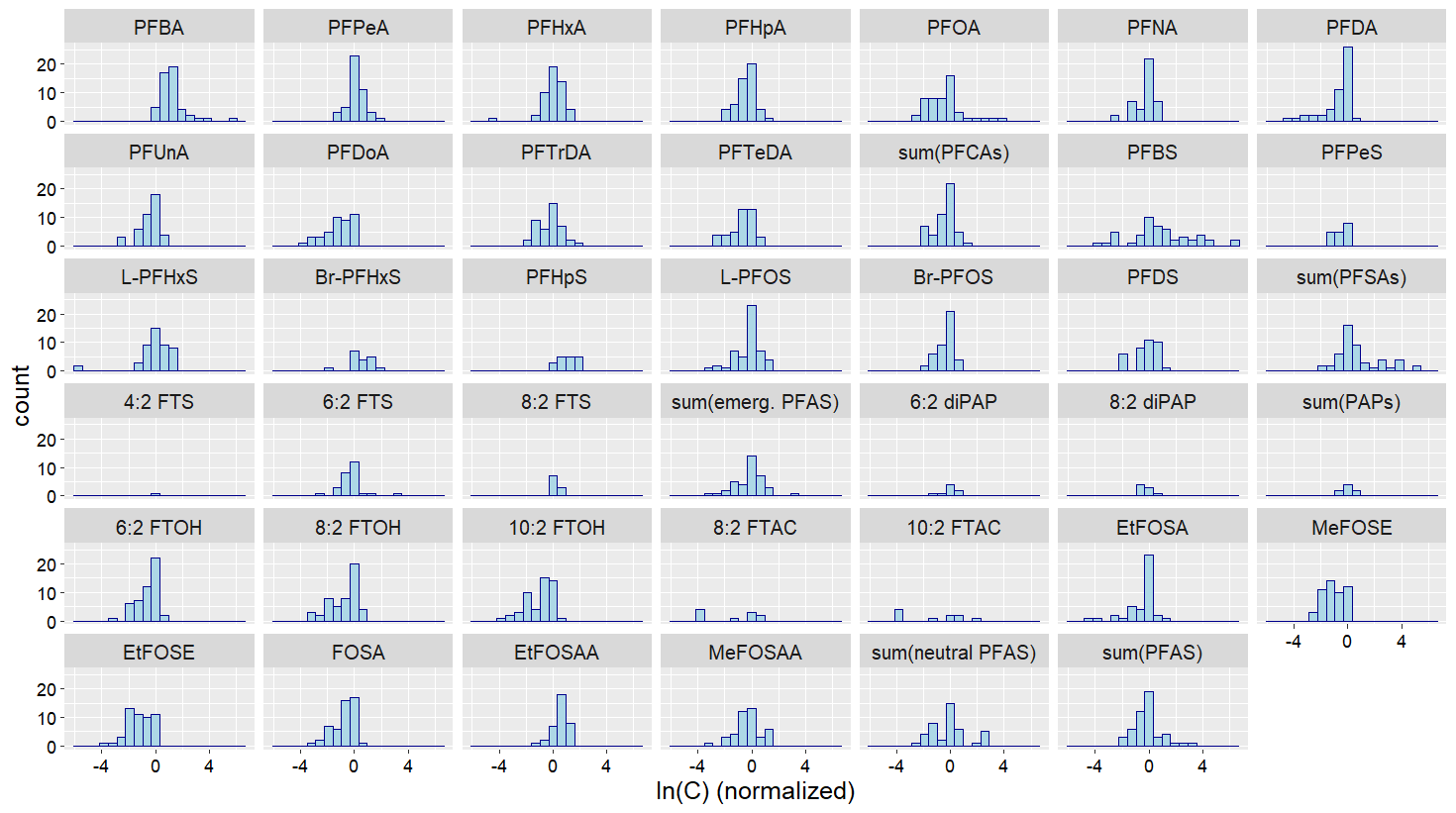


**Figure S2**: Histograms showing the distributions of the ln-transformed, normalized concentrations of targeted PFAS across all dust samples. Non-detects were replaced by MDL/2 prior to ln-transformation.

**Table S6**: PFAS measured in dust samples and field blanks (FBs) from New York (NY) homes using the Clarkson method for ionic/ionizable PFAS (LC-MS/MS). “nd”: not detected

| **Home ID** | **Size fraction** | **PFBA** | **PFPeA** | **PFHxA** | **PFHpA** | **PFOA** | **PFNA** | **PFDA** | **PFuDA** | **PFDoA** | **PFTrDA** | **PFTe-DA** | **PFBS** | **PFPeS** | **L-PFHxS** | **Br-PFHxS** | **PFHpS** | **L-PFOS** | **Br-PFOS** | **PFNS** |
| --- | --- | --- | --- | --- | --- | --- | --- | --- | --- | --- | --- | --- | --- | --- | --- | --- | --- | --- | --- | --- |
|  |  | **ng/g** | | | | | | | | | | | | | | | | | | |
|  | *MDL* | *14.19* | *3.46* | *5.22* | *3.48* | *4.55* | *4.42* | *4.69* | *3.57* | *4.69* | *3.79* | *4.20* | *1.09* | *3.28* | *7.44* | *3.91* | *4.72* | *4.63* | *4.51* | *4.23* |
| C1 | >1180 µm | 10.7 | 6.56 | 6.22 | 1.90 | 6.04 | 1.38 | 4.15 | 1.38 | 2.07 | 1.04 | 1.21 | 120 | nd | 2.59 | nd | nd | 6.56 | 3.63 | nd |
| C1 | 600-1180 µm | 6.42 | 5.37 | 5.97 | 3.13 | 3.88 | 2.69 | 5.52 | 1.64 | 2.54 | nd | 1.64 | 40.5 | nd | 1.34 | nd | nd | 4.18 | nd | nd |
| C1 | <600 µm* |  |  |  |  |  |  |  |  |  |  |  |  |  |  |  |  |  |  |  |
| C1 | 250-600 µm | 1.24 | 2.30 | 7.61 | 1.42 | 6.37 | 3.19 | 7.61 | 3.89 | 9.91 | 2.12 | 2.12 | 25.0 | nd | nd | nd | nd | 14.9 | 3.72 | nd |
| C1 | 150-250 µm | 7.49 | 9.81 | 4.64 | 5.89 | 5.71 | 2.85 | 1.43 | 4.46 | nd | nd | 1.25 | nd | nd | 10.4 | 5.89 | nd | nd | nd | nd |
| C1 | 75-150 µm | 1.39 | 1.86 | 6.35 | 2.17 | 10.2 | 1.24 | 2.79 | nd | 1.24 | 1.70 | 1.24 | nd | nd | nd | nd | nd | 13.2 | 4.18 | nd |
| C1 | <75 µm | 14.7 | 6.50 | 9.50 | 11.3 | 36.7 | 13.2 | 83.8 | 16.8 | 61.3 | 6.33 | 19.3 | 8.67 | 5.83 | 6.17 | 1.17 | 1.00 | 43.8 | 13.3 | nd |
| C2 | >1180 µm | 10.5 | 8.01 | 14.8 | 7.14 | 9.23 | 7.67 | 5.92 | 2.96 | 3.48 | 1.57 | 3.83 | 290 | nd | nd | nd | nd | 2.26 | nd | nd |
| C2 | 600-1180 µm | 5.92 | 4.36 | 6.27 | 1.22 | 7.84 | 1.74 | 1.92 | 0.87 | 0.35 | 0.70 | 0.87 | 17.94 | nd | nd | nd | nd | 1.57 | nd | nd |
| C2 | <600 µm* |  |  |  |  |  |  |  |  |  |  |  |  |  |  |  |  |  |  |  |
| C2 | 250-600 µm | 1.84 | 2.17 | 7.02 | 2.01 | 4.85 | 2.17 | 2.51 | 1.67 | 1.00 | nd | nd | 29.8 | nd | 1.67 | nd | nd | 10.7 | 2.84 | nd |
| C2 | 150-250 µm | 7.49 | 6.07 | 14.8 | 7.49 | 9.81 | 4.64 | 5.89 | 5.71 | 2.85 | 1.43 | 4.46 | nd | nd | 1.25 | nd | nd | 10.4 | 5.89 | nd |
| C2 | 75-150 µm | 13.1 | 10.2 | 11.9 | 7.85 | 2.92 | 5.84 | 7.85 | 5.47 | 4.93 | 6.20 | 3.28 | 44.2 | nd | 2.19 | 0.36 | 1.64 | 6.02 | 2.19 | nd |
| C2 | <75 µm | 7.81 | 10.0 | 8.29 | 5.71 | 8.19 | 4.29 | 7.14 | 3.24 | 5.14 | 3.43 | 5.14 | 9.90 | nd | 1.14 | nd | 0.29 | 6.86 | 4.67 | nd |
| C3 | >1180 µm | 10.6 | 4.31 | 23.1 | 11.6 | 15.9 | 1.66 | 2.82 | 1.49 | 1.33 | 0.50 | 1.82 | 3973 | nd | nd | nd | 0.66 | 6.80 | 2.82 | nd |
| C3 | 600-1180 µm | 6.24 | 5.92 | 8.32 | 4.96 | 4.16 | 2.24 | 4.96 | 1.28 | 1.60 | 0.48 | 0.64 | 7.20 | nd | 1.12 | nd | nd | 4.16 | 0.80 | nd |
| C3 | <600 µm* |  |  |  |  |  |  |  |  |  |  |  |  |  |  |  |  |  |  |  |
| C3 | 250-600 µm | 2.83 | 2.20 | 7.56 | 1.26 | 35.4 | 2.52 | 3.31 | 2.20 | 0.47 | 0.31 | 1.26 | nd | nd | 0.79 | nd | nd | 11.3 | 5.67 | nd |
| C3 | 150-250 µm | 16.9 | 12.0 | 11.4 | 3.81 | 6.66 | 2.09 | 2.28 | 0.95 | 0.76 | 0.38 | 0.95 | 200 | nd | nd | nd | nd | 5.90 | 5.52 | nd |
| C3 | 75-150 µm | 8.60 | 5.01 | 13.5 | 5.30 | 12.8 | 2.72 | 3.44 | 1.00 | 0.72 | 1.43 | 1.15 | 48.6 | 0.86 | 2.58 | nd | nd | 6.73 | 3.15 | nd |
| C3 | <75 µm | 3.04 | 1.83 | 4.96 | 3.22 | 0.87 | 1.22 | 3.48 | 1.22 | 2.26 | 0.52 | 1.74 | 5.91 | nd | 1.74 | nd | nd | 7.13 | 3.39 | nd |
| C4 | >1180 µm | 11.2 | 5.45 | 9.74 | 3.63 | 6.60 | 2.97 | 3.47 | 1.49 | 0.50 | nd | nd | 847 | nd | 0.66 | nd | nd | 6.60 | nd | nd |
| C4 | 600-1180 µm | 3.21 | 3.39 | 6.79 | 0.71 | 10.9 | 2.68 | 2.14 | 1.43 | nd | 1.61 | 1.61 | 1275 | nd | nd | nd | nd | 5.54 | nd | nd |
| C4 | <600 µm* |  |  |  |  |  |  |  |  |  |  |  |  |  |  |  |  |  |  |  |
| C4 | 250-600 µm | 3.07 | 2.93 | 6.44 | 2.05 | 4.97 | 1.17 | 1.61 | nd | 0.59 | nd | 0.88 | nd | nd | nd | nd | nd | 9.07 | 0.88 | nd |
| C4 | 150-250 µm | 8.74 | 7.09 | 7.75 | 2.14 | 7.75 | 3.13 | 7.25 | 1.32 | 1.98 | 1.65 | 1.81 | 40.1 | nd | 1.98 | nd | nd | 7.75 | 0.82 | nd |
| C4 | 75-150 µm | 14.1 | 7.71 | 7.88 | 3.77 | 4.80 | 3.43 | 3.43 | 2.40 | 2.40 | 0.86 | 2.06 | 38.7 | nd | nd | nd | nd | 1.89 | 2.57 | nd |
| C4 | <75 µm | 8.86 | 4.48 | 11.2 | 5.24 | 36.38 | 3.33 | 8.00 | 2.29 | 6.10 | 1.24 | 5.14 | 21.5 | nd | 0.95 | nd | nd | 6.38 | 4.00 | nd |
| C5 | >1180 µm | 16.7 | 4.19 | 7.75 | 2.33 | 8.37 | 2.48 | 1.71 | 0.78 | 1.40 | 1.40 | 1.09 | 581 | nd | nd | nd | nd | 2.79 | 4.50 | nd |
| C5 | 600-1180 µm | 11.9 | 3.45 | 11.7 | 2.07 | 6.55 | 3.28 | 5.34 | 1.72 | 2.07 | 1.90 | 1.38 | 491 | nd | 1.55 | nd | nd | 5.86 | nd | nd |
| C5 | <600 µm* |  |  |  |  |  |  |  |  |  |  |  |  |  |  |  |  |  |  |  |
| C5 | 250-600 µm | 3.48 | 2.53 | 7.28 | 1.74 | 4.28 | 1.74 | 1.74 | 0.79 | 0.79 | 1.27 | 0.48 | nd | nd | nd | nd | nd | 13.5 | 2.53 | nd |
| C5 | 150-250 µm | 25.6 | 6.89 | 7.80 | 2.18 | 10.9 | 3.08 | 3.81 | 1.09 | 1.99 | 1.99 | 1.09 | 108 | nd | 1.27 | nd | nd | 3.63 | 2.36 | nd |
| C5 | 75-150 µm | 12.9 | 2.24 | 11.55 | 2.59 | 12.9 | 3.28 | 3.10 | 1.21 | 0.86 | 0.52 | 1.90 | nd | nd | 2.24 | nd | nd | 12.6 | 1.72 | nd |
| C5 | <75 µm | 7.18 | 2.91 | 3.73 | 1.73 | 25.09 | 2.55 | 4.09 | 1.64 | 2.36 | 1.09 | 1.73 | 8.45 | nd | nd | nd | nd | 3.27 | 2.18 | nd |
| C6 | >1180 µm | 6.88 | 3.93 | 7.37 | 0.98 | 10.2 | 1.80 | 3.44 | 0.98 | nd | 0.49 | 1.15 | 972 | nd | 1.31 | nd | nd | 2.78 | nd | nd |
| C6 | 600-1180 µm | 8.47 | 2.37 | 10.51 | 2.88 | 11.2 | 2.54 | 3.05 | 1.02 | 0.34 | 0.34 | 1.53 | 4374 | nd | 1.02 | nd | nd | 13.4 | 2.54 | nd |
| C6 | <600 µm* |  |  |  |  |  |  |  |  |  |  |  |  |  |  |  |  |  |  |  |
| C6 | 250-600 µm | 8.48 | 5.86 | 8.79 | 2.47 | 4.32 | 1.85 | 3.55 | 1.08 | 1.70 | nd | 0.46 | 40.9 | nd | nd | nd | nd | 5.55 | nd | nd |
| C6 | 150-250 µm | 2.73 | 3.21 | 8.03 | 1.77 | 6.43 | 2.41 | 2.09 | 1.12 | 0.80 | 0.96 | 0.80 | 24.1 | nd | 0.64 | nd | nd | 18.2 | 3.37 | nd |
| C6 | 75-150 µm | 6.77 | 6.11 | 8.75 | 1.49 | 12.06 | 2.15 | 2.15 | 2.64 | 0.83 | 0.83 | 1.32 | 108 | nd | 1.49 | nd | nd | 2.81 | nd | nd |
| C6 | <75 µm | 7.00 | 2.33 | 4.56 | 2.44 | 5.67 | 1.67 | 3.33 | 1.56 | 2.56 | 0.33 | 0.78 | 9.89 | nd | 1.22 | nd | 0.89 | 6.78 | 2.67 | nd |
| C7 | >1180 µm | 10.8 | 8.98 | 9.87 | 4.12 | 14.3 | 3.68 | 4.12 | 1.91 | 2.21 | 1.03 | 2.95 | 504 | nd | 1.47 | nd | nd | 3.09 | 1.62 | nd |
| C7 | 600-1180 µm | 3.01 | 2.26 | 2.63 | 2.13 | 13.7 | 3.64 | 4.76 | 2.38 | 1.76 | 0.50 | 1.00 | 4.51 | 3.01 | 0.38 | nd | nd | 0.88 | nd | nd |
| C7 | <600 µm* |  |  |  |  |  |  |  |  |  |  |  |  |  |  |  |  |  |  |  |
| C7 | 250-600 µm | 7.68 | 6.63 | 9.60 | 6.98 | 7.50 | 12.2 | 7.68 | 6.46 | 3.66 | 1.22 | 3.66 | 106 | nd | nd | nd | nd | 3.49 | nd | nd |
| C7 | 150-250 µm | 11.7 | 7.18 | 13.4 | 5.51 | 4.67 | 8.01 | 9.68 | 7.01 | 5.84 | 1.34 | 3.51 | 48.1 | nd | nd | nd | 0.83 | 9.85 | 4.17 | nd |
| C7 | 75-150 µm | 9.15 | 2.96 | 9.01 | 4.65 | 3.52 | 5.21 | 4.37 | 2.11 | 1.83 | 1.27 | 3.52 | 55.4 | nd | 0.85 | nd | nd | 5.63 | 1.13 | nd |
| C7 | <75 µm | 12.7 | 5.14 | 10.9 | 10.3 | 14.4 | 14.4 | 10.6 | 8.43 | 9.71 | 3.57 | 8.86 | 19.3 | 3.43 | 1.00 | 0.57 | nd | 11.6 | 2.43 | nd |
| C1 | FB* | nd | 1.67 | nd | 1.67 | 12.5 | nd | nd | nd | nd | nd | 1.67 | 183 | nd | 1.00 | nd | nd | 13.7 | nd | nd |
| C2 | FB* | nd | 1.54 | 6.14 | 0.92 | 6.91 | nd | nd | nd | nd | nd | nd | nd | nd | nd | nd | nd | 8.60 | 2.15 | nd |
| C3 | FB* | 0.42 | 1.98 | 5.52 | 1.42 | 4.67 | 1.70 | nd | nd | 1.84 | nd | 0.85 | nd | nd | nd | nd | nd | 11.5 | 2.55 | nd |
| C4 | FB* | 0.88 | nd | 5.72 | 1.32 | 6.02 | 1.47 | nd | nd | nd | 0.59 | nd | nd | nd | 1.47 | nd | nd | 8.80 | 2.93 | nd |
| C5 | FB* | 1.17 | nd | 5.14 | 1.03 | 7.19 | nd | nd | nd | nd | 1.32 | nd | 36.5 | nd | nd | nd | nd | 6.46 | 1.17 | nd |
| C6 | FB* | 0.91 | 1.96 | 5.13 | 1.66 | 5.58 | 1.36 | nd | nd | nd | 0.60 | nd | nd | nd | nd | nd | nd | 9.05 | nd | nd |
| C7 | FB* | 1.18 | nd | nd | 1.03 | 9.76 | 1.63 | nd | nd | nd | 2.07 | nd | nd | nd | nd | nd | nd | 7.54 | 2.22 | nd |
| **Home ID** | **Size fraction** | **PFDS** | **PFDoS** | **GenX** | **Na-DONA** | **NFDHA** | **PFEESA** | **PFMPA (PF40-PeA)** | **PFMBA (PF50-HxA)** | **9Cl-PF3ONS** | **11Cl-PF3O-UdS** | **3:3 FTCA (FPrPA)** | **5:3 FTCA (FPe-PA)** | **7:3 FTCA (FHp-PA)** | **4:2 FTS** | **6:2 FTS** | **8:2 FTS** | **FOSA** | **Et-FOSAA** | **Me-FOSAA** |
|  |  | **ng/g** | | | | | | | | | | | | | | | | | | |
|  | *MDL* | *4.36* | *5.82* | *30.34* | *5.07* | *83.85* | *5.79* | *4.17* | *4.47* | *4.51* | *4.58* | *45.48* | *27.16* | *34.07* | *1.83* | *9.06* | *8.40* | *2.80* | *8.17* | *10.36* |
| C1 | >1180 µm | nd | nd | nd | nd | nd | 0.69 | nd | nd | nd | nd | nd | nd | 7.60 | nd | nd | nd | nd | 1.73 | 1.90 |
| C1 | 600-1180 µm | nd | nd | nd | nd | nd | nd | nd | nd | nd | nd | nd | nd | nd | nd | 0.90 | nd | nd | nd | nd |
| C1 | <600 µm* |  |  |  |  |  |  |  |  |  |  |  |  |  |  |  |  |  |  |  |
| C1 | 250-600 µm | 1.77 | nd | nd | nd | nd | nd | nd | nd | nd | nd | nd | nd | nd | nd | nd | nd | nd | nd | nd |
| C1 | 150-250 µm | nd | nd | nd | nd | nd | nd | nd | nd | nd | nd | nd | 3.21 | nd | nd | 90.5 | nd | nd | 6.85 | 4.44 |
| C1 | 75-150 µm | nd | nd | nd | nd | nd | nd | nd | nd | nd | nd | nd | nd | nd | nd | nd | nd | nd | 2.32 | nd |
| C1 | <75 µm | 13.0 | nd | nd | nd | nd | 0.50 | nd | nd | nd | nd | nd | nd | nd | nd | 2.83 | 0.67 | 1.00 | 15.3 | 33.0 |
| C2 | >1180 µm | nd | nd | nd | nd | nd | nd | nd | nd | nd | nd | nd | nd | nd | nd | 4.18 | nd | 0.52 | 76.0 | nd |
| C2 | 600-1180 µm | nd | nd | nd | nd | nd | nd | nd | nd | nd | nd | nd | nd | nd | nd | 0.52 | nd | 0.35 | 2.96 | nd |
| C2 | <600 µm* |  |  |  |  |  |  |  |  |  |  |  |  |  |  |  |  |  |  |  |
| C2 | 250-600 µm | nd | nd | nd | nd | nd | nd | nd | nd | nd | nd | nd | nd | nd | nd | 2.01 | nd | nd | 25.8 | nd |
| C2 | 150-250 µm | nd | nd | nd | nd | nd | nd | nd | nd | nd | nd | nd | nd | nd | nd | 3.21 | nd | nd | 90.5 | nd |
| C2 | 75-150 µm | nd | nd | nd | nd | nd | nd | nd | nd | nd | nd | nd | nd | 4.38 | nd | 4.74 | nd | nd | 137 | 1.46 |
| C2 | <75 µm | 4.76 | nd | nd | nd | nd | 0.38 | 1.90 | nd | nd | nd | nd | nd | nd | nd | 8.38 | 1.52 | 0.76 | 92.1 | 3.62 |
| C3 | >1180 µm | 2.32 | nd | nd | nd | nd | 0.83 | nd | nd | nd | nd | nd | nd | 6.30 | nd | 0.83 | nd | nd | 6.14 | 0.83 |
| C3 | 600-1180 µm | 0.64 | nd | nd | nd | nd | 0.48 | nd | nd | nd | nd | nd | nd | nd | nd | 0.96 | nd | 0.48 | 4.96 | 0.48 |
| C3 | <600 µm* |  |  |  |  |  |  |  |  |  |  |  |  |  |  |  |  |  |  |  |
| C3 | 250-600 µm | nd | nd | nd | nd | nd | 0.63 | nd | nd | nd | nd | nd | nd | 1.57 | nd | nd | nd | nd | nd | nd |
| C3 | 150-250 µm | nd | nd | nd | nd | nd | nd | nd | nd | nd | nd | nd | nd | nd | nd | 1.33 | nd | nd | 10.7 | 0.57 |
| C3 | 75-150 µm | 8.88 | nd | nd | nd | nd | 0.57 | nd | nd | nd | nd | nd | nd | nd | nd | nd | nd | nd | 14.8 | 1.72 |
| C3 | <75 µm | 5.22 | nd | nd | nd | nd | 0.26 | nd | nd | nd | nd | nd | nd | nd | nd | 1.57 | 0.61 | 0.35 | 4.43 | 2.26 |
| C4 | >1180 µm | nd | nd | nd | nd | nd | nd | nd | nd | nd | nd | nd | nd | 1.65 | nd | 1.65 | nd | nd | 3.63 | nd |
| C4 | 600-1180 µm | nd | nd | nd | nd | nd | nd | nd | nd | nd | nd | nd | nd | nd | nd | nd | nd | nd | nd | 1.07 |
| C4 | <600 µm* |  |  |  |  |  |  |  |  |  |  |  |  |  |  |  |  |  |  |  |
| C4 | 250-600 µm | nd | nd | nd | nd | nd | 0.59 | nd | nd | nd | nd | nd | nd | 3.51 | nd | nd | nd | nd | nd | nd |
| C4 | 150-250 µm | nd | nd | nd | nd | nd | nd | nd | nd | nd | nd | nd | nd | 3.96 | nd | nd | nd | nd | 8.24 | nd |
| C4 | 75-150 µm | nd | nd | nd | nd | nd | 0.69 | nd | nd | nd | nd | nd | nd | 4.63 | nd | 1.89 | nd | nd | 6.00 | nd |
| C4 | <75 µm | 2.48 | nd | nd | nd | nd | 0.48 | nd | nd | nd | nd | nd | nd | nd | nd | 2.19 | 0.10 | 0.57 | 10.6 | 3.81 |
| C5 | >1180 µm | nd | nd | nd | nd | nd | nd | nd | nd | nd | nd | nd | nd | nd | nd | 0.47 | nd | nd | nd | nd |
| C5 | 600-1180 µm | nd | nd | nd | nd | nd | 0.69 | nd | nd | nd | nd | nd | nd | 3.10 | nd | nd | 1.38 | nd | nd | 0.86 |
| C5 | <600 µm* |  |  |  |  |  |  |  |  |  |  |  |  |  |  |  |  |  |  |  |
| C5 | 250-600 µm | nd | nd | nd | nd | nd | 0.63 | nd | nd | nd | nd | nd | nd | 5.86 | nd | nd | nd | nd | nd | 0.48 |
| C5 | 150-250 µm | nd | nd | nd | nd | nd | 0.73 | nd | nd | nd | nd | nd | nd | 2.72 | nd | nd | nd | nd | nd | 0.91 |
| C5 | 75-150 µm | nd | nd | nd | nd | nd | nd | nd | nd | nd | nd | nd | nd | 3.10 | nd | nd | nd | nd | nd | 1.38 |
| C5 | <75 µm | nd | nd | nd | nd | nd | 0.27 | 1.82 | nd | nd | nd | nd | nd | nd | 1.27 | 1.18 | 0.82 | nd | 1.18 | 0.82 |
| C6 | >1180 µm | nd | nd | nd | nd | nd | nd | nd | nd | nd | nd | nd | nd | 5.24 | nd | 0.98 | nd | nd | 1.31 | nd |
| C6 | 600-1180 µm | 2.37 | nd | nd | nd | nd | nd | nd | nd | 0.68 | nd | nd | nd | nd | nd | 2.37 | nd | nd | 5.93 | nd |
| C6 | <600 µm* |  |  |  |  |  |  |  |  |  |  |  |  |  |  |  |  |  |  |  |
| C6 | 250-600 µm | nd | nd | nd | nd | nd | nd | nd | nd | nd | nd | nd | nd | 7.09 | nd | 0.46 | 3.39 | nd | 4.01 | nd |
| C6 | 150-250 µm | nd | nd | nd | nd | nd | 0.64 | nd | nd | nd | nd | nd | nd | nd | nd | nd | nd | nd | 4.18 | nd |
| C6 | 75-150 µm | nd | nd | nd | nd | nd | 0.66 | nd | nd | nd | nd | nd | nd | nd | nd | 4.79 | nd | 0.33 | 4.29 | nd |
| C6 | <75 µm | 1.44 | nd | nd | nd | nd | 0.33 | nd | nd | nd | nd | nd | nd | nd | nd | 1.78 | 1.44 | 0.44 | 9.89 | 0.33 |
| C7 | >1180 µm | nd | nd | nd | nd | nd | 0.59 | nd | nd | nd | nd | nd | nd | 5.45 | nd | 3.09 | nd | nd | nd | 0.88 |
| C7 | 600-1180 µm | nd | nd | nd | nd | nd | nd | nd | nd | nd | nd | nd | nd | nd | nd | nd | nd | 0.25 | nd | nd |
| C7 | <600 µm* |  |  |  |  |  |  |  |  |  |  |  |  |  |  |  |  |  |  |  |
| C7 | 250-600 µm | nd | nd | nd | nd | nd | nd | nd | nd | nd | nd | nd | nd | 6.81 | nd | nd | nd | nd | 2.97 | 1.05 |
| C7 | 150-250 µm | nd | nd | nd | nd | nd | nd | nd | nd | nd | nd | nd | nd | nd | nd | 1.00 | nd | nd | nd | 0.83 |
| C7 | 75-150 µm | 4.08 | nd | nd | nd | nd | 0.70 | nd | nd | nd | nd | nd | nd | nd | nd | 1.83 | 0.85 | nd | 6.48 | 0.85 |
| C7 | <75 µm | 1.00 | nd | nd | nd | nd | 0.57 | nd | nd | nd | nd | nd | nd | nd | nd | 1.86 | 0.43 | 0.86 | 5.71 | 3.86 |
| C1 | FB* | nd | nd | nd | nd | nd | nd | nd | nd | nd | nd | nd | nd | nd | nd | nd | nd | nd | nd | nd |
| C2 | FB* | nd | nd | nd | nd | nd | nd | nd | nd | nd | nd | nd | nd | nd | nd | nd | nd | nd | nd | nd |
| C3 | FB* | nd | nd | nd | nd | nd | nd | nd | nd | nd | nd | nd | nd | nd | nd | nd | nd | nd | nd | nd |
| C4 | FB* | nd | nd | nd | nd | nd | nd | nd | nd | nd | nd | nd | nd | nd | nd | nd | nd | nd | nd | nd |
| C5 | FB* | nd | nd | nd | nd | nd | nd | nd | nd | nd | nd | nd | nd | nd | nd | 1.17 | nd | nd | nd | nd |
| C6 | FB* | nd | nd | nd | nd | nd | nd | nd | nd | nd | nd | nd | nd | nd | nd | nd | nd | nd | nd | nd |
| C7 | FB* | nd | nd | nd | nd | nd | nd | nd | nd | nd | nd | nd | nd | nd | nd | nd | nd | nd | nd | nd |
| * Assuming an average dust mass of ~13 mg | | | | | | | | | | | | | | | | | | | | |

**Table S7**: Neutral PFAS measured in dust samples from New York (NY) homes. “nd”: not detected

| **Home ID** | **Size fraction** | **6:2 FTOH** | **8:2 FTOH** | **10:2 FTOH** | **8:2 FTAC** | **10:2 FTAC** | **EtFOSA** | **MeFOSA** | **MeFOSE** | **EtFOSE** |
| --- | --- | --- | --- | --- | --- | --- | --- | --- | --- | --- |
|  |  | **ng/g** | | | | | | | | |
|  | *MDL* | *4.53* | *5.99* | *6.86* | *5.37* | *6.08* | *6.96* | *7.51* | *1.72* | *1.64* |
| C1 | >1180 µm | 3.03 | 1.41 | 1.11 | nd | nd | nd | nd | 1.07 | 1.19 |
| C1 | 600-1180 µm | 25.5 | 13.4 | 6.89 | nd | nd | nd | nd | 2.39 | 2.22 |
| C1 | <600 µm* | 14.8 | 7.64 | 6.20 | nd | nd | 0.512 | nd | 5.10 | 5.16 |
| C1 | 250-600 µm | 8.31 | 5.56 | 4.55 | nd | nd | nd | nd | 4.02 | 4.20 |
| C1 | 150-250 µm | 15.9 | 8.06 | 7.77 | nd | nd | nd | nd | 5.22 | 5.12 |
| C1 | 75-150 µm | 21. 6 | 9.31 | 6.11 | nd | nd | 1.79 | nd | 5.07 | 5.12 |
| C1 | <75 µm | 19.7 | 10.8 | 10.1 | nd | nd | nd | nd | 9.96 | 10.0 |
| C2 | >1180 µm | 233 | 18.1 | 9.49 | nd | nd | nd | nd | 2.58 | 1.98 |
| C2 | 600-1180 µm | 71.2 | 39.5 | 23.21 | nd | nd | nd | nd | 4.74 | 3.67 |
| C2 | <600 µm* | 255 | 214 | 78.5 | nd | nd | nd | nd | 6.39 | 7.53 |
| C2 | 250-600 µm | 82.0 | 20.2 | 12.3 | nd | nd | nd | nd | 4.14 | 3.63 |
| C2 | 150-250 µm | 290 | 349 | 82.8 | nd | nd | nd | nd | 4.93 | 7.84 |
| C2 | 75-150 µm | 475 | 315 | 160 | nd | nd | nd | nd | 8.72 | 11.3 |
| C2 | <75 µm | 576 | 418 | 231 | nd | nd | nd | nd | 17.09 | 17.3 |
| C3 | >1180 µm | 2.95 | 2.93 | 1.77 | nd | nd | nd | nd | 1.21 | 1.58 |
| C3 | 600-1180 µm | 135 | 1.13 | 1.05 | nd | nd | 0.04 | nd | 2.12 | 2.01 |
| C3 | <600 µm* | 56.2 | 11.9 | 4.87 | nd | nd | nd | nd | 3.59 | 9.23 |
| C3 | 250-600 µm | 34.0 | 4.70 | 2.54 | nd | nd | nd | nd | 2.04 | 4.36 |
| C3 | 150-250 µm | 86.4 | 29.2 | 7.32 | nd | nd | nd | nd | 4.08 | 9.11 |
| C3 | 75-150 µm | 74.4 | 9.51 | 5.74 | nd | nd | nd | nd | 3.76 | 7.74 |
| C3 | <75 µm | 95.8 | 24.8 | 14.7 | nd | nd | nd | nd | 15.3 | 58.3 |
| C4 | >1180 µm | 28.2 | 1.72 | 1.25 | nd | nd | 0.66 | nd | 1.68 | 1.88 |
| C4 | 600-1180 µm | 8.93 | 1.02 | 0.98 | nd | nd | 0.53 | nd | 1.12 | 0.91 |
| C4 | <600 µm* | 21.3 | 7.56 | 5.10 | nd | nd | 0.866 | nd | 3.88 | 4.31 |
| C4 | 250-600 µm | 15.1 | 5.52 | 3.88 | nd | nd | 0.92 | nd | 2.52 | 2.18 |
| C4 | 150-250 µm | 23.6 | 9.68 | 7.01 | nd | nd | 1.67 | nd | 5.23 | 5.35 |
| C4 | 75-150 µm | 33.00 | 8.97 | 4.77 | nd | nd | nd | nd | 4.04 | 6.45 |
| C4 | <75 µm | 37.0 | 15.5 | 11.2 | nd | nd | nd | nd | 11.9 | 14.9 |
| C5 | >1180 µm | 8.93 | 3.36 | 2.23 | nd | nd | 1.13 | nd | 2.49 | 1.68 |
| C5 | 600-1180 µm | 44.8 | 5.49 | 3.14 | nd | nd | 1.43 | nd | 3.02 | 2.31 |
| C5 | <600 µm* | 25.4 | 13.2 | 7.16 | nd | nd | 0.360 | nd | 3.77 | 3.54 |
| C5 | 250-600 µm | 6.99 | 4.50 | 2.26 | nd | nd | 0.51 | nd | 1.70 | 1.31 |
| C5 | 150-250 µm | 25.7 | 16.0 | 8.16 | nd | nd | nd | nd | 4.01 | 3.66 |
| C5 | 75-150 µm | 51.7 | 24.7 | 12.2 | nd | nd | 0.50 | nd | 4.94 | 4.50 |
| C5 | <75 µm | 41.4 | 17.0 | 14.8 | nd | nd | nd | nd | 10.2 | 11.6 |
| C6 | >1180 µm | 25.8 | 11.0 | 7.58 | nd | nd | 2.46 | nd | 2.98 | 3.57 |
| C6 | 600-1180 µm | 45.4 | 26.5 | 16.0 | nd | nd | 2.85 | nd | 5.11 | 3.66 |
| C6 | <600 µm* | 64.3 | 96.0 | 41.4 | nd | nd | 1.00 | nd | 4.99 | 4.23 |
| C6 | 250-600 µm | 49.9 | 99.1 | 33.2 | nd | nd | 1.07 | nd | 3.02 | 2.77 |
| C6 | 150-250 µm | 33.5 | 73.9 | 31.3 | nd | nd | nd | nd | 5.24 | 4.25 |
| C6 | 75-150 µm | 146 | 130 | 77.7 | nd | nd | 2.48 | nd | 7.27 | 5.83 |
| C6 | <75 µm | 64.7 | 49.7 | 38.9 | nd | nd | nd | nd | 16.8 | 14.5 |
| C7 | >1180 µm | 30.4 | 46.6 | nd | nd | nd | nd | nd | 7.70 | 7.01 |
| C7 | 600-1180 µm | 14.8 | 15.0 | 12.1 | nd | nd | 0.15 | nd | 5.88 | 5.09 |
| C7 | <600 µm* | 53.9 | 37.4 | 24.0 | nd | nd | 2.02 | nd | 9.22 | 8.15 |
| C7 | 250-600 µm | 41.2 | 20.6 | 11.0 | nd | nd | 0.87 | nd | 4.14 | 3.76 |
| C7 | 150-250 µm | 27.2 | 20.0 | 15.8 | nd | nd | 0.71 | nd | 7.41 | 6.06 |
| C7 | 75-150 µm | 92.3 | 70.7 | 39.0 | 16.7 | 17.4 | 3.10 | nd | 12.3 | 11.4 |
| C7 | <75 µm | 137 | 122 | 100 | 72.2 | 78.2 | 12.1 | nd | 41.2 | 36.5 |
| C1 | FB** | nd | nd | nd | nd | nd | nd | nd | nd | nd |
| C2 | FB** | nd | nd | nd | nd | nd | nd | nd | nd | nd |
| C3 | FB** | nd | nd | nd | nd | nd | nd | nd | nd | nd |
| C4 | FB** | 1.64 | 1.82 | 2.46 | nd | nd | nd | nd | nd | nd |
| C5 | FB** | 0.353 | 1.07 | nd | nd | nd | nd | nd | nd | nd |
| C6 | FB** | 2.61 | 1.72 | nd | nd | nd | nd | nd | nd | nd |
| C7 | FB** | nd | nd | nd | nd | nd | nd | nd | nd | nd |
| * Weighted sum  ** Assuming an average dust mass of 135 mg | | | | | | | | | | |

**
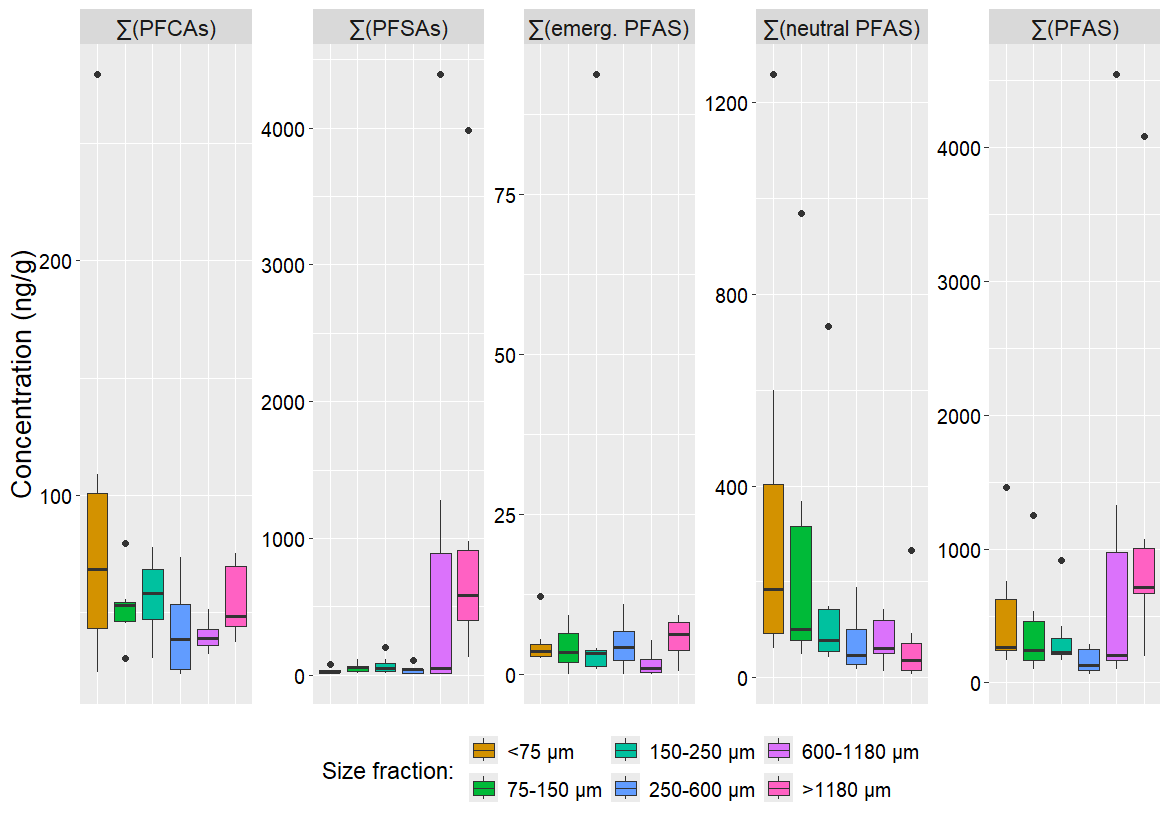
**

**Figure S3**: Box-and-whisker plots of ∑(PFCAs), ∑(PFSAs), ∑(emerging PFAS), ∑(neutral PFAS) and ∑(PFAS) found in six different size fractions in NY homes.


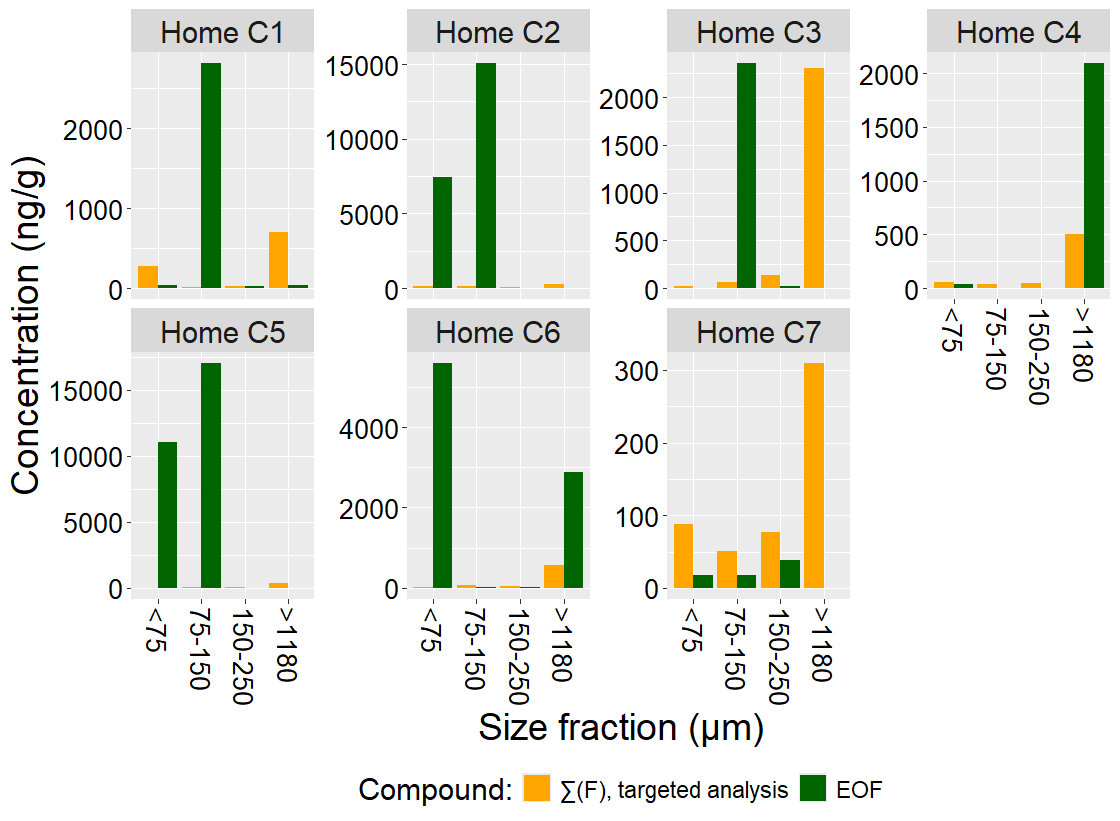


**Figure S4**: Extractable organic fluorine (EOF, green) and sum of measured ionic/ionizable PFAS as fluorine (∑(F); orange) concentrations in NY house dust samples (home IDs C1-C7). Note that all EOF values shown are below the MDL of 20,000 ng/g.

**Table S8**: PFAS measured in dust samples and field blanks (FBs) from North Carolina (NC) homes using the UNC-CH method for ionic/ionizable PFAS (LC-MS/MS). Calculated concentrations in the <500 µm size fractions have been reported in Chang et al. (2025). “nd”: not detected

| **Home ID** | **Size fraction** | **PFBA** | **PFPeA** | **PFHxA** | **PFHpA** | **PFOA** | **PFNA** | **PFDA** | **PFuDA** | **PFDoA** | **PFTrDA** | **PFTeDA** | **PFHxDA** | **PFODA** |
| --- | --- | --- | --- | --- | --- | --- | --- | --- | --- | --- | --- | --- | --- | --- |
|  |  | **ng/g** | | | | | | | | | | | | |
|  | *MDL* | *0.03* | *0.02* | *0.02* | *0.03* | *0.04* | *0.03* | *0.03* | *0.09* | *0.48* | *0.31* | *0.73* | *0.53* | *0.04* |
| 01 | 250-500 µm | 5.59 | nd | 2.22 | nd | 2.71 | nd | 0.32 | nd | nd | nd | nd | nd | nd |
| 01 | 125-250 µm | 3.48 | 0.26 | 1.17 | 0.13 | 2.22 | nd | 0.14 | nd | nd | nd | nd | nd | nd |
| 01 | 63-125 µm | 2.16 | nd | 0.85 | 0.16 | 1.72 | nd | 0.20 | nd | nd | nd | nd | nd | nd |
| 01 | <63 µm | 3.73 | nd | 1.90 | 0.43 | 3.89 | nd | 0.44 | nd | nd | nd | nd | nd | nd |
| 10 | 250-500 µm | 1.65 | 0.63 | 3.79 | 0.74 | 3.87 | nd | nd | nd | nd | nd | nd | nd | nd |
| 10 | <250 µm | 2.79 | 0.70 | 4.38 | 2.86 | 4.45 | nd | 0.10 | nd | nd | nd | nd | nd | nd |
| 18 | 250-500 µm | 125 | 1.63 | 7.27 | 4.51 | 14.5 | nd | 0.06 | nd | nd | nd | nd | nd | nd |
| 18 | <250 µm | 159 | 1.97 | 9.92 | 5.22 | 16.8 | nd | 0.30 | nd | nd | nd | nd | nd | nd |
| 30 | 250-500 µm | 1.78 | 0.25 | 0.94 | 0.11 | 1.46 | nd | nd | nd | nd | nd | nd | nd | nd |
| 30 | <250 µm | 1.94 | nd | 1.55 | 0.13 | 1.26 | nd | nd | nd | nd | nd | nd | nd | nd |
| 35 | 250-500 µm | 0.37 | nd | nd | nd | nd | nd | nd | nd | nd | nd | nd | nd | nd |
| 35 | <250 µm | 3.38 | nd | 1.56 | 0.08 | 0.97 | nd | nd | nd | nd | nd | nd | nd | nd |
| 43 | 250-500 µm | 1.51 | nd | 0.04 | nd | 0.39 | nd | 0.14 | nd | nd | nd | nd | nd | nd |
| 43 | 125-250 µm | 0.36 | nd | 0.90 | nd | 0.81 | nd | nd | nd | nd | nd | nd | nd | nd |
| 43 | 63-125 µm | 2.85 | nd | 4.41 | 1.98 | 2.81 | nd | 1.12 | nd | nd | nd | nd | nd | nd |
| 43 | <63 µm | 2.54 | nd | 3.83 | 1.38 | 4.30 | nd | 1.30 | nd | nd | nd | nd | nd | nd |
| 50 | 250-500 µm | 3.28 | 0.36 | 0.29 | nd | 0.95 | nd | nd | nd | nd | nd | nd | nd | nd |
| 50 | <250 µm | 5.97 | 0.65 | 7.84 | 4.28 | 8.23 | nd | 0.78 | nd | nd | nd | nd | nd | nd |
| 59 | 250-500 µm | 3.31 | 0.68 | nd | nd | 3.86 | nd | 0.16 | nd | nd | nd | nd | nd | nd |
| 59 | <250 µm | 7.93 | nd | 0.85 | 1.47 | 5.40 | nd | 2.04 | nd | nd | nd | nd | nd | nd |
| 65 | 250-500 µm | 6.45 | 0.59 | 5.80 | 1.17 | 4.31 | nd | 0.50 | nd | nd | nd | nd | nd | nd |
| 65 | <250 µm | 8.46 | 0.98 | 10.6 | 1.67 | 5.45 | nd | 2.02 | nd | nd | nd | nd | nd | nd |
| 78 | 250-500 µm | nd | 0.68 | nd | nd | nd | nd | nd | nd | nd | nd | nd | nd | nd |
| 78 | <250 µm | 4.01 | 0.37 | 0.55 | nd | nd | nd | nd | nd | nd | nd | nd | nd | nd |
| 01 | FB* | nd | nd | nd | nd | nd | nd | nd | nd | nd | nd | nd | nd | nd |
| 18 | FB* | nd | 0.42 | nd | nd | nd | nd | nd | nd | nd | nd | nd | nd | nd |
| 30 | FB* | nd | nd | nd | nd | nd | nd | nd | nd | nd | nd | nd | nd | nd |
| 35 | FB* | nd | nd | nd | nd | nd | nd | nd | nd | nd | nd | nd | nd | nd |
| 50 | FB* | nd | nd | nd | nd | nd | nd | nd | nd | nd | nd | nd | nd | nd |
| 59 | FB* | nd | nd | nd | nd | nd | nd | nd | nd | nd | nd | nd | nd | nd |
| 65 | FB* | nd | nd | nd | nd | nd | nd | nd | nd | nd | nd | nd | nd | nd |
| 78 | FB* | nd | nd | nd | nd | nd | nd | nd | nd | nd | nd | nd | nd | nd |

| **Home ID** | **Size fraction** | **PFBS** | **PFPeS** | **L-PFHxS** | **PFHpS** | **L-PFOS** | **PFNS** | **PFDS** | **PFDoS** | **GenX** | **6:2 monoPAP** | **8:2 monoPAP** | **6:2 diPAP** | **8:2 diPAP** |
| --- | --- | --- | --- | --- | --- | --- | --- | --- | --- | --- | --- | --- | --- | --- |
|  |  | **ng/g** | | | | | | | | | | | | |
|  | *MDL* | *0.10* | *0.06* | *0.06* | *0.03* | *0.04* | *0.04* | *0.13* | *0.46* | *0.39* | *0.44* | *0.33* | *.73* | *0.31* |
| 01 | 250-500 µm | nd | nd | nd | nd | 173 | nd | nd | nd | nd | nd | nd | 51.5 | 6.6 |
| 01 | 125-250 µm | 0.81 | nd | nd | nd | 143 | nd | nd | nd | nd | 8.37 | 4.18 | 45.5 | 2.7 |
| 01 | 63-125 µm | 0.85 | nd | nd | nd | 28.1 | nd | nd | nd | nd | nd | nd | 10.6 | 1.5 |
| 01 | <63 µm | 1.44 | nd | nd | nd | 59.9 | nd | nd | nd | nd | nd | nd | 28.4 | 2.7 |
| 10 | 250-500 µm | 1.14 | nd | nd | nd | 17.5 | nd | nd | nd | nd | nd | nd | 179 | 53.3 |
| 10 | <250 µm | 1.05 | nd | nd | nd | 20.4 | nd | nd | nd | nd | 22.96 | nd | 161 | 60.0 |
| 18 | 250-500 µm | 11.6 | nd | nd | nd | 78.4 | nd | nd | nd | nd | 2.16 | nd | 169 | 11.9 |
| 18 | <250 µm | 14.3 | nd | nd | nd | 81.7 | nd | nd | nd | nd | 29.78 | nd | 165 | 14.5 |
| 30 | 250-500 µm | nd | nd | nd | nd | 15.1 | nd | nd | nd | nd | 37.29 | nd | 325 | 6.5 |
| 30 | <250 µm | 1.22 | nd | nd | nd | 19.8 | nd | nd | nd | nd | 163.30 | nd | 410 | 2.8 |
| 35 | 250-500 µm | nd | nd | nd | nd | 19.1 | nd | nd | nd | nd | nd | nd | 53.9 | 1.0 |
| 35 | <250 µm | nd | nd | nd | nd | 22.3 | nd | nd | nd | nd | nd | nd | 189 | 3.0 |
| 43 | 250-500 µm | 3.66 | nd | nd | nd | 53.8 | nd | nd | nd | nd | nd | nd | 166 | 3.5 |
| 43 | 125-250 µm | 1.51 | nd | nd | nd | 48.5 | nd | nd | nd | nd | nd | nd | 119 | 3.3 |
| 43 | 63-125 µm | 1.53 | nd | 11.7 | nd | 71.9 | nd | nd | nd | nd | 5.71 | 8.04 | 151 | 4.1 |
| 43 | <63 µm | nd | nd | 8.20 | nd | 54.8 | nd | nd | nd | nd | nd | nd | 206 | 5.7 |
| 50 | 250-500 µm | 1.82 | nd | nd | nd | 46.7 | nd | nd | nd | nd | nd | nd | 25.2 | 2.4 |
| 50 | <250 µm | 3.48 | nd | nd | nd | 152 | nd | nd | nd | nd | nd | nd | 128 | 25.8 |
| 59 | 250-500 µm | nd | nd | nd | nd | 34.8 | nd | nd | nd | nd | nd | nd | 25.7 | 7.4 |
| 59 | <250 µm | 2.20 | nd | nd | nd | 115 | nd | nd | nd | nd | nd | nd | 63.1 | 15.6 |
| 65 | 250-500 µm | 4.77 | nd | nd | nd | 69.2 | 1.65 | 8.82 | nd | nd | nd | nd | 69.7 | 56.7 |
| 65 | <250 µm | 1.08 | nd | nd | nd | 30.4 | nd | nd | nd | nd | nd | nd | 80.6 | 50.0 |
| 78 | 250-500 µm | 1.88 | nd | nd | nd | 146 | nd | nd | nd | nd | nd | nd | 62.8 | 1.3 |
| 78 | <250 µm | nd | nd | nd | nd | 227 | nd | nd | nd | nd | nd | nd | 132 | 2.8 |
| 01 | FB* | 3.57 | nd | nd | nd | 6.23 | nd | nd | nd | nd | nd | nd | nd | 1.34 |
| 18 | FB* | 3.36 | nd | nd | nd | 0.96 | nd | nd | nd | nd | nd | nd | nd | 2.34 |
| 30 | FB* | 4.09 | nd | nd | nd | 0.66 | nd | nd | nd | nd | nd | nd | nd | 1.71 |
| 35 | FB* | 4.09 | nd | nd | nd | nd | nd | nd | nd | nd | nd | nd | nd | 0.81 |
| 50 | FB* | 3.86 | nd | nd | nd | 0.29 | nd | nd | nd | nd | nd | nd | nd | 1.18 |
| 59 | FB* | 4.35 | nd | nd | nd | 0.60 | nd | nd | nd | nd | nd | nd | nd | 0.65 |
| 65 | FB* | 1.60 | nd | nd | nd | 0.60 | nd | nd | nd | nd | nd | nd | nd | 3.62 |
| 78 | FB* | 2.10 | nd | nd | nd | 0.45 | nd | nd | nd | nd | nd | nd | nd | 1.76 |
| * Assuming an average dust mass of 138 mg | | | | | | | | | | | | | | |
|  | | | | | | | | | | | | | | |

**Table S9**: Neutral PFAS measured in dust samples and field blanks (FBs) from North Carolina (NC) homes. Calculated concentrations in the <500 µm fractions have been reported in Eichler et al. (2024). “nd”: not detected

| **Home ID** | **Size fraction** | **6:2 FTOH** | **8:2 FTOH** | **10:2 FTOH** | **8:2 FTAC** | **10:2 FTAC** | **EtFOSA** | **MeFOSA** | **MeFOSE** | **EtFOSE** |
| --- | --- | --- | --- | --- | --- | --- | --- | --- | --- | --- |
|  |  | **ng/g** | | | | | | | | |
|  | *MDL* | *4.53* | *5.99* | *6.86* | *5.37* | *6.08* | *6.96* | *7.51* | *1.72* | *1.64* |
| 01 | 250-500 µm | 24.4 | 10.6 | 7.96 | nd | nd | nd | nd | 12.4 | 17.0 |
| 01 | 125-250 µm | 48.1 | 19.1 | 10.1 | nd | nd | nd | nd | 8.94 | 46.3 |
| 01 | 63-125 µm | 36.3 | 20.9 | 12.7 | nd | nd | nd | nd | 12.2 | 56.0 |
| 01 | <63 µm | 65.2 | 26.4 | 16.6 | nd | nd | nd | nd | 15.5 | 70.9 |
| 10 | 250-500 µm | 46.1 | 25.4 | 23.5 | nd | nd | nd | nd | 1.38 | 5.99 |
| 10 | <250 µm | 88.2 | 65.3 | 50.5 | nd | nd | nd | nd | 1.76 | 11.1 |
| 18 | 250-500 µm | 118 | 24.4 | 17.2 | 1.00 | nd | 0.18 | nd | 29.8 | 127 |
| 18 | <250 µm | 206 | 29.1 | 22.6 | 1.14 | nd | 0.39 | nd | 48.7 | 217 |
| 30 | 250-500 µm | 87.1 | 13.9 | 15.0 | nd | nd | nd | nd | 3.46 | 1.16 |
| 30 | <250 µm | 358 | 24.8 | 13.9 | nd | nd | nd | nd | 11.8 | 5.66 |
| 35 | 250-500 µm | 27.2 | 8.77 | 9.21 | nd | nd | nd | nd | 0.35 | 0.55 |
| 35 | <250 µm | 30.8 | 30.4 | 19.5 | nd | nd | nd | nd | 0.74 | 2.37 |
| 43 | 250-500 µm | 20.1 | 64.6 | 8.15 | 0.25 | 0.20 | 0.09 | nd | 1.63 | 2.01 |
| 43 | 125-250 µm | 28.3 | 113 | 13.2 | 0.14 | 0.06 | 0.26 | nd | 3.14 | 4.80 |
| 43 | 63-125 µm | 40.6 | 101 | 19.8 | 0.13 | 0.07 | 0.55 | nd | 4.73 | 10.5 |
| 43 | <63 µm | 55.1 | 85.6 | 30.6 | 0.10 | 0.03 | 0.12 | nd | 4.68 | 7.64 |
| 50 | 250-500 µm | 61.7 | 8.51 | 8.15 | nd | nd | nd | nd | 44.4 | 13.6 |
| 50 | <250 µm | 139 | 24.6 | 19.4 | nd | nd | nd | nd | 49.5 | 21.1 |
| 59 | 250-500 µm | 46.8 | 24.7 | 21.5 | nd | nd | nd | nd | 2.99 | 459 |
| 59 | <250 µm | 56.6 | 44.2 | 34.0 | nd | nd | nd | nd | 5.63 | 18.4 |
| 65 | 250-500 µm | 24.9 | 21.9 | 18.0 | nd | nd | nd | nd | 3.51 | 2.24 |
| 65 | <250 µm | 42.7 | 33.1 | 24.7 | nd | nd | nd | nd | 8.65 | 14.8 |
| 78 | 250-500 µm | 76.6 | 40.7 | 42.7 | nd | nd | 0.12 | nd | 2.87 | 3.12 |
| 78 | <250 µm | 262 | 159 | 151 | nd | nd | 0.14 | nd | 2.29 | 3.47 |
| 01 | FB* | nd | nd | nd | nd | nd | nd | nd | nd | nd |
| 10 | FB* | 3.20 | nd | nd | nd | nd | nd | nd | nd | nd |
| 43 | FB* | 3.31 | nd | nd | nd | nd | 0.67 | nd | nd | nd |
| 50 | FB* | nd | nd | nd | nd | nd | 0.48 | nd | nd | nd |
| * Assuming an average dust mass of 106 mg | | | | | | | | | | |


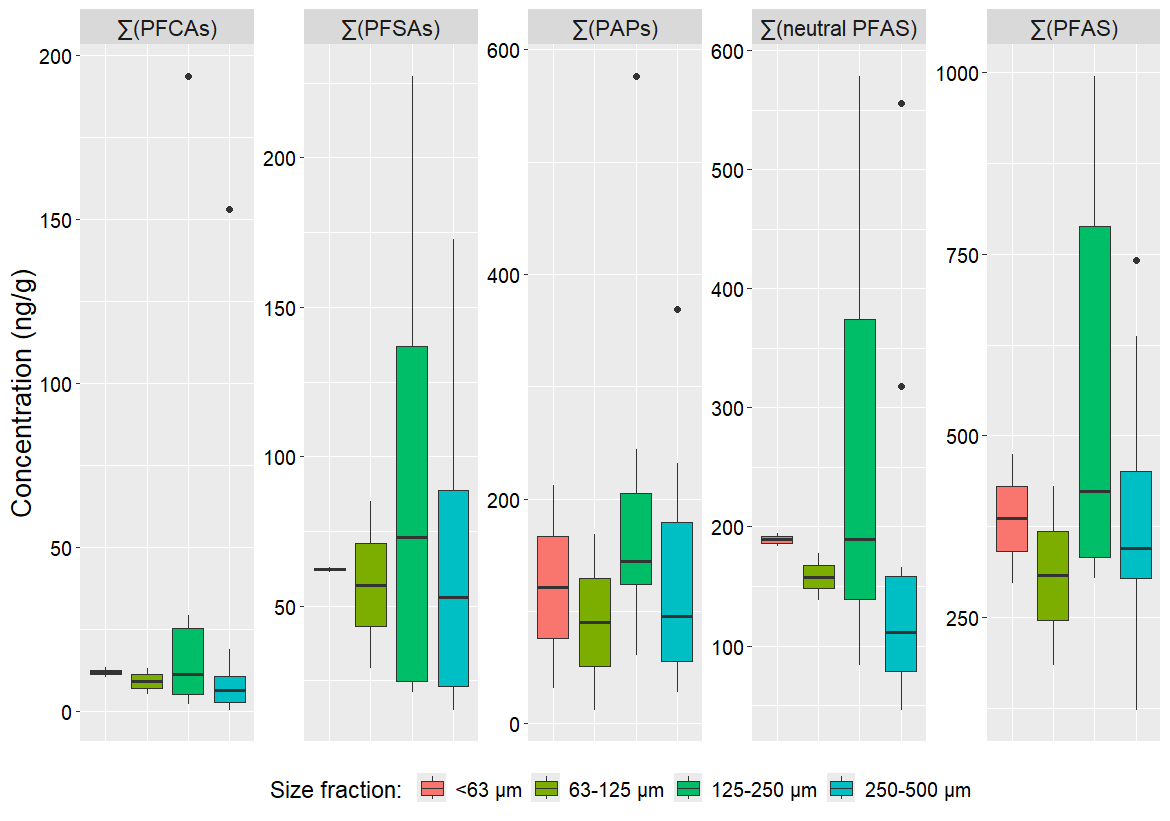


**Figure S5**: Box-and-whisker plots of ∑(PFCAs), ∑(PFSAs), ∑(PAPs), ∑(neutral PFAS) and ∑(PFAS) found in four different size fractions in NC homes.


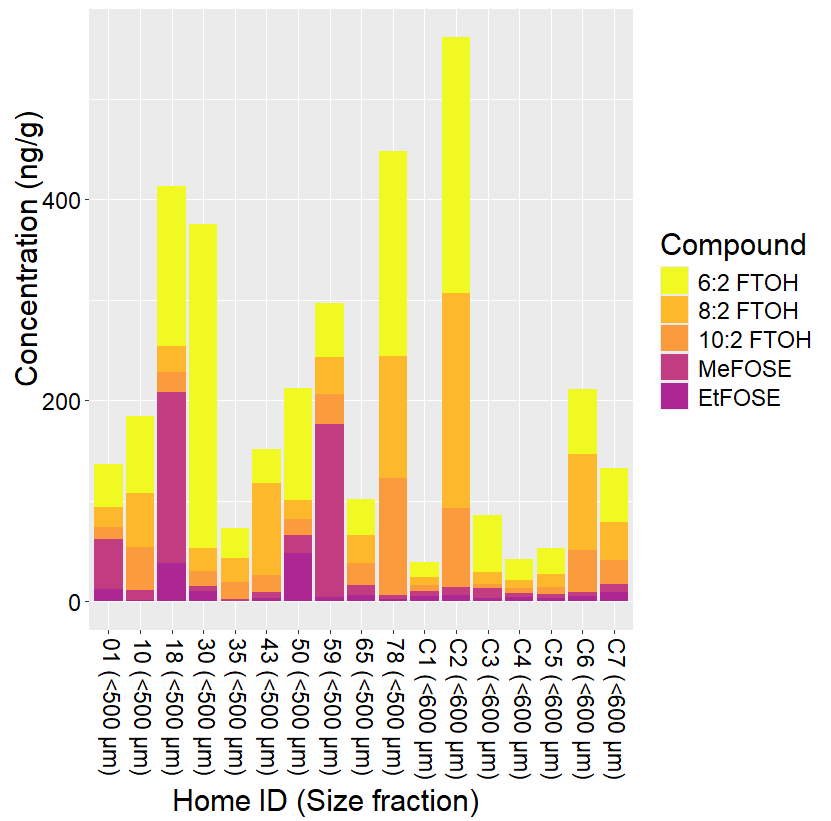


**Figure S6**: Concentrations of neutral PFAS measured with >50% DF above MDL in North Carolina (homes 01-78) and New York (homes C1-C7), weighted sums.

**A**


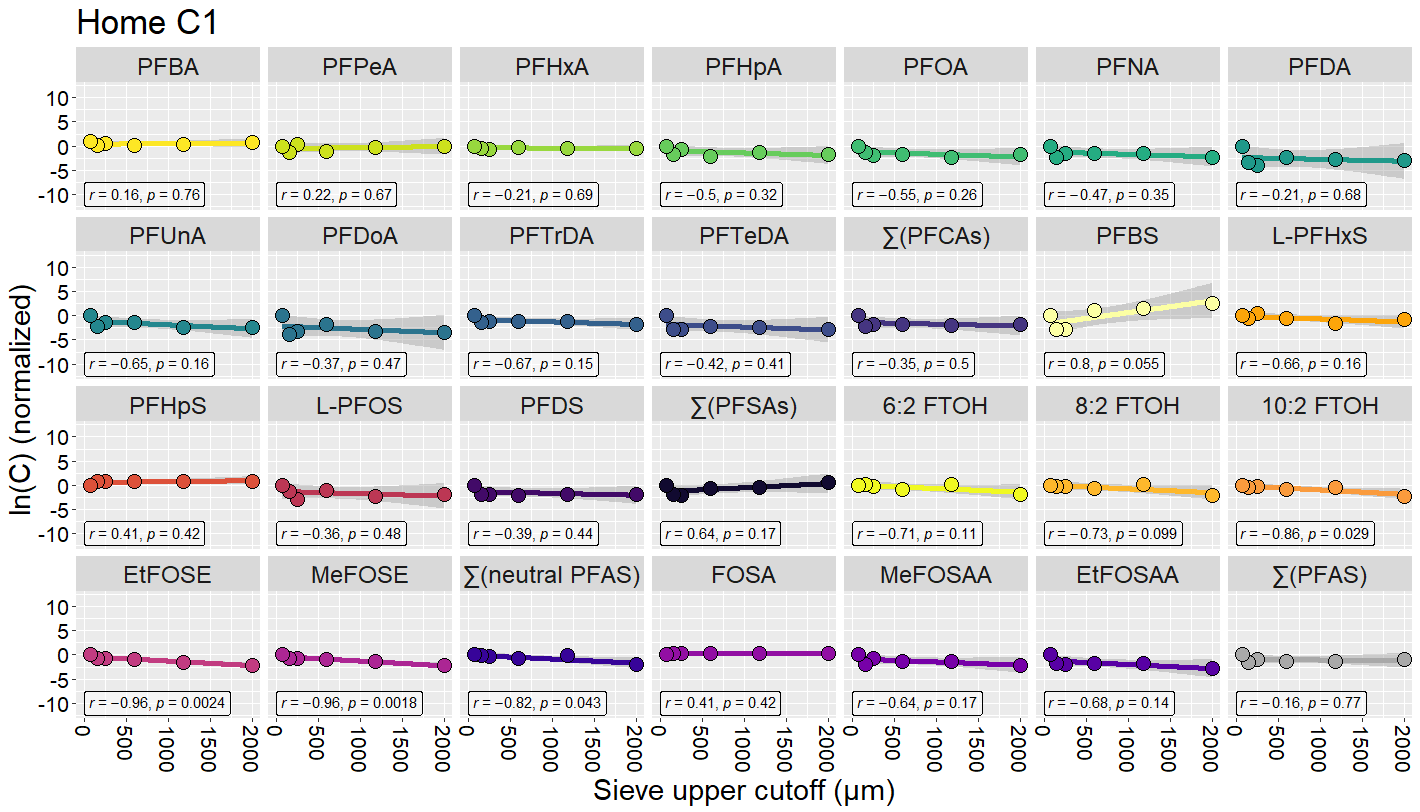


**B**


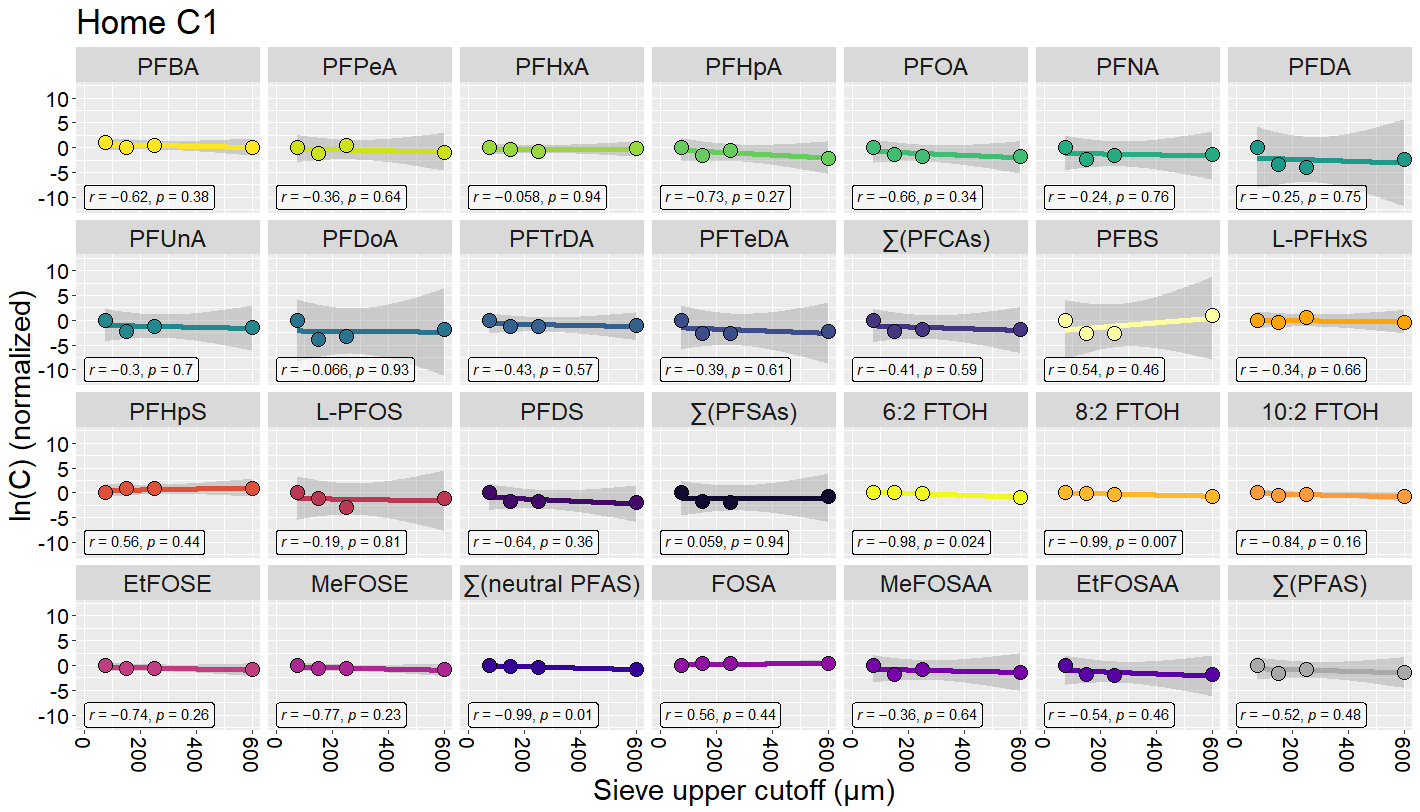


**Figure S7**: Correlations between normalized PFAS concentrations and size fraction found for Home C1 (New York). Concentrations were normalized by the concentrations measured in the 75 µm size fractions. A) Full range of size fractions (<63 µm to <2,000 µm); B) subset of size fractions (<63 µm to <600 µm).

**A**


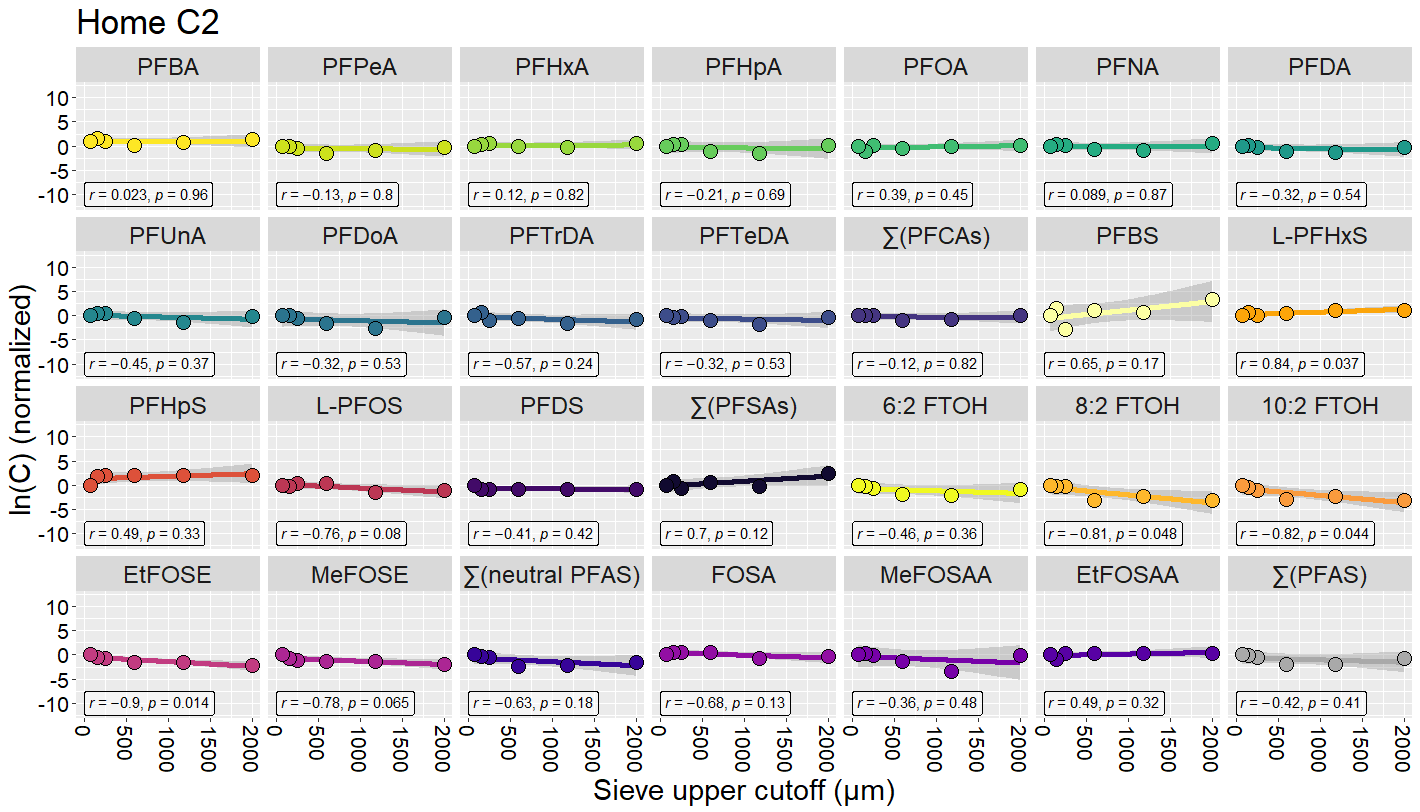


**B**


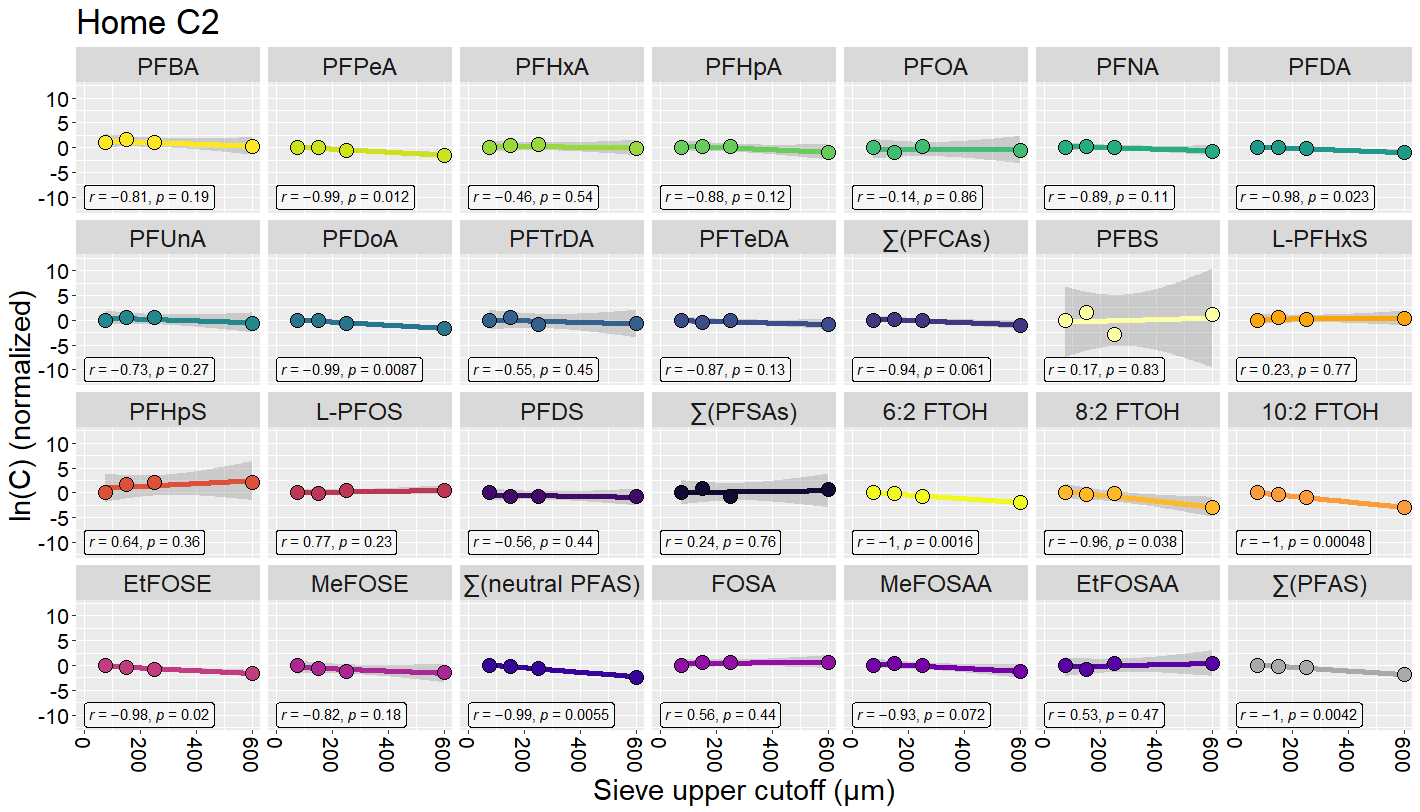


**Figure S8**: Correlations between normalized PFAS concentrations and size fraction found for Home C2 (New York). Concentrations were normalized by the concentrations measured in the 75 µm size fractions. A) Full range of size fractions (<63 µm to <2,000 µm); B) subset of size fractions (<63 µm to <600 µm).

**A**


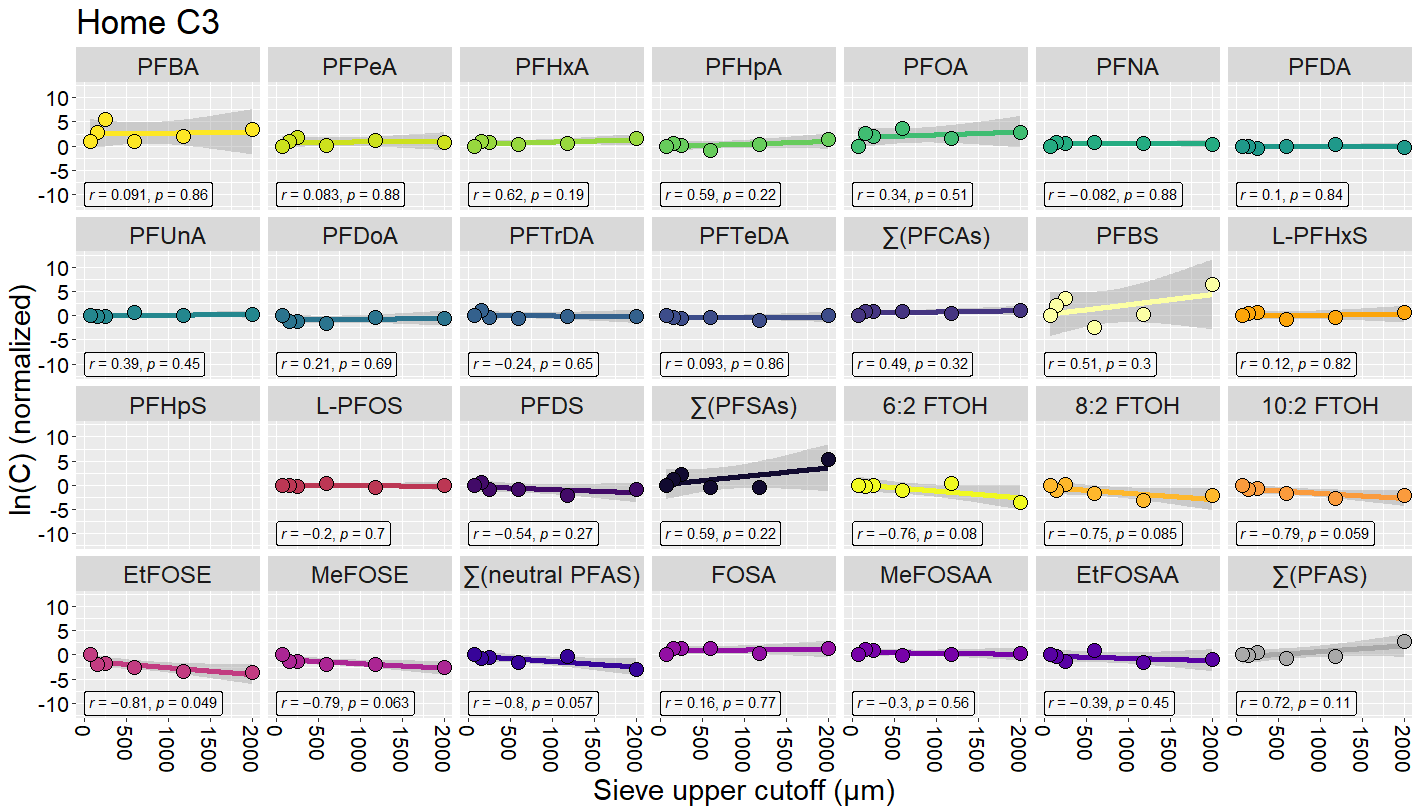


**B**


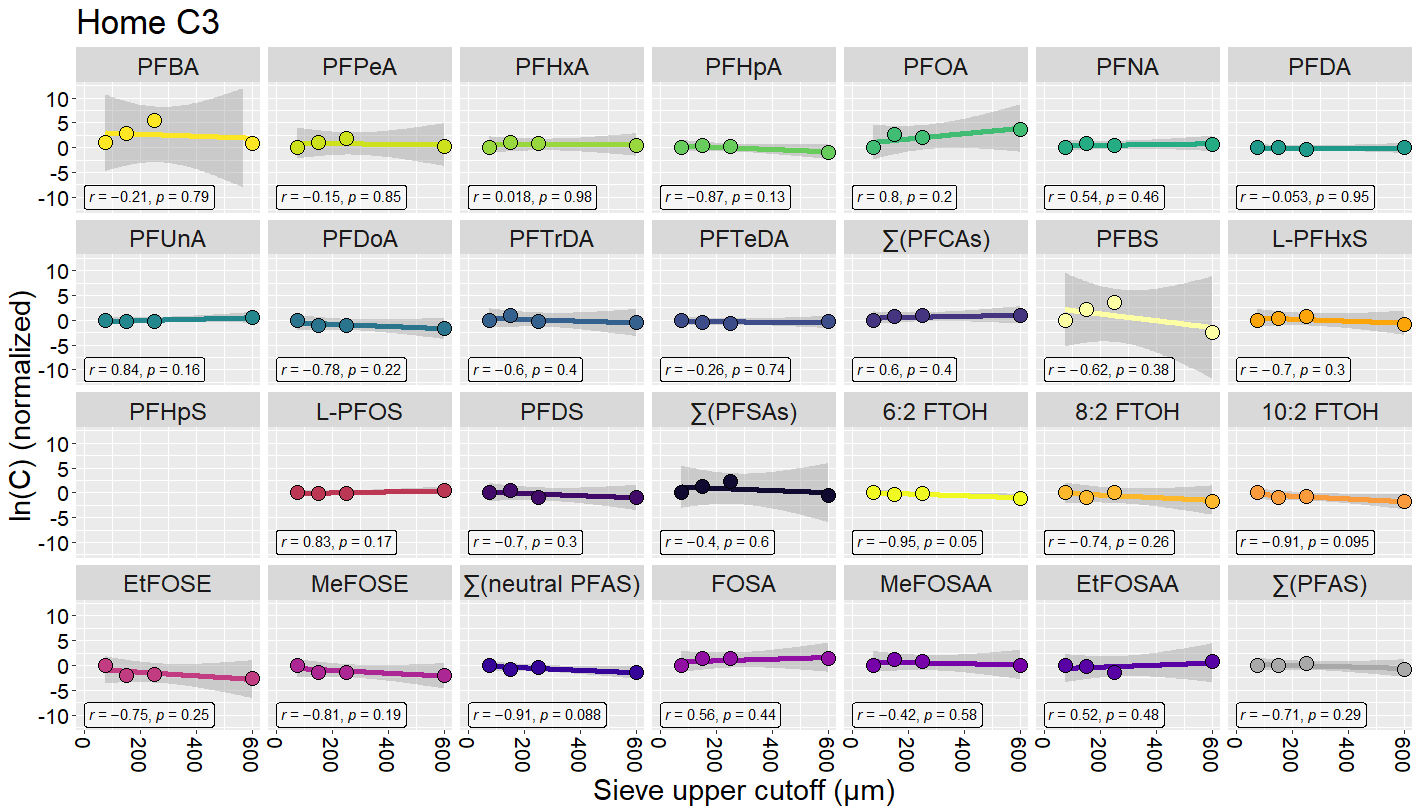


**Figure S9**: Correlations between normalized PFAS concentrations and size fraction found for Home C3 (New York). Concentrations were normalized by the concentrations measured in the 75 µm size fractions. PFHpS was not detected in enough size fractions from Home C3. A) Full range of size fractions (<63 µm to <2,000 µm); B) subset of size fractions (<63 µm to <600 µm).

**A**


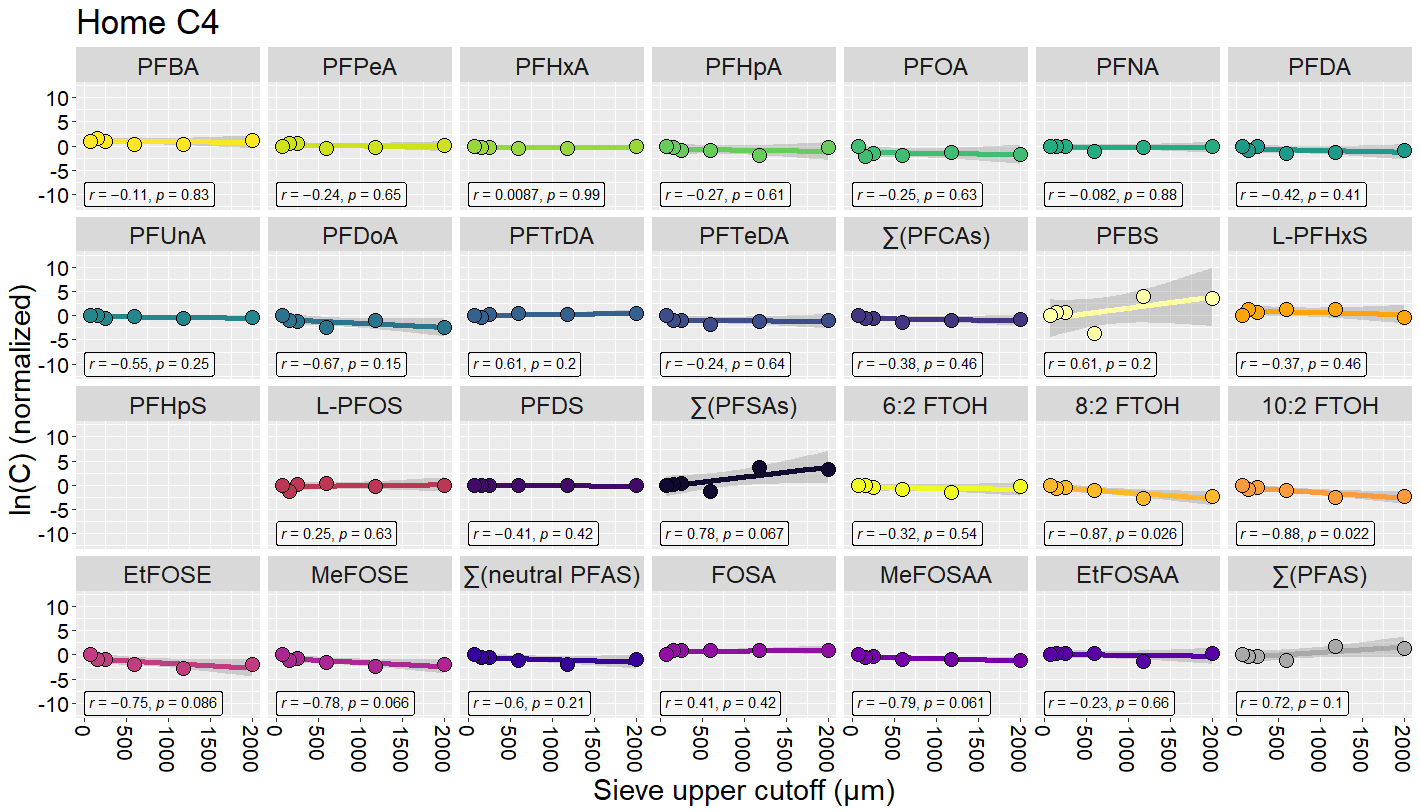


**B**


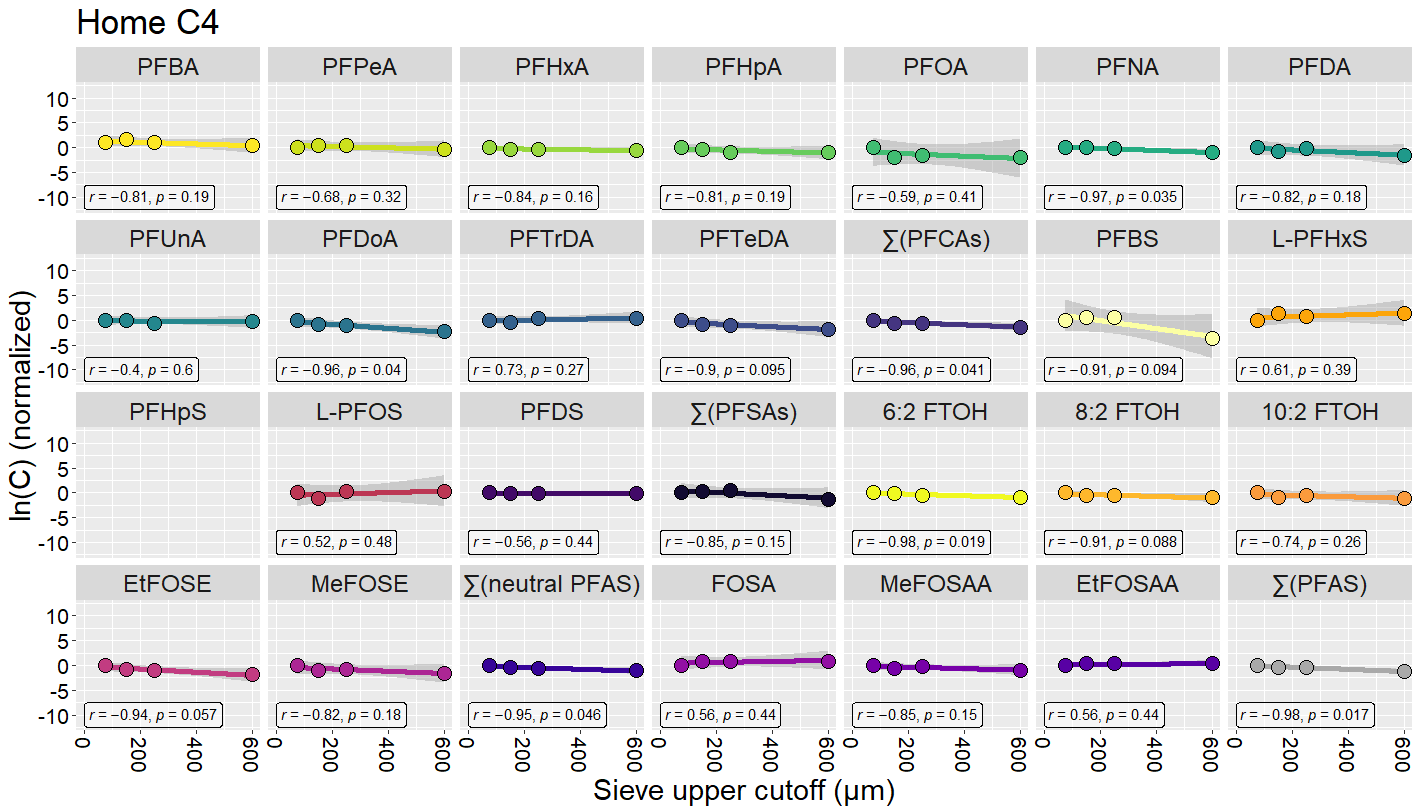


**Figure S10**: Correlations between normalized PFAS concentrations and size fraction found for Home C4 (New York). Concentrations were normalized by the concentrations measured in the 75 µm size fractions. PFHpS was not detected in dust samples from Home C4. A) Full range of size fractions (<63 µm to <2,000 µm); B) subset of size fractions (<63 µm to <600 µm).

**A**


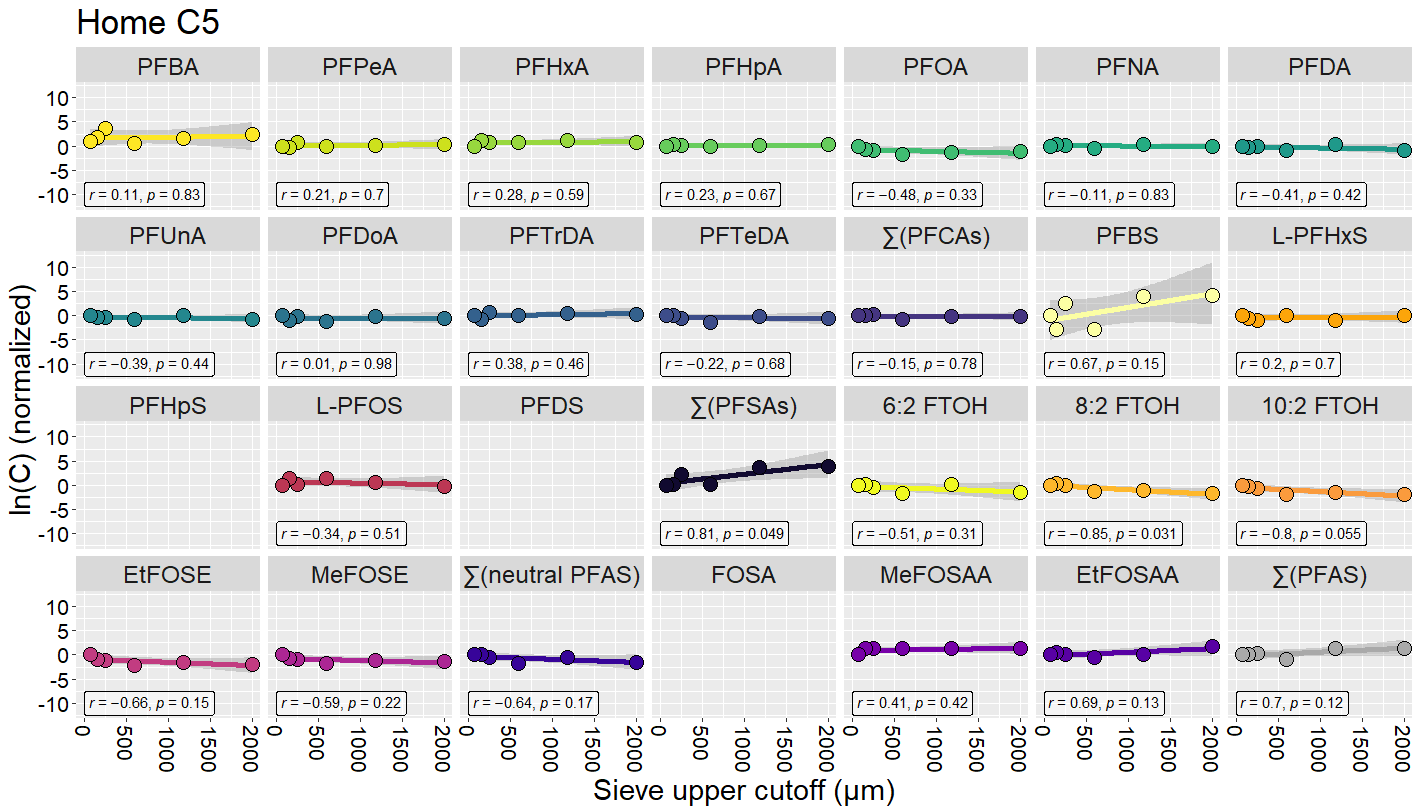


**B**


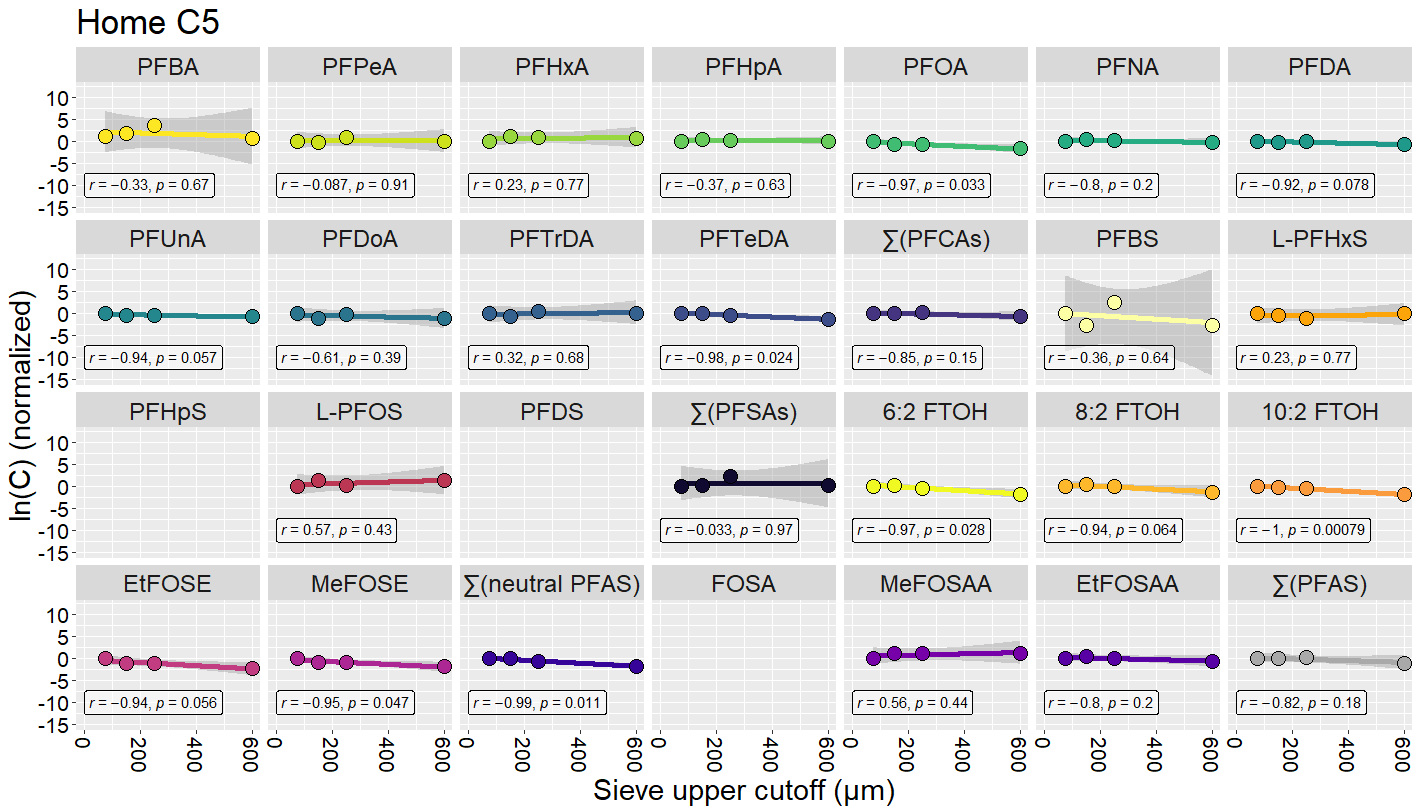


**Figure S11**: Correlations between normalized PFAS concentrations and size fraction found for Home C5 (New York). Concentrations were normalized by the concentrations measured in the 75 µm size fractions. PFHpS, PFDS, and FOSA were not detected in Home C5. A) Full range of size fractions (<63 µm to <2,000 µm); B) subset of size fractions (<63 µm to <600 µm).

**A**


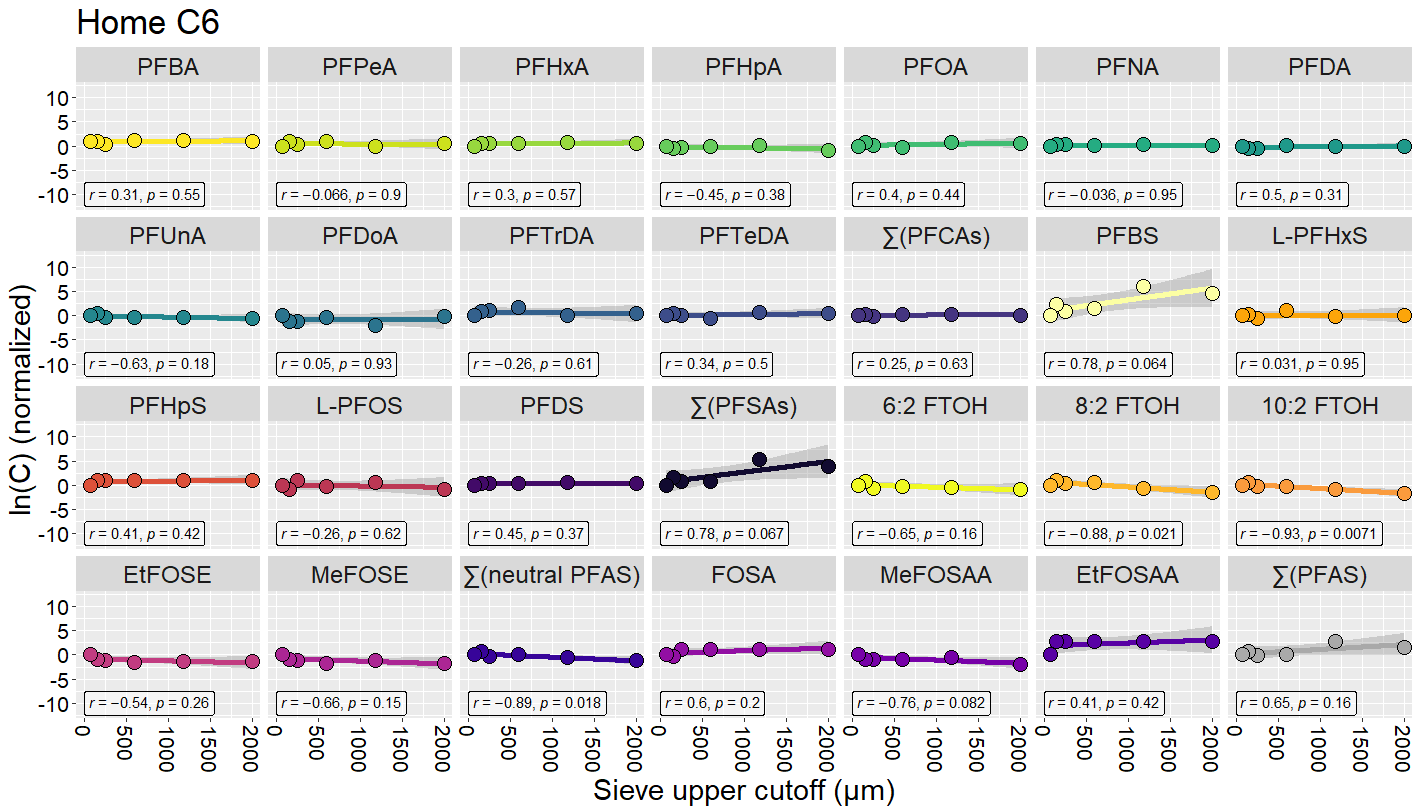


**B**


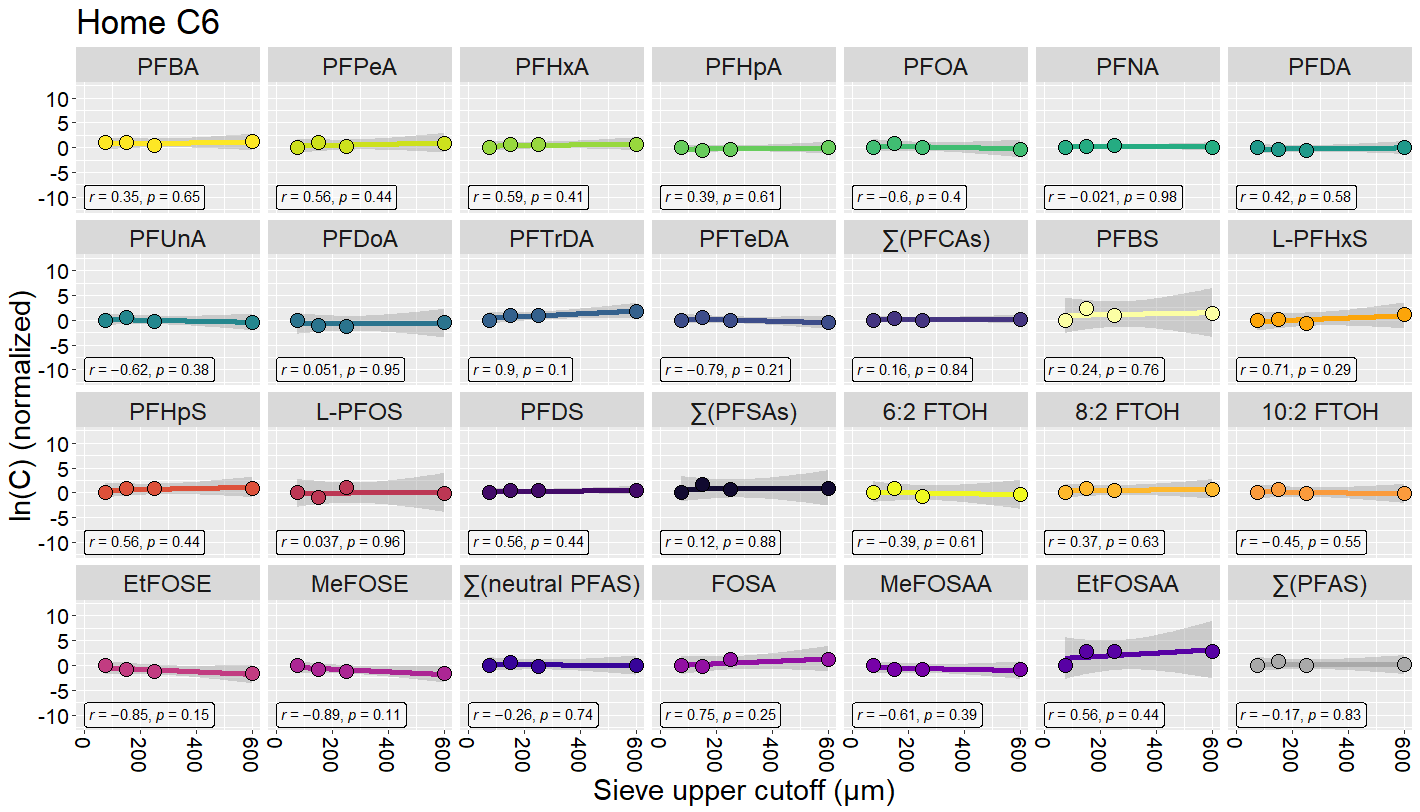


**Figure S12**: Correlations between normalized PFAS concentrations and size fraction found for Home C6 (New York). Concentrations were normalized by the concentrations measured in the 75 µm size fractions. A) Full range of size fractions (<63 µm to <2,000 µm); B) subset of size fractions (<63 µm to <600 µm).

**A**


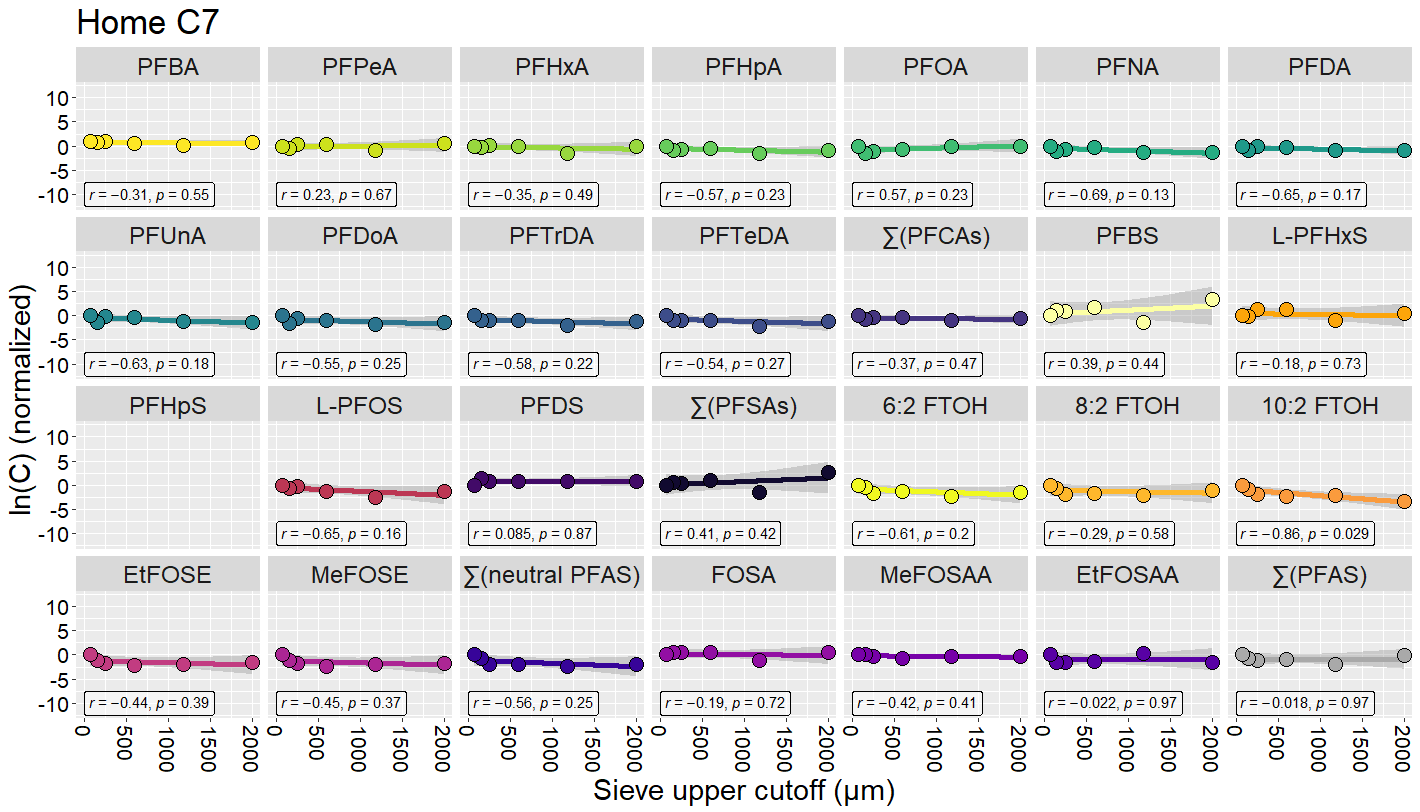


**B**


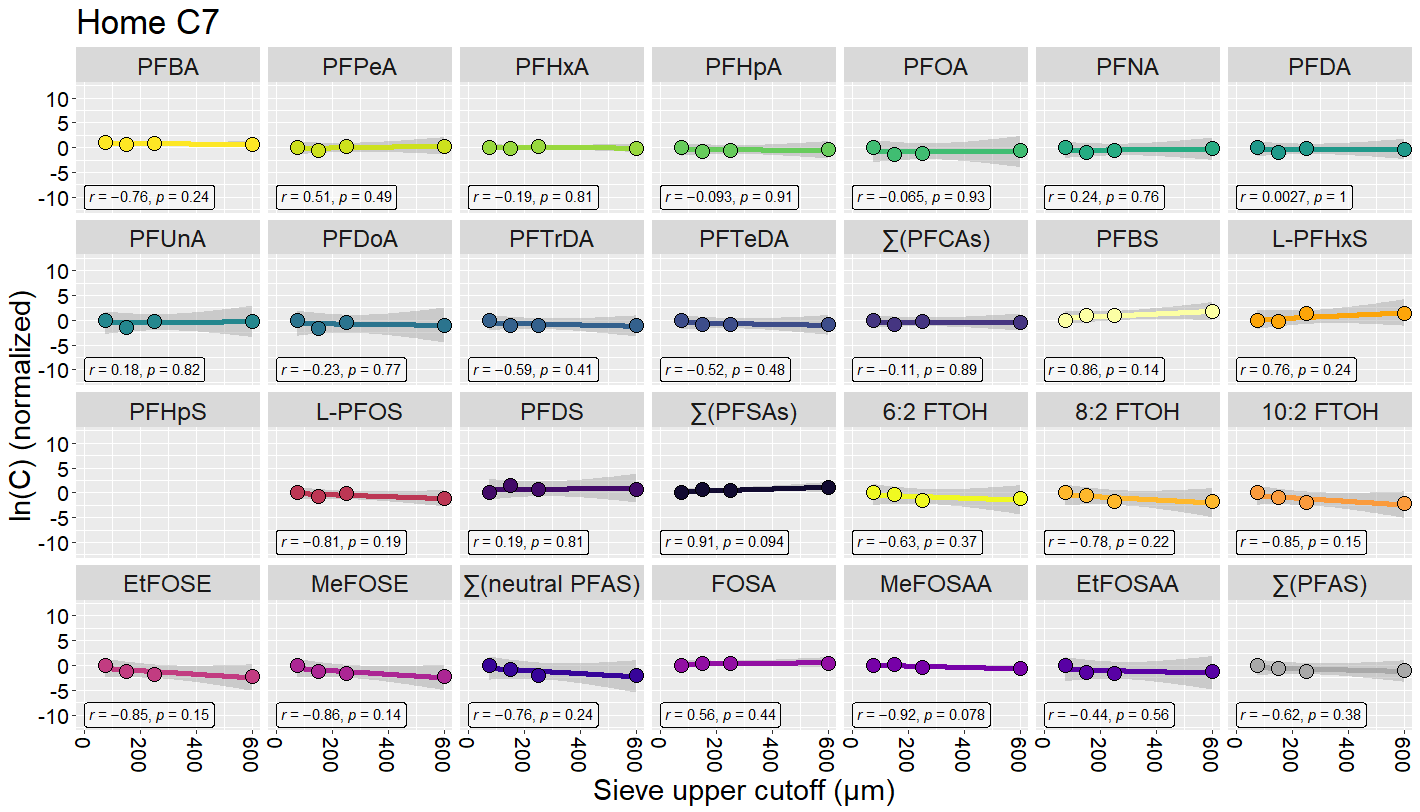


**Figure S13**: Correlations between normalized PFAS concentrations and size fraction found for Home C7 (New York). Concentrations were normalized by the concentrations measured in the 75 µm size fractions. PFHpS was not detected in enough size fractions from Home C7. A) Full range of size fractions (<63 µm to <2,000 µm); B) subset of size fractions (<63 µm to <600 µm).


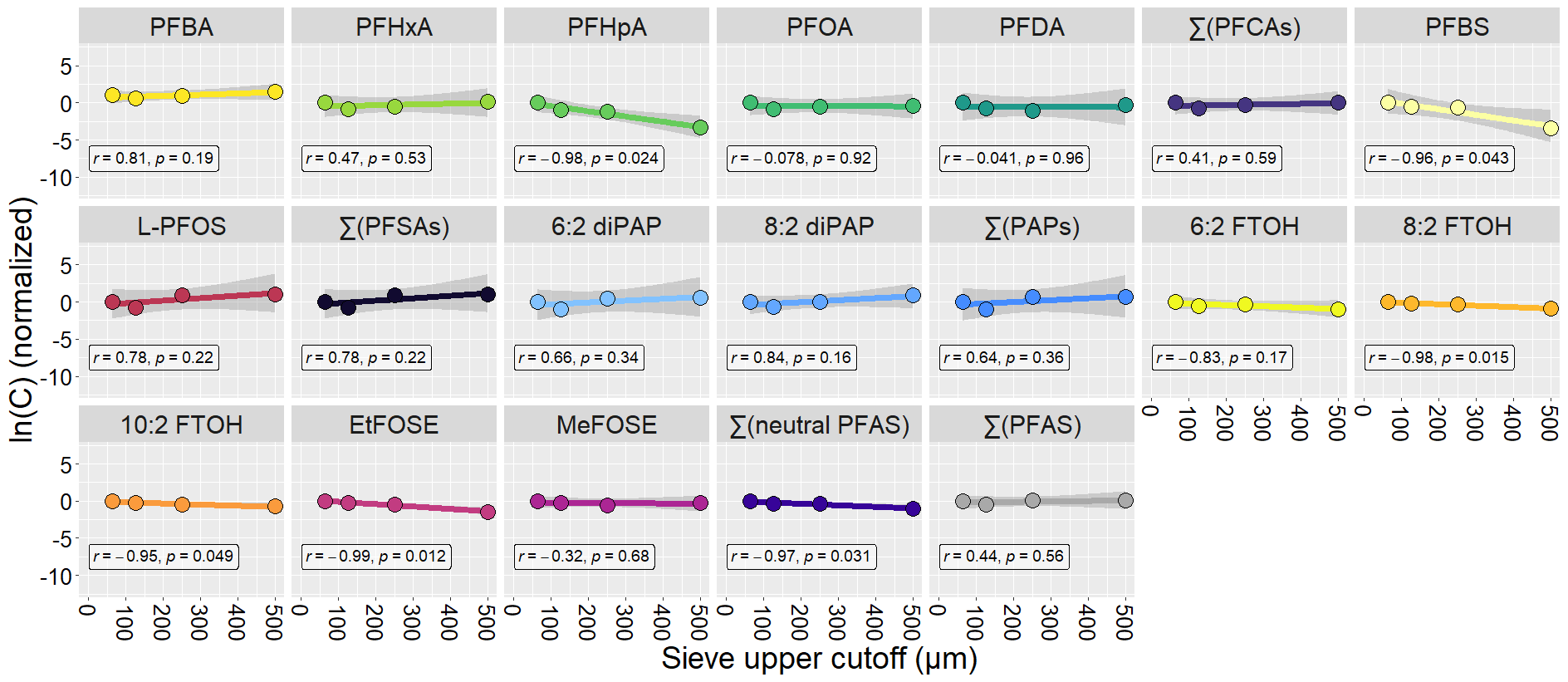


**Figure S14**: Correlations between normalized PFAS concentrations and size fraction found for Home 01 (North Carolina). Concentrations were normalized by the concentrations measured in the 63 µm size fractions.


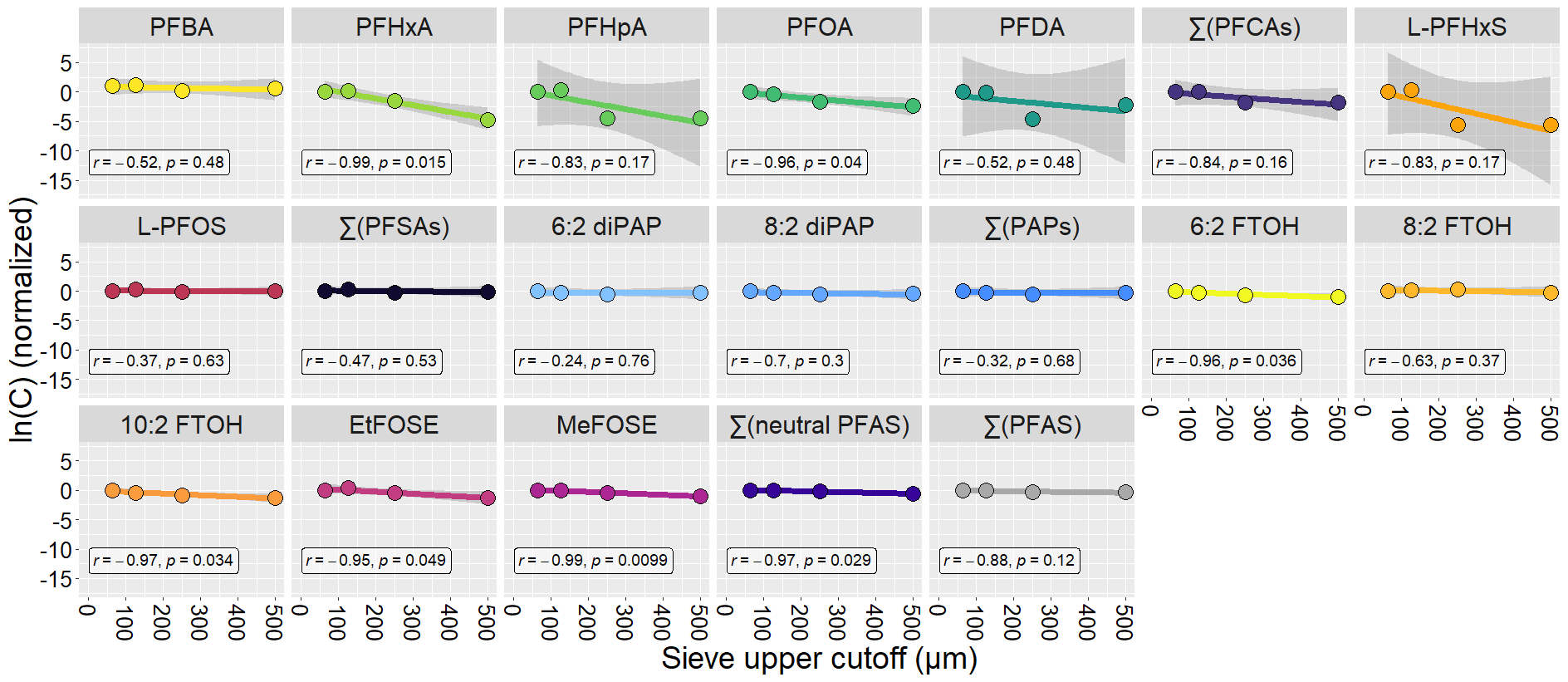


**Figure S15**: Correlations between normalized PFAS concentrations and size fraction found for Home 43 (North Carolina). Concentrations were normalized by the concentrations measured in the 63 µm size fractions.

| **A** | 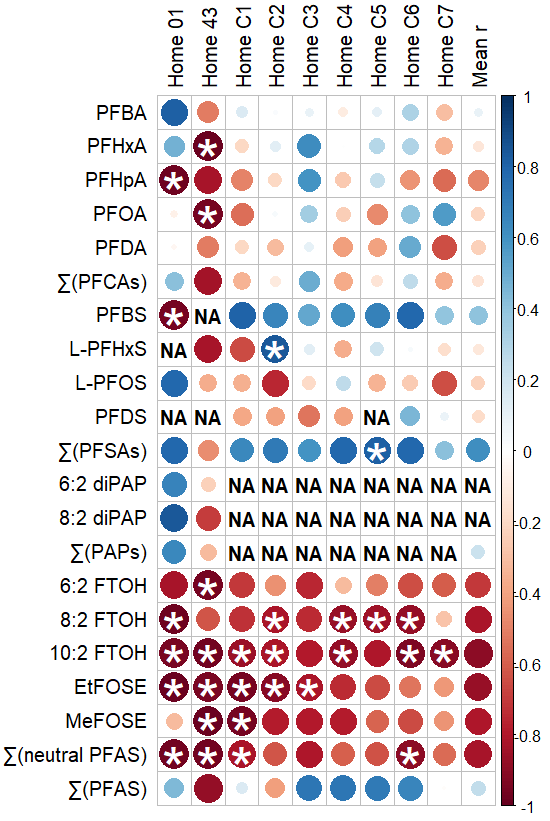 | **B** | 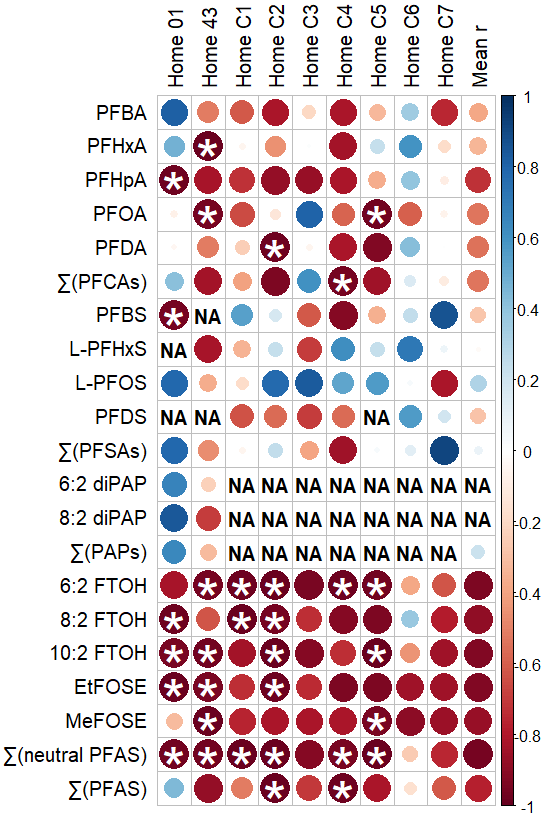 |
| --- | --- | --- | --- |

**Figure S16**: Correlation matrices summarizing the Pearson correlation coefficients (*r*) for the NC homes as shown in **Fig. S14-S15** and A) the full range of size fractions (<63 µm to <2,000 µm) for the NY homes as shown in **Fig. S7A-S13A** and B) the subset of size fractions (<63 µm to <600 µm) for the NY homes as shown in **Fig. S7B-S13B**. Mean *r* values were calculated based on the Fisher-transformed *r* values from all homes (Corey, Dunlap and Burke 1998). The asterisk (*) indicates a significant correlation (p < 0.05). “NA”: not enough data available.

**Table S10**: Dust-air partition coefficients as ${\log(K'}_{d})$ of 6:2 FTOH, 8:2 FTOH, 10:2 FTOH, MeFOSE, and EtFOSE measured in NC homes for different size fractions

| **Home ID** | **Size fraction** | **log(m³/µg)** | | | | |
| --- | --- | --- | --- | --- | --- | --- |
|  |  | **6:2 FTOH** | **8:2 FTOH** | **10:2 FTOH** | **MeFOSE** | **EtFOSE** |
| 01 | 250-500 µm | -5.45 | -5.64 | -5.40 | -4.82 | -4.23 |
| 01 | 125-250 µm | -5.16 | -5.39 | -5.30 | NA | NA |
| 01 | 63-125 µm | -5.28 | -5.35 | -5.20 | -4.83 | -3.71 |
| 01 | <63 µm | -5.03 | -5.24 | -5.08 | -4.73 | -3.61 |
| 10 | 250-500 µm | -5.22 | -5.01 | -4.75 | -5.33 | -3.81 |
| 10 | <250 µm | -4.93 | -4.60 | -4.41 | -5.22 | -3.55 |
| 18 | 250-500 µm | -4.25 | -4.74 | -4.38 | -4.13 | -3.26 |
| 18 | <250 µm | -4.01 | -4.66 | -4.26 | -3.92 | -3.03 |
| 30 | 250-500 µm | -4.54 | -4.88 | -4.57 | NA | NA |
| 30 | <250 µm | -3.93 | -4.62 | -4.60 | NA | NA |
| 35 | 250-500 µm | -5.31 | -5.62 | -5.17 | -4.75 | -4.53 |
| 35 | <250 µm | -5.25 | -5.08 | -4.84 | -4.42 | -3.90 |
| 43 | 250-500 µm | -5.85 | -5.11 | -5.48 | -5.60 | -5.39 |
| 43 | 125-250 µm | -5.70 | -4.87 | -5.27 | -5.31 | -5.02 |
| 43 | 63-125 µm | -5.54 | -4.91 | -5.09 | -5.13 | -4.68 |
| 43 | <63 µm | -5.41 | -4.99 | -4.91 | -5.14 | -4.81 |
| 50 | 250-500 µm | -3.87 | -5.12 | -4.68 | -3.87 | -4.23 |
| 50 | <250 µm | -3.51 | -4.66 | -4.30 | -3.83 | -4.04 |
| 59 | 250-500 µm | -4.74 | -5.41 | -5.02 | -5.28 | -2.66 |
| 59 | <250 µm | -4.66 | -5.16 | -4.82 | -5.01 | -4.06 |
| 65 | 250-500 µm | -5.44 | -5.21 | -4.77 | -5.06 | -5.03 |
| 65 | <250 µm | -5.20 | -5.03 | -4.63 | -4.67 | -4.21 |
| 78 | 250-500 µm | -4.63 | -4.95 | -4.25 | -4.85 | -4.84 |
| 78 | <250 µm | -4.09 | -4.35 | -3.70 | -4.95 | -4.79 |


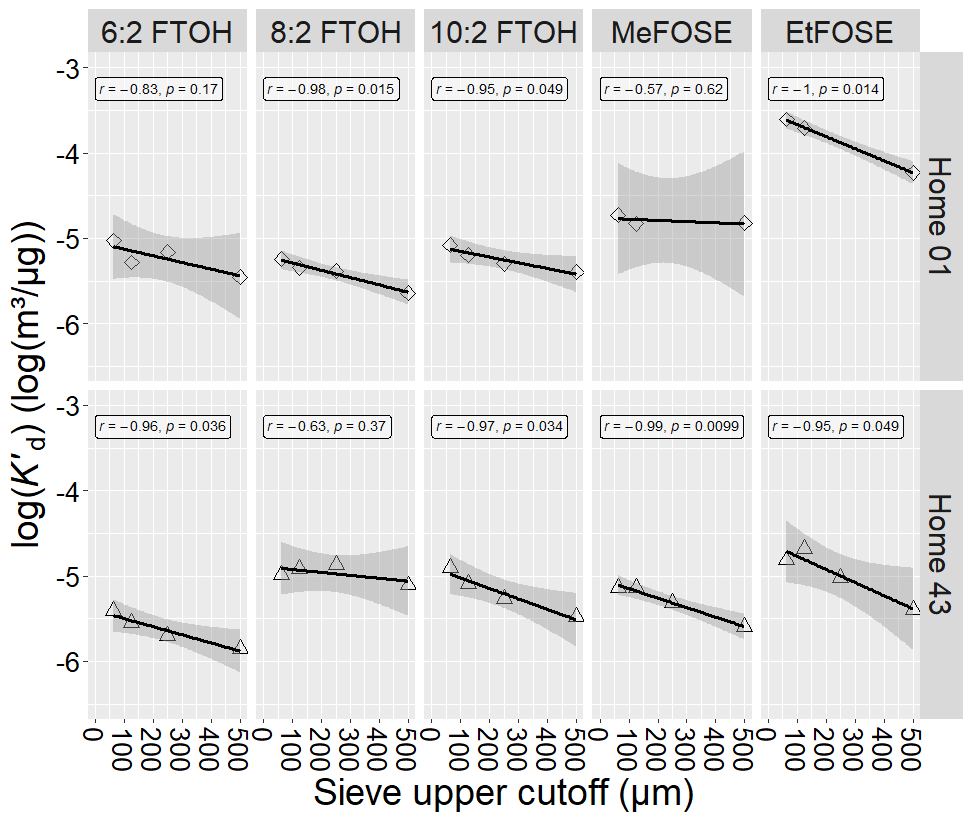


**Figure S17**: Correlations between the dust-air partition coefficient, ${\log(K'}_{d})$, and the dust size fraction for 6:2 FTOH, 8:2 FTOH, 10:2 FTOH, MeFOSE, and EtFOSE, based on data from North Carolina homes 01 and 43.

**References**

Chang, N. Y., C. M. A. Eichler, E. A. Cohen Hubal, J. D. Surratt, G. C. Morrison, B. J. Turpin. 2025. Exposure to Per- and Polyfluoroalkyl Substances (PFAS) in North Carolina Homes: Results from the Indoor PFAS Assessment (IPA) Campaign. *Environ. Sci. Process Impacts* 27:1654-1670. doi: <https://doi.org/10.1039/D4EM00525B>

Corey, D. M., W. P. Dunlap, M. J. Burke. 1998. Averaging Correlations: Expected Values and Bias in Combined Pearson rs and Fisher's z Transformations. *The Journal of General Psychology* 125:245-261. doi: 10.1080/00221309809595548.

Eichler, C. M. A., N. Y. Chang, D. E. Amparo, E. A. Cohen Hubal, J. D. Surratt, G. C. Morrison, B. J. Turpin. 2024. Partitioning of Neutral PFAS in Homes and Release to the Outdoor Environment: Results from the IPA Campaign. *Environ. Sci. Technol.* 58:18870-18880. doi: <https://doi.org/10.1021/acs.est.4c05286>.

Eichler, C. M. A., N. Y. Chang, E. A. Cohen Hubal, D. E. Amparo, J. Zhou, J. D. Surratt, G. C. Morrison, B. J. Turpin. 2023. Cloth-Air Partitioning of Neutral Per- and Polyfluoroalkyl Substances (PFAS) in North Carolina Homes during the Indoor PFAS Assessment (IPA) Campaign *Environ. Sci. Technol.* 57:15173-15183. doi: <https://doi.org/10.1021/acs.est.3c04770>.

USEPA. 2016. Definition and Procedure for the Determination of the Method Detection Limit, Revision 2: U.S. Environmental Protection Agency.

USEPA. 2024. Method 1633, Revision A: Analysis of Per- and Polyfluoroalkyl Substances (PFAS) in Aqueous, Solid, Biosolids, and Tissue Samples by LC-MS/MS: U.S. Environmental Protection Agency.

Zhou, J., K. Baumann, R. N. Mead, S. A. Skrabal, R. J. Kieber, G. B. Avery, M. Shimizu, J. C. DeWitt, M. Sun, S. A. Vance, W. Bodnar, Z. Zhang, L. B. Collins, J. D. Surratt, B. J. Turpin. 2021. PFOS dominates PFAS composition in ambient fine particulate matter (PM2.5) collected across North Carolina nearly 20 years after the end of its US production. *Environ. Sci. Process Impacts* 23:580-587. doi: 10.1039/D0EM00497A.
